# Supplementary material for: Synthesis of D-π-A′-π-A Chromophores with Quinoxaline Core as Auxiliary Acceptor and Effect of Various Silicon-Substituted Donor Moieties on Thermal and Nonlinear Optical Properties at Molecular and Material Level
Source: Molecules. 2023 Jan 5;28(2):531. doi: 10.3390/molecules28020531 (PMC9867400; doi:10.3390/molecules28020531)
Supplement: Supplementary file 1 [file molecules-28-00531-s001.zip › molecules-2130188-supplementary.pdf]

Electronic Supplementary File

Table of Contents

1. The most stable **Chr-An1** conformers (Fig. S1**)** and **DBA-VQV-TCF$_{\mathbf{CyPh}}$** chromophore (Fig. S2**)** **2**

2. Notation of dihedral angles between donor/acceptor and bridge planes (Fig. S3) **3**

3. **Chr-An3/PMMA**(40) in the amorphous cell (Fig. S4) **4**

4. UV–Vis electron absorption spectra for films before and after poling (Fig. S5) **5**

5. Polymer films characteristics (Table S1) **6**

6. The coordinates (x,y,z) of the chromophores **Chr-An1, Chr-An2, Chr-An3, Chr-Car, Chr-PT, Chr-TQ**(Table S2) **7**

7. ^{1}H and ^{13}C NMR spectra **19**

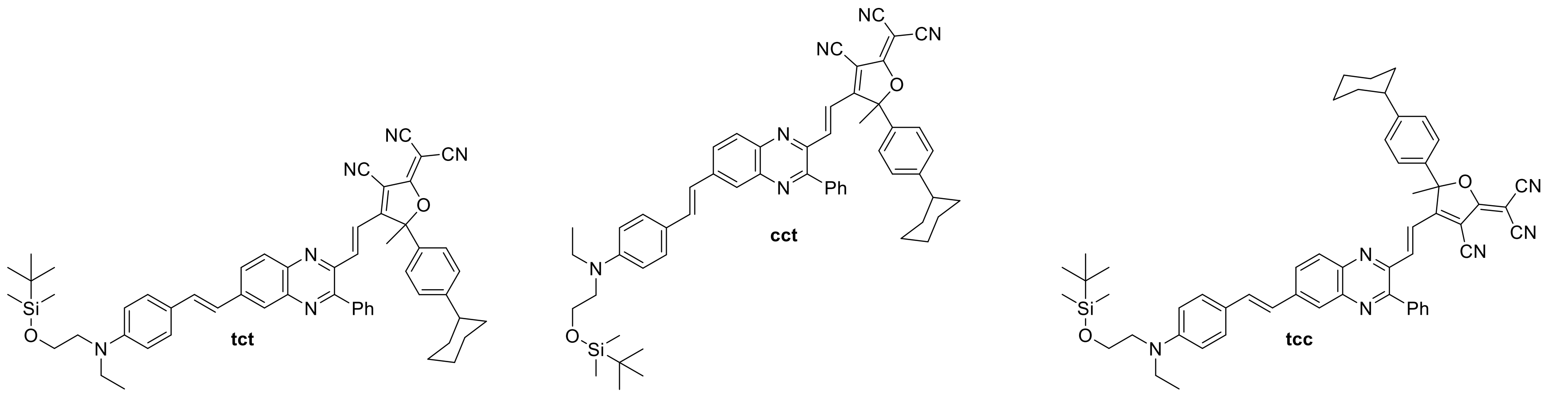

Figure S1. The most stable **Chr-An1** conformers **a-c** with Boltzmann weight factors estimated by Conformational search being 83 (*tct*), 10 (*cct*), 5 (*tcc*)%.

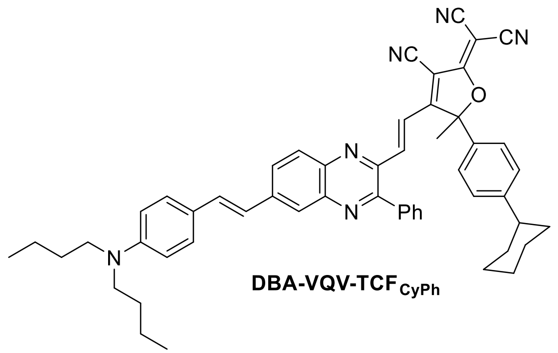

Figure S2. DBA-VQV-TCF$_{\mathbf{CyPh}}$ chromophore.

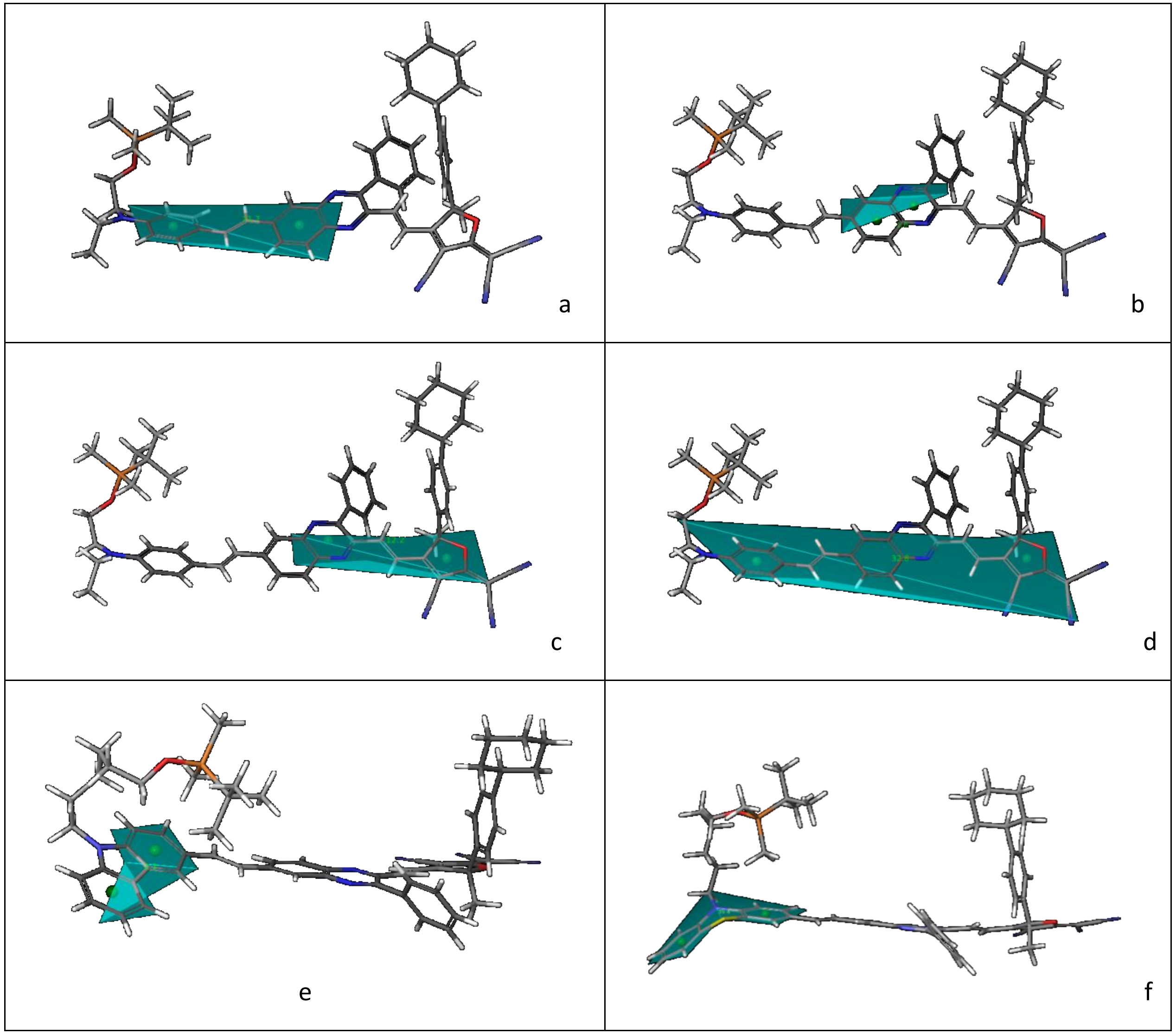

Figure S3. Dihedral angles between donor and bridge planes (a), between bridge fused cycles planes (b), between bridge cycle and acceptor (c), between donor and acceptor planes (d) for **Chr-An1** chromophore; between donor planes for **Chr-Car** (e) and **Chr-PT** (f) chromophores.

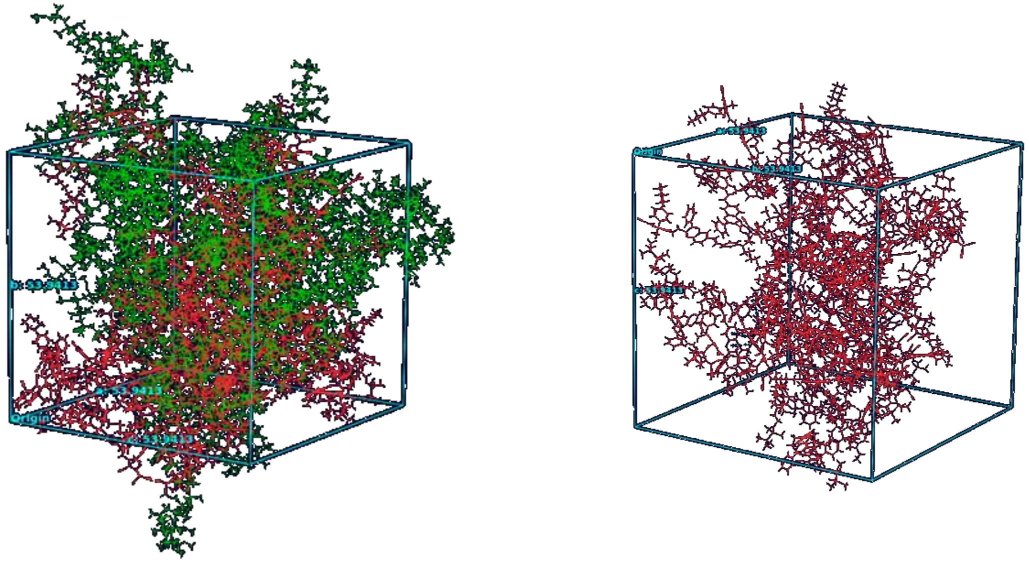

Figure S4. Chr-An3/PMMA(40) in the amorphous cell

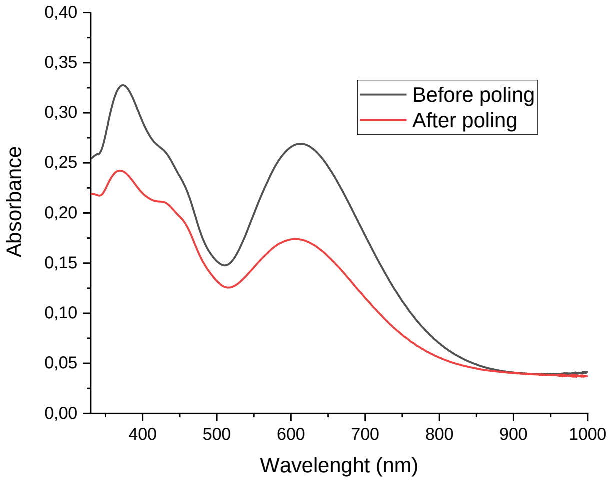
Absorbance
0,00
0,05
0,10
0,15
0,20
0,25
0,30
0,35
0,40
400
500
600
700
800
900
1000
Wavelenght (nm)
Before poling
After poling

a

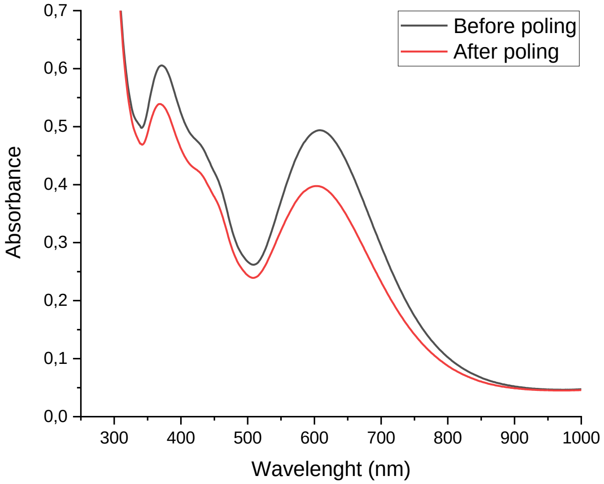
Absorbance
0,0
0,1
0,2
0,3
0,4
0,5
0,6
0,7
300
400
500
600
700
800
900
1000
Wavelenght (nm)
Before poling
After poling

b

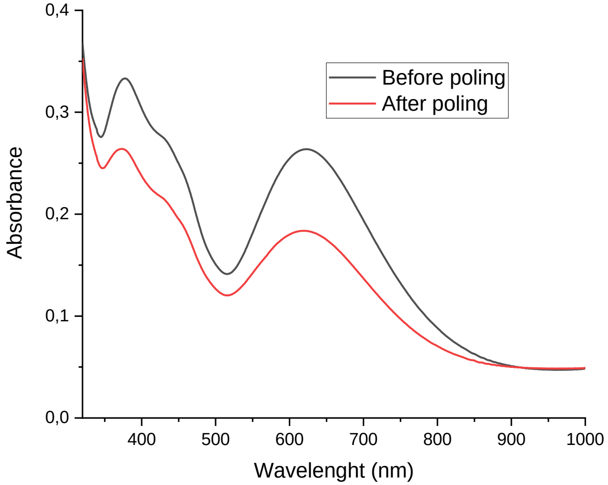
Absorbance
0,0
0,1
0,2
0,3
0,4
400
500
600
700
800
900
1000
Wavelenght (nm)
Before poling
After poling

c

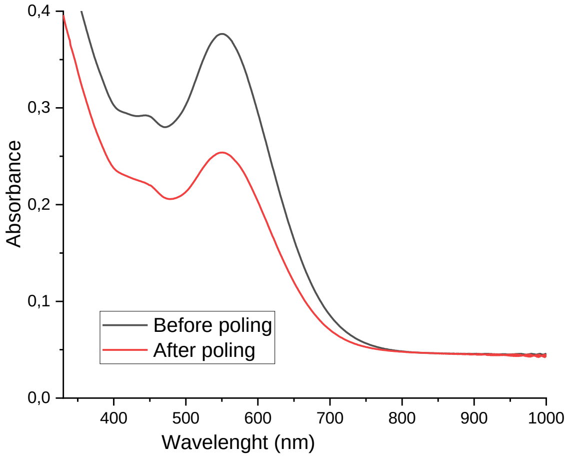
Absorbance
0,0
0,1
0,2
0,3
0,4
400
500
600
700
800
900
1000
Wavelenght (nm)
Before poling
After poling

d

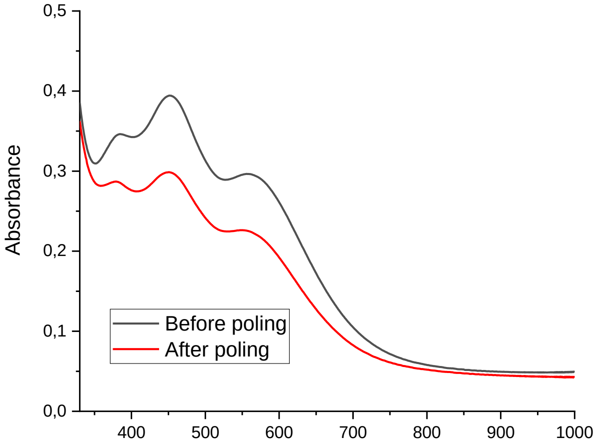
Absorbance
0,0
0,1
0,2
0,3
0,4
0,5
400
500
600
700
800
900
1000
Before poling
After poling

e

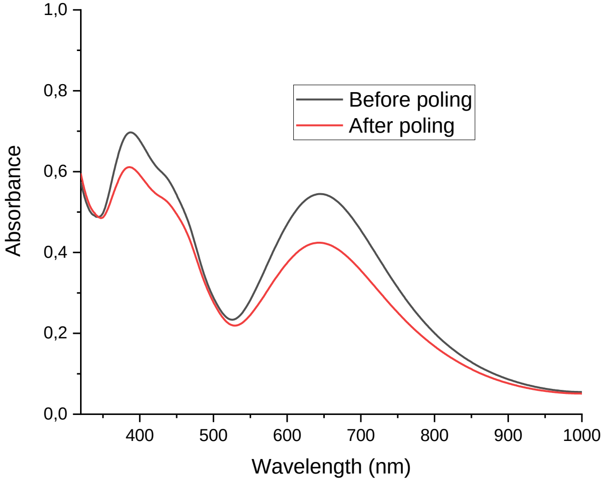
Absorbance
0,0
0,2
0,4
0,6
0,8
1,0
400
500
600
700
800
900
1000
Wavelength (nm)
Before poling
After poling

f

Figure S5. UV–vis electronic absorption spectra registered before and after poling for films **Chr-An1/PMMA** (a), **Chr-An2/PMMA** (b), **Chr-An3/PMMA** (c), **Chr-Car/PMMA** (d), **Chr-PT/PMMA** (e), **Chr-TQ/PMMA** (f) with chromophore load 25 wt. %

Table S1. Polymer films characteristics: poling temperature, T_p, order parameter, η, film thickness, and λ_{max}.

Polymer films	T_p, °C	η	h, nm	λ_{max}, nm
**Chr-An1(25)/PMMA**	110	0.35	240	612
**Chr-An1(40)/PMMA**	110	0.17	800	612
**Chr-An2(25)/PMMA**	100	0.23	640	607
**Chr-An2(40)/PMMA**	110	0.20	830	608
**Chr-An3(25)/PMMA**	110	0.35	290	624
**Chr-An3(40)/PMMA**	110	0.33	250	626
**Chr-Car(25)/PMMA**	110	0.35	400	551
**Chr-Car(40)/PMMA**	100	0.24	800	553
**Chr-PT(25)/PMMA**	110	0.23	400	556
**Chr-PT(40)/PMMA**	110	0.10	1000	562
**Chr-TQ(25)/PMMA**	90	0.24	600	645

Table S2. The coordinates (x,y,z) of the chromophores **Chr-An1, Chr-An2, Chr-An3, Chr-Car, Chr-PT, Chr-TQ**

**Chr_An1**				**Chr_An2**			
C	19.08380	6.12580	7.93400	C	0.63380	-5.24560	0.41580
C	20.38890	5.61010	7.64190	C	0.33010	-5.49550	-0.96310
N	18.91930	7.17780	8.75780	N	0.96850	-4.01570	0.83780
C	20.00880	7.70520	9.31800	C	0.96200	-3.01560	-0.04610
C	21.31700	7.13170	9.09000	C	0.57190	-3.24940	-1.41960
N	21.49140	6.13270	8.24460	N	0.30810	-4.46950	-1.85770
C	20.53210	4.53460	6.74410	C	0.00850	-6.80290	-1.37940
C	19.43070	3.94700	6.12680	C	-0.05860	-7.85700	-0.47050
C	18.13010	4.47120	6.43800	C	0.26390	-7.58760	0.90430
C	17.95920	5.51760	7.30510	C	0.59940	-6.33350	1.33510
H	21.53770	4.17170	6.55110	H	-0.22760	-6.95690	-2.42850
C	19.64640	2.83690	5.20870	C	-0.47130	-9.17510	-0.92790
H	17.25550	4.01790	5.98240	H	0.25680	-8.40470	1.61810
H	16.97270	5.90880	7.53660	H	0.84970	-6.13280	2.37220
C	18.73120	2.24460	4.40210	C	-0.83300	-10.21480	-0.13510
H	17.71120	2.62900	4.39780	H	-0.82310	-10.06700	0.94400
H	20.67740	2.49060	5.16230	H	-0.54240	-9.28510	-2.00840
C	19.39950	-0.88360	1.48320	C	-2.36560	-14.09650	-1.24950
C	20.35720	-0.60880	2.49290	C	-2.39150	-13.62720	0.08430
C	20.14210	0.37150	3.44500	C	-1.86270	-12.38930	0.41430
C	18.96840	1.15310	3.46700	C	-1.30390	-11.52520	-0.54730
C	18.00340	0.85000	2.48820	C	-1.26360	-12.00590	-1.87240
C	18.20100	-0.13000	1.52620	C	-1.76220	-13.25140	-2.21610
H	21.30440	-1.13180	2.49440	H	-2.88010	-14.20870	0.85480
H	20.91520	0.53830	4.19030	H	-1.91570	-12.06110	1.45050
H	17.07050	1.41060	2.47440	H	-0.80320	-11.40400	-2.65130
H	17.40960	-0.31160	0.80880	H	-1.64080	-13.59900	-3.23390
C	19.85510	8.91130	10.11920	C	1.41170	-1.71100	0.42630
C	18.66760	9.39350	10.56900	C	1.71910	-1.46270	1.72870
H	17.75960	8.84770	10.32340	H	1.63210	-2.28480	2.43480
H	20.76870	9.44920	10.33890	H	1.52870	-0.93410	-0.31980

C	18.51490	10.56910	11.37090	C	2.15340	-0.20940	2.26300
C	17.31220	11.04710	11.83680	C	2.54130	-0.00240	3.56970
C	17.55100	12.22880	12.64110	C	2.87480	1.39840	3.74260
O	18.86620	12.48720	12.66880	O	2.71000	2.04240	2.57710
C	19.62030	11.48410	11.89300	C	2.20140	1.12990	1.53650
C	16.68180	13.05130	13.32250	C	3.30200	2.10020	4.84920
C	20.52790	10.79460	12.91810	C	0.79180	1.63510	1.20380
C	16.05910	10.44610	11.55720	C	2.58840	-1.02100	4.55980
N	15.07540	9.89130	11.27810	N	2.59880	-1.91060	5.31010
H	21.16930	11.53980	13.39640	H	0.84140	2.66670	0.84630
H	19.91080	10.32330	13.68850	H	0.31630	1.01170	0.44280
H	21.15460	10.02730	12.45920	H	0.17430	1.60970	2.10560
C	17.18980	14.16460	14.05410	C	3.55270	3.50170	4.74320
C	15.27240	12.84680	13.33720	C	3.50740	1.48730	6.11910
N	17.58320	15.07750	14.65990	N	3.75810	4.64540	4.67030
N	14.11470	12.72890	13.38130	N	3.68930	1.03000	7.17470
C	24.87700	8.43640	11.10650	C	0.11070	-0.16120	-4.37560
C	24.86530	8.28060	9.71880	C	0.83660	-1.31890	-4.66510
C	23.70640	7.86070	9.06980	C	0.98780	-2.31190	-3.69990
C	22.53440	7.61020	9.79790	C	0.43200	-2.15480	-2.42120
C	22.55830	7.75710	11.19350	C	-0.30660	-0.99350	-2.14390
C	23.72330	8.16430	11.84390	C	-0.46860	-0.00530	-3.11560
H	25.78380	8.75850	11.61210	H	-0.01350	0.60900	-5.13210
H	25.76320	8.48400	9.14060	H	1.28170	-1.45040	-5.64770
H	23.69530	7.72260	7.99290	H	1.53530	-3.22170	-3.92600
H	21.67110	7.51890	11.77500	H	-0.79030	-0.88120	-1.17670
H	23.73170	8.25680	12.92700	H	-1.06020	0.87870	-2.89240
C	20.72870	-2.78740	0.56320	C	-3.20070	-15.68680	-2.97980
N	19.62800	-1.83190	0.49870	N	-2.90050	-15.33050	-1.59760
C	18.69580	-2.01410	-0.61220	C	-3.23780	-16.34200	-0.60230
H	19.27070	-2.39450	-1.46490	H	-2.62360	-16.19410	0.28920
C	17.53590	-2.97360	-0.31270	C	-4.71410	-16.42210	-0.19900
H	18.31580	-1.03590	-0.92370	H	-2.95960	-17.32550	-1.00640
H	20.36260	-3.75770	0.19940	H	-3.31780	-14.77660	-3.57220

C	21.95760	-2.40970	-0.27210	C	-2.19090	-16.61470	-3.66240
H	21.02760	-2.94440	1.60260	H	-4.17550	-16.19510	-3.00270
H	16.88910	-3.07590	-1.19200	O	-5.10440	-15.28090	0.53990
H	17.90800	-3.97070	-0.05080	H	-5.33580	-16.53480	-1.10090
H	16.92690	-2.61390	0.52230	H	-2.05910	-17.52300	-3.05380
H	22.63890	-3.27390	-0.29150	H	-2.62350	-16.93080	-4.62500
H	21.64150	-2.22540	-1.31050	O	-0.95350	-15.96000	-3.86800
O	22.63470	-1.29360	0.27560	Si	0.46880	-16.77440	-4.28060
Si	23.18240	0.03410	-0.61280	C	1.78240	-15.40960	-4.51520
C	24.29020	1.03130	0.58320	C	0.18520	-17.75930	-5.87110
C	24.88290	2.25020	-0.16010	H	-0.61160	-18.50250	-5.74360
C	23.47540	1.53510	1.79310	H	-0.10030	-17.10990	-6.70660
C	25.44290	0.13980	1.09420	H	1.08900	-18.30520	-6.16720
C	21.70360	1.04240	-1.21930	C	2.00990	-14.65560	-3.18730
C	24.16960	-0.57450	-2.10900	C	1.31430	-14.40700	-5.59190
H	22.02440	1.94020	-1.76190	C	3.11540	-16.04700	-4.96640
H	21.07940	0.45640	-1.90530	H	-4.84610	-17.33620	0.40190
H	21.06800	1.35880	-0.38520	Si	-6.70130	-14.95070	0.98370
H	24.53220	0.26740	-2.71120	C	-6.62630	-13.30300	1.94520
H	25.04020	-1.16850	-1.80850	C	-7.76930	-14.80990	-0.57130
H	23.55540	-1.19990	-2.76840	C	-7.35510	-16.36400	2.05830
H	24.12080	2.11600	2.46830	C	-5.67010	-13.43620	3.15000
H	22.64600	2.18670	1.49260	C	-6.12150	-12.17180	1.02310
H	23.05190	0.70620	2.36950	C	-8.03690	-12.94090	2.46150
H	25.52530	2.83130	0.51730	H	-8.39080	-16.18090	2.36840
H	25.50080	1.95430	-1.01700	H	-7.34620	-17.31690	1.51500
H	24.10300	2.92940	-0.52690	H	-6.75420	-16.49560	2.96530
H	26.08020	0.70270	1.79150	H	-8.79890	-14.52980	-0.31790
H	25.06530	-0.74150	1.62510	H	-7.37840	-14.05350	-1.26100
H	26.08600	-0.21070	0.27740	H	-7.82160	-15.76110	-1.11490
C	21.59700	13.80050	8.76460	C	0.93240	-17.96860	-2.88920
C	22.34430	12.99150	9.63010	H	1.88160	-18.47470	-3.10300
C	21.73160	12.22990	10.62680	H	1.04010	-17.44730	-1.93160
C	20.34250	12.24670	10.78280	H	0.17560	-18.75170	-2.75820

C	19.58740	13.04030	9.90960	C	5.12520	1.32630	-1.69860
C	20.20480	13.80250	8.92460	C	5.47470	0.89470	-0.41100
H	23.42700	12.95710	9.54010	C	4.53150	0.81710	0.60900
H	22.35430	11.62830	11.28190	C	3.19660	1.17510	0.37860
H	18.50590	13.07970	10.00680	C	2.83720	1.59930	-0.90500
H	19.59340	14.41880	8.26920	C	3.78640	1.66870	-1.92460
C	23.57860	14.66760	5.51060	H	6.50600	0.61970	-0.20220
C	22.94630	13.79390	6.60550	H	4.84340	0.48580	1.59580
C	22.25120	14.65120	7.68980	H	1.81410	1.88190	-1.12850
C	23.23850	15.68780	8.27670	H	3.47040	2.00480	-2.90830
C	23.86870	16.55800	7.17840	C	7.49160	3.02290	-4.29680
C	24.55110	15.69930	6.10280	C	6.40960	2.90730	-3.21270
H	22.78200	15.19300	4.96280	C	6.17290	1.43740	-2.79220
H	24.09330	14.03350	4.77750	C	5.84450	0.57000	-4.02900
H	23.72620	13.17910	7.07740	C	6.92940	0.68870	-5.11060
H	22.22210	13.09480	6.16820	C	7.16510	2.15060	-5.51780
H	21.44950	15.21590	7.19040	H	8.45910	2.71040	-3.87670
H	22.71980	16.31460	9.01290	H	7.60810	4.07210	-4.59670
H	24.03500	15.16200	8.82300	H	5.46740	3.32880	-3.59200
H	23.08620	17.17370	6.71020	H	6.68330	3.50260	-2.33280
H	24.58840	17.25710	7.62330	H	7.12010	1.06310	-2.37600
H	25.40760	15.17290	6.55080	H	5.72100	-0.47660	-3.72350
H	24.95920	16.33730	5.30870	H	4.87940	0.88690	-4.44950
				H	7.86910	0.26340	-4.72830
				H	6.65080	0.08840	-5.98610
				H	6.26050	2.53970	-6.00870
				H	7.97290	2.21400	-6.25770
				H	2.75860	-13.86200	-3.32550
				H	1.09120	-14.18570	-2.81990
				H	2.38460	-15.32010	-2.39930
				H	2.06700	-13.61730	-5.72970
				H	1.16720	-14.88950	-6.56610
				H	0.37180	-13.92370	-5.31220
				H	3.88160	-15.26970	-5.09840

				H	3.50340	-16.75990	-4.22820
				H	3.01990	-16.57360	-5.92400
				H	-5.62340	-12.48820	3.70530
				H	-6.00140	-14.20930	3.85430
				H	-4.65180	-13.68630	2.83300
				H	-6.06870	-11.22460	1.57940
				H	-5.12170	-12.38180	0.62840
				H	-6.79270	-12.00930	0.17090
				H	-8.00590	-11.98920	3.01080
				H	-8.75770	-12.81770	1.64360
				H	-8.43400	-13.69850	3.14830
**Chr-An3**				**Chr_Car**			
C	-35.98610	2.64310	-15.03960	C	13.12100	3.19870	-10.28790
C	-36.16680	3.84780	-14.28330	C	14.21780	3.16020	-9.36780
N	-34.89570	1.87690	-14.86780	N	11.88060	2.83810	-9.90460
C	-34.00760	2.23950	-13.94000	C	11.70200	2.47790	-8.63410
C	-34.23860	3.40050	-13.10740	C	12.79240	2.52210	-7.68450
N	-35.27470	4.19520	-13.31620	N	14.02060	2.82290	-8.06440
C	-37.29680	4.65490	-14.51920	C	15.51250	3.49980	-9.80970
C	-38.26450	4.29390	-15.45350	C	15.75650	3.87950	-11.12580
C	-38.06420	3.08500	-16.20400	C	14.64340	3.92920	-12.03160
C	-36.96920	2.28790	-16.00850	C	13.37450	3.60020	-11.63120
C	-39.44210	5.13300	-15.60910	H	16.31890	3.45730	-9.08300
C	-40.55160	4.84980	-16.33500	C	17.12220	4.21080	-11.52080
H	-40.59610	3.90490	-16.87520	H	14.80760	4.24850	-13.05620
H	-39.41990	6.05210	-15.02680	H	12.53430	3.63840	-12.31820
C	-32.77130	1.47230	-13.85850	C	17.58690	4.40130	-12.77720
C	-32.53580	0.34200	-14.57810	H	16.90130	4.27680	-13.61400
H	-33.32080	-0.01970	-15.23750	H	17.82890	4.26050	-10.69390
H	-31.99710	1.87830	-13.21970	C	10.39050	1.98120	-8.23730
C	-31.31590	-0.40560	-14.56260	C	9.27420	2.06760	-9.00420
C	-31.04740	-1.50010	-15.35520	H	9.35180	2.53550	-9.98290
C	-29.71070	-1.97720	-15.05780	H	10.34060	1.49440	-7.27200

O	-29.15570	-1.19230	-14.12110	C	7.97940	1.59300	-8.61850
C	-30.10790	-0.16870	-13.66020	C	6.85860	1.65340	-9.41160
C	-28.99140	-3.03680	-15.56580	C	5.73150	1.11380	-8.67680
C	-30.41940	-0.52550	-12.20080	O	6.14160	0.71460	-7.46440
C	-31.95690	-2.04110	-16.30290	C	7.57700	0.98900	-7.27460
N	-32.75910	-2.42170	-17.05500	C	4.40910	0.97150	-9.02850
H	-29.50350	-0.48510	-11.60550	C	7.64020	2.01370	-6.13550
H	-31.15800	0.15640	-11.77280	C	6.84990	2.17040	-10.73190
H	-30.82150	-1.54100	-12.15330	N	6.93240	2.60890	-11.80620
C	-27.67210	-3.29050	-15.08380	H	7.17670	1.59480	-5.23840
C	-29.50910	-3.91100	-16.56390	H	8.66870	2.30110	-5.90620
N	-26.59570	-3.50890	-14.69730	H	7.08610	2.91130	-6.42480
N	-29.89050	-4.65110	-17.37810	C	3.48510	0.40840	-8.10080
C	-31.73550	4.55760	-9.81100	C	3.90020	1.37040	-10.29670
C	-32.14340	5.49930	-10.75890	N	2.71110	-0.04520	-7.35940
C	-32.95070	5.11500	-11.82720	N	3.43030	1.67850	-11.31630
C	-33.34640	3.77740	-11.97610	C	12.30230	1.75510	-3.48850
C	-32.94240	2.84030	-11.01160	C	11.45970	2.66470	-4.13010
C	-32.14500	3.22900	-9.93440	C	11.60430	2.90630	-5.49610
H	-31.11320	4.86070	-8.97310	C	12.59860	2.24730	-6.23510
H	-31.83690	6.53730	-10.66140	C	13.45670	1.35680	-5.57590
H	-33.28570	5.84550	-12.55680	C	13.30230	1.10490	-4.21460
H	-33.28530	1.81120	-11.08430	H	12.18650	1.56170	-2.42520
H	-31.85720	2.49680	-9.18450	H	10.69730	3.19520	-3.56540
C	-28.27560	3.73410	-14.33250	H	10.96330	3.63510	-5.98660
C	-28.40600	2.79130	-15.36200	H	14.24180	0.86540	-6.14270
C	-28.98670	1.54690	-15.13720	H	13.96570	0.40180	-3.71780
C	-29.45190	1.19490	-13.86410	C	9.44240	-2.88920	-6.54030
C	-29.30930	2.12230	-12.82630	C	9.57510	-1.84780	-5.61330
C	-28.73750	3.37230	-13.06130	C	8.98790	-0.60050	-5.82980
H	-28.05100	3.03850	-16.35970	C	8.25160	-0.35260	-6.99190
H	-29.07340	0.84460	-15.96160	C	8.11720	-1.38790	-7.92570
H	-29.65370	1.89020	-11.82400	C	8.69930	-2.63030	-7.70010
H	-28.65790	4.07170	-12.23350	H	10.14320	-2.00420	-4.70000

C	-25.83200	6.77330	-14.03230	H	9.11780	0.17460	-5.08100
C	-26.43150	5.39160	-13.72820	H	7.54580	-1.22890	-8.83620
C	-27.67410	5.10210	-14.60120	H	8.57390	-3.41700	-8.44060
C	-28.71900	6.23250	-14.44260	C	10.15080	-6.34220	-4.85230
C	-28.11600	7.61350	-14.74180	C	9.51820	-4.95660	-5.05480
C	-26.87570	7.88950	-13.87940	C	10.07550	-4.25070	-6.31360
H	-25.44260	6.78020	-15.06110	C	11.61970	-4.17520	-6.25260
H	-24.97380	6.96010	-13.37460	C	12.24760	-5.56310	-6.04810
H	-26.71470	5.34750	-12.66670	C	11.68420	-6.25990	-4.79960
H	-25.68130	4.60620	-13.88210	H	9.85050	-7.00150	-5.68020
H	-27.34270	5.11290	-15.65030	H	9.75980	-6.79940	-3.93440
H	-29.57430	6.03770	-15.10180	H	9.71920	-4.33320	-4.17130
H	-29.11040	6.21920	-13.41490	H	8.42700	-5.04030	-5.13260
H	-27.83590	7.66250	-15.80450	H	9.81550	-4.87840	-7.17910
H	-28.87250	8.39290	-14.58450	H	12.00260	-3.71230	-7.17100
H	-27.17740	7.96040	-12.82360	H	11.91700	-3.51550	-5.42430
H	-26.43690	8.85980	-14.14400	H	12.04490	-6.18480	-6.93280
C	-44.12890	5.77700	-17.03590	H	13.33890	-5.47200	-5.97630
C	-42.91900	5.10110	-17.00680	H	11.98540	-5.69580	-3.90380
C	-41.75960	5.65200	-16.42950	H	12.11610	-7.26310	-4.69600
C	-41.87490	6.96870	-15.93850	C	19.91680	5.20430	-12.25490
C	-43.07310	7.65920	-15.96150	C	18.96580	4.70570	-13.15920
C	-44.26020	7.07130	-16.47310	C	19.36760	4.46700	-14.49750
N	-45.47480	7.72710	-16.41810	C	20.67160	4.65880	-14.93470
C	-46.68130	7.13010	-16.99110	C	21.61280	5.12660	-14.01020
H	-42.87340	4.10000	-17.43080	N	22.96270	5.41070	-14.16850
H	-41.00290	7.47110	-15.52930	C	23.45920	5.88600	-12.95450
H	-43.09050	8.66810	-15.56900	C	24.75260	6.30770	-12.62930
C	-45.61170	9.03580	-15.77400	C	24.98370	6.74700	-11.32760
H	-36.81480	1.37840	-16.58100	C	23.95820	6.76860	-10.36800
H	-38.79540	2.79990	-16.95360	C	22.67180	6.35260	-10.69730
H	-37.40710	5.55680	-13.92380	C	22.41340	5.90780	-11.99820
H	-44.85210	9.72550	-16.16530	C	21.22910	5.41700	-12.67010
H	-46.57640	9.44290	-16.09260	H	19.62850	5.44030	-11.23500

C	-45.54870	9.02050	-14.23300	H	18.62740	4.09720	-15.20310
C	-46.48540	8.01200	-13.55750	H	20.93990	4.44540	-15.96470
H	-44.52390	8.80660	-13.90770	H	25.55520	6.29980	-13.36030
H	-45.77360	10.03910	-13.88580	H	25.98080	7.07920	-11.05080
C	-46.34000	8.02100	-12.02840	H	24.17420	7.11470	-9.36110
H	-47.53100	8.21960	-13.83170	H	21.87850	6.37340	-9.95450
H	-46.25400	7.01270	-13.94600	C	23.78970	5.11120	-15.33260
C	-47.08530	6.89230	-11.29880	C	24.69830	3.88170	-15.14170
H	-45.27720	7.94970	-11.76740	H	23.12210	4.96830	-16.18760
H	-46.69070	8.98690	-11.63950	H	24.39970	5.99570	-15.55490
C	-46.54020	5.49060	-11.57940	C	23.93860	2.55870	-14.96440
H	-47.02590	7.06990	-10.21740	H	25.36370	3.81640	-16.01400
H	-48.15280	6.90300	-11.56320	H	25.34580	4.06330	-14.27610
O	-45.16060	5.43690	-11.24130	C	24.82660	1.36440	-14.56490
H	-47.10920	4.75720	-10.98790	H	23.14660	2.69040	-14.21780
H	-46.68060	5.23000	-12.63930	H	23.42400	2.31590	-15.90500
Si	-44.16000	4.09950	-11.44640	C	25.46600	1.44700	-13.16620
C	-42.41470	4.72920	-10.98570	H	24.22500	0.44900	-14.61280
C	-44.72330	2.69960	-10.30420	H	25.62630	1.24340	-15.30930
C	-44.24180	3.49300	-13.23630	C	24.47340	1.47840	-12.00230
H	-44.06470	1.82640	-10.38690	H	26.12090	0.57760	-13.02610
H	-44.73280	3.01290	-9.25420	H	26.10950	2.33390	-13.08400
H	-45.73640	2.36400	-10.55790	O	23.75440	0.25340	-11.93960
H	-43.56520	2.64370	-13.39140	H	25.02620	1.61820	-11.06210
H	-45.25110	3.14880	-13.49400	H	23.79170	2.33520	-12.10150
H	-43.96420	4.27370	-13.95320	Si	22.15030	0.00490	-11.51030
C	-42.03340	5.92530	-11.88390	C	21.80480	0.50940	-9.68720
C	-42.39550	5.18500	-9.51100	C	21.00470	0.95570	-12.67770
C	-41.37690	3.60270	-11.17990	C	21.91010	-1.84820	-11.75370
H	-41.03170	6.29520	-11.61870	C	20.35540	0.12030	-9.31590
H	-42.01110	5.65290	-12.94580	C	22.77840	-0.22840	-8.74420
H	-42.73740	6.75650	-11.76610	C	21.96880	2.03220	-9.48730
H	-41.40210	5.57510	-9.24480	H	20.14260	0.39290	-8.27140
H	-43.12670	5.97940	-9.32450	H	19.61610	0.63610	-9.94140

H	-42.61440	4.35860	-8.82380	H	20.17920	-0.95830	-9.41080
H	-40.37410	3.95650	-10.89990	H	22.59310	0.05720	-7.69800
H	-41.59560	2.72620	-10.55720	H	22.66460	-1.31740	-8.80930
H	-41.32140	3.26920	-12.22330	H	23.82430	0.00890	-8.97170
H	-46.70690	6.06010	-16.75590	H	21.72980	2.30870	-8.44910
H	-44.97830	5.28660	-17.49510	H	22.99410	2.36800	-9.68110
H	-47.54030	7.56680	-16.47370	H	21.30150	2.60920	-10.13930
C	-46.82260	7.35080	-18.50250	H	19.95170	0.74510	-12.45410
H	-46.86880	8.42020	-18.73640	H	21.13430	2.04250	-12.62250
H	-45.97420	6.92460	-19.04720	H	21.18470	0.65340	-13.71630
H	-47.74050	6.88040	-18.87410	H	20.87530	-2.15360	-11.55950
				H	22.15110	-2.12700	-12.78610
				H	22.56330	-2.43060	-11.09450
**Chr-PT**				**Chr-TQ**			
C	-34.03200	-21.06890	-19.39240	C	-20.68790	-1.88600	-28.49730
C	-33.47180	-20.00290	-20.16530	C	-19.48090	-1.98000	-27.73900
N	-35.33540	-21.06530	-19.05870	N	-21.33430	-0.71890	-28.61670
C	-36.09910	-20.06980	-19.50280	C	-20.86640	0.34380	-27.96060
C	-35.55960	-19.05400	-20.37780	C	-19.72380	0.22170	-27.09760
N	-34.26950	-19.01520	-20.65640	N	-19.03520	-0.90600	-27.03880
C	-32.09090	-19.98370	-20.43590	C	-18.76930	-3.19650	-27.69820
C	-31.25640	-21.00050	-19.98220	C	-19.23810	-4.33120	-28.35450
C	-31.83880	-22.06580	-19.22150	C	-20.46680	-4.22240	-29.09210
C	-33.17740	-22.10810	-18.93400	C	-21.16630	-3.04930	-29.16580
C	-29.81880	-21.04590	-20.22860	H	-17.85090	-3.22640	-27.11850
C	-29.11770	-20.22350	-21.04220	C	-18.47540	-5.56780	-28.28020
H	-29.65990	-19.45900	-21.59680	H	-20.83800	-5.09010	-29.62850
H	-29.29230	-21.83850	-19.70000	H	-22.08310	-2.96700	-29.74170
C	-37.46660	-19.99850	-19.00450	C	-18.88410	-6.79610	-28.68470
C	-38.05670	-21.00010	-18.30160	H	-19.89600	-6.90640	-29.07300
H	-37.48690	-21.90940	-18.12820	H	-17.49180	-5.46720	-27.82480
H	-37.99440	-19.06910	-19.17840	C	-21.50620	1.62140	-28.23220
C	-39.37220	-20.95280	-17.74260	C	-22.55450	1.74470	-29.08900

C	-39.94100	-21.95560	-16.99050	H	-22.93660	0.83930	-29.55300
C	-41.27380	-21.54220	-16.59230	H	-21.09640	2.49380	-27.73910
O	-41.51850	-20.31180	-17.06870	C	-23.19940	2.96450	-29.44970
C	-40.39010	-19.82600	-17.88140	C	-24.25880	3.05600	-30.31990
C	-42.23960	-22.18720	-15.85170	C	-24.63840	4.44510	-30.45130
C	-40.92990	-19.72610	-19.31230	O	-23.84320	5.20180	-29.68220
C	-39.29470	-23.18150	-16.67460	C	-22.83980	4.36920	-28.98910
N	-38.69630	-24.15670	-16.46210	C	-25.62490	5.04300	-31.20290
H	-41.76650	-19.02330	-19.34390	C	-21.48450	4.82590	-29.54600
H	-40.15740	-19.39550	-20.01040	C	-24.84800	1.93990	-30.96550
H	-41.28850	-20.70740	-19.63490	N	-25.27050	0.97010	-31.44920
C	-43.48930	-21.54230	-15.60650	H	-21.36870	5.90320	-29.40430
C	-42.05140	-23.49180	-15.31050	H	-20.65560	4.30810	-29.05740
N	-44.51300	-21.02700	-15.40150	H	-21.44080	4.61620	-30.61810
N	-41.94210	-24.55570	-14.84980	C	-25.78610	6.46030	-31.15480
C	-37.92090	-16.01870	-22.29280	C	-26.50540	4.30700	-32.04450
C	-38.37270	-17.33910	-22.27790	N	-25.92560	7.61580	-31.12510
C	-37.62030	-18.32800	-21.64330	N	-27.24730	3.74680	-32.74530
C	-36.40330	-18.00910	-21.01960	C	-18.18280	3.30880	-24.54930
C	-35.94660	-16.68280	-21.06400	C	-19.56010	3.10420	-24.62110
C	-36.70440	-15.69440	-21.68740	C	-20.08020	2.11920	-25.46000
H	-38.50790	-15.24780	-22.78490	C	-19.22440	1.32360	-26.23700
H	-39.30320	-17.60360	-22.77320	C	-17.83930	1.52450	-26.14060
H	-37.96040	-19.36010	-21.66590	C	-17.32250	2.51440	-25.31070
H	-34.99260	-16.44150	-20.60590	H	-17.77950	4.07860	-23.89630
H	-36.34380	-14.66950	-21.70540	H	-20.23580	3.70520	-24.01960
C	-39.04020	-16.09250	-16.07050	H	-21.15310	1.95010	-25.48300
C	-39.42330	-16.13440	-17.41680	H	-17.17770	0.88950	-26.72110
C	-39.86300	-17.31810	-18.00980	H	-16.24770	2.66500	-25.25350
C	-39.92410	-18.50540	-17.27270	C	-23.56580	5.10920	-24.74550
C	-39.54310	-18.47130	-15.92520	C	-24.41720	4.30090	-25.51140
C	-39.11320	-17.28570	-15.33810	C	-24.16340	4.04870	-26.85570
H	-39.38390	-15.23200	-18.02080	C	-23.04080	4.59520	-27.49080
H	-40.15080	-17.29910	-19.05570	C	-22.18030	5.39510	-26.73200

H	-39.58710	-19.37540	-15.32410
H	-38.82690	-17.28620	-14.28900
C	-39.14840	-12.42810	-14.71520
C	-39.64440	-13.70800	-15.40550
C	-38.55860	-14.80920	-15.41640
C	-37.25750	-14.27510	-16.05990
C	-36.76510	-12.99410	-15.36910
C	-37.85050	-11.90780	-15.35040
H	-38.97140	-12.63780	-13.64990
H	-39.92850	-11.65750	-14.75410
H	-39.93250	-13.47350	-16.44050
H	-40.54740	-14.08530	-14.90990
H	-38.32720	-15.04600	-14.36720
H	-36.48270	-15.05140	-16.02390
H	-37.43980	-14.06670	-17.12410
H	-36.47200	-13.23110	-14.33560
H	-35.86180	-12.62360	-15.87030
H	-38.05670	-11.58470	-16.38180
H	-37.49220	-11.02160	-14.81190
C	-22.07160	-20.69910	-24.07300
C	-21.69550	-20.51220	-25.40350
C	-22.38930	-19.61850	-26.21660
C	-23.47140	-18.91380	-25.68890
C	-23.82630	-19.06830	-24.34900
S	-25.14160	-18.08600	-23.64910
C	-25.78960	-19.30780	-22.52400
C	-27.15190	-19.36280	-22.25910
C	-27.68070	-20.23920	-21.29230
C	-26.77380	-21.08310	-20.62460
C	-25.41600	-21.05670	-20.91020
C	-24.88840	-20.16240	-21.85870
N	-23.51460	-20.10310	-22.15820
C	-23.12620	-19.96080	-23.51530
H	-21.54570	-21.43310	-23.47190

C	-22.44040	5.64590	-25.38460
H	-25.29990	3.86600	-25.04810
H	-24.85520	3.42520	-27.41490
H	-21.29950	5.84010	-27.18250
H	-21.75390	6.28150	-24.83120
C	-24.52630	7.17310	-21.57220
C	-24.17490	6.89630	-23.04200
C	-23.87580	5.39810	-23.28690
C	-22.76470	4.91010	-22.32870
C	-23.11860	5.18800	-20.85940
C	-23.42700	6.67400	-20.62280
H	-25.47340	6.67030	-21.32630
H	-24.69830	8.24700	-21.42670
H	-23.29400	7.49090	-23.32410
H	-24.99130	7.22110	-23.69860
H	-24.78910	4.83970	-23.03250
H	-22.58840	3.83750	-22.48180
H	-21.82290	5.42240	-22.57530
H	-23.99610	4.58630	-20.58030
H	-22.29760	4.85930	-20.20950
H	-22.51250	7.26380	-20.78580
H	-23.72210	6.83870	-19.57890
C	-18.20010	-12.96290	-28.43350
C	-18.89240	-11.76670	-29.08870
C	-18.12150	-10.48780	-28.82880
C	-18.77070	-9.26110	-28.86370
C	-18.12210	-8.03240	-28.63440
C	-16.73980	-8.09210	-28.37400
C	-16.06210	-9.29760	-28.33570
C	-16.72650	-10.53200	-28.54670
N	-16.05370	-11.74280	-28.45890
C	-16.72840	-12.98390	-28.83810
H	-18.67430	-13.90400	-28.73400
H	-18.27270	-12.88960	-27.34140

H	-20.86550	-21.08670	-25.80530	H	-16.64730	-13.16460	-29.92370
H	-22.10380	-19.47870	-27.25500	H	-16.20830	-13.80900	-28.33820
H	-24.03930	-18.22790	-26.31120	H	-18.96960	-11.94490	-30.17190
H	-27.82010	-18.69950	-22.80260	C	-14.59540	-11.79870	-28.40170
H	-27.13040	-21.77970	-19.87200	H	-19.92030	-11.66480	-28.72140
H	-24.75620	-21.74040	-20.38780	H	-19.84000	-9.25430	-29.07190
C	-22.53420	-20.56250	-21.16600	H	-14.99570	-9.27820	-28.14730
H	-33.61440	-22.91230	-18.35020	H	-16.17540	-7.17670	-28.22020
H	-31.18780	-22.86030	-18.86520	H	-14.32610	-12.78180	-27.99720
H	-31.71110	-19.14390	-21.00850	C	-13.89520	-11.59130	-29.75610
H	-22.64210	-21.63890	-20.96240	H	-14.23390	-11.06690	-27.67080
H	-21.54890	-20.42690	-21.61620	C	-12.36530	-11.56740	-29.64190
C	-22.57220	-19.76110	-19.85450	H	-14.20020	-12.39300	-30.44280
C	-22.26650	-18.26900	-20.03560	H	-14.24640	-10.65190	-30.20250
H	-23.54450	-19.87890	-19.36170	C	-11.67570	-11.48170	-31.01090
H	-21.83080	-20.21500	-19.18170	H	-12.06230	-10.71190	-29.01870
C	-22.24070	-17.50040	-18.70700	H	-12.02450	-12.46910	-29.11420
H	-21.29460	-18.15580	-20.53980	C	-10.15640	-11.26150	-30.95920
H	-23.01330	-17.83450	-20.71080	H	-11.88080	-12.40030	-31.57360
C	-21.96120	-15.99620	-18.85600	H	-12.12250	-10.65840	-31.58560
H	-23.19710	-17.62940	-18.18460	C	-9.36940	-12.35170	-30.23350
H	-21.47110	-17.93910	-18.05630	H	-9.77480	-11.18420	-31.98530
C	-23.10230	-15.19570	-19.48290	H	-9.93370	-10.30380	-30.46610
H	-21.75620	-15.56470	-17.86820	O	-9.64290	-13.62180	-30.81490
H	-21.05770	-15.84140	-19.46360	H	-8.29590	-12.11510	-30.29390
O	-24.23920	-15.24260	-18.62730	H	-9.63590	-12.37160	-29.16640
H	-22.78110	-14.15270	-19.62160	Si	-8.67770	-14.98520	-30.61510
H	-23.35180	-15.59470	-20.47750	C	-9.69260	-16.41420	-31.37610
Si	-25.75860	-14.63050	-19.00280	C	-7.03100	-14.73460	-31.51140
C	-26.73050	-14.72700	-17.35890	C	-8.33610	-15.26880	-28.77530
C	-25.60300	-12.84780	-19.61980	C	-10.00870	-16.10390	-32.85460
C	-26.56130	-15.67560	-20.35970	C	-8.89250	-17.73220	-31.29640
H	-26.58890	-12.40290	-19.80180	C	-11.01970	-16.58010	-30.60480
H	-25.07880	-12.21130	-18.89790	H	-6.37260	-15.60330	-31.39050

H	-25.04930	-12.80110	-20.56550	H	-7.17700	-14.57110	-32.58490
H	-27.53830	-15.26970	-20.64950	H	-6.49230	-13.86470	-31.11620
H	-25.93710	-15.69610	-21.26170	H	-7.73400	-16.17170	-28.61690
H	-26.70940	-16.71370	-20.04170	H	-7.77820	-14.42990	-28.34180
C	-26.06440	-13.81480	-16.30620	H	-9.26230	-15.38110	-28.20030
C	-28.18910	-14.26970	-17.57580	H	-10.60860	-16.91350	-33.29510
C	-26.72780	-16.17880	-16.83400	H	-10.57600	-15.17250	-32.95860
H	-26.59780	-13.88440	-15.34670	H	-9.09710	-16.01020	-33.45730
H	-25.02140	-14.10020	-16.12920	H	-9.47490	-18.55890	-31.72820
H	-26.07790	-12.76080	-16.60990	H	-7.94890	-17.67550	-31.85310
H	-28.74800	-14.31460	-16.62970	H	-8.65520	-18.00980	-30.26160
H	-28.24850	-13.23520	-17.93750	H	-11.62120	-17.38740	-31.04780
H	-28.71790	-14.90730	-18.29530	H	-10.85230	-16.83970	-29.55220
H	-27.27410	-16.24190	-15.88130	H	-11.61890	-15.66300	-30.63410
H	-27.21400	-16.86930	-17.53430				
H	-25.70860	-16.54040	-16.65960				

^{1}H NMR ($CDCl_3$, 400 MHz) of 6-(10H-phenothiazin-10-yl)hexan-1-ol

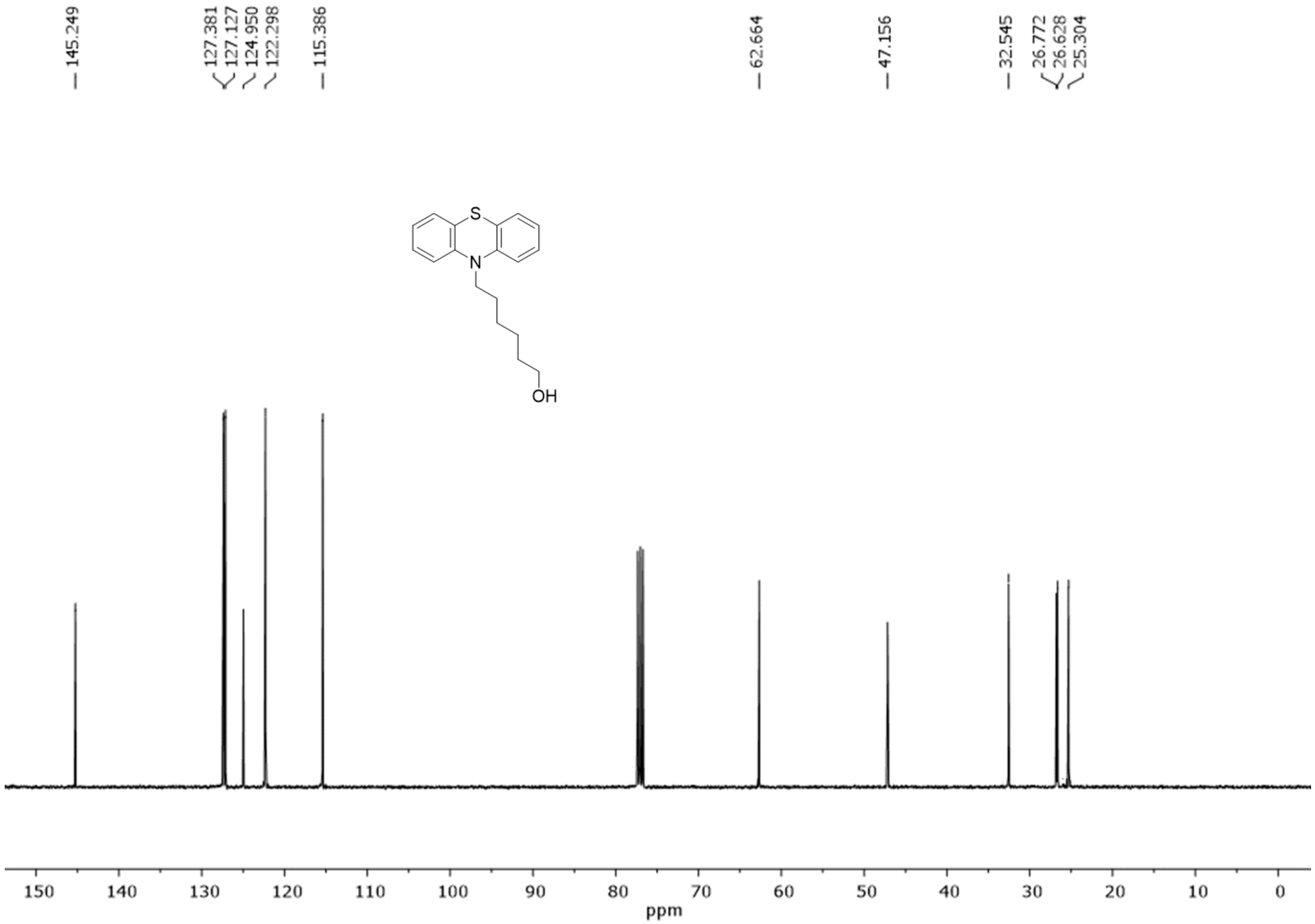

^{13}C NMR ($CDCl_3$, 100 MHz) of 6-(10H-phenothiazin-10-yl)hexan-1-ol

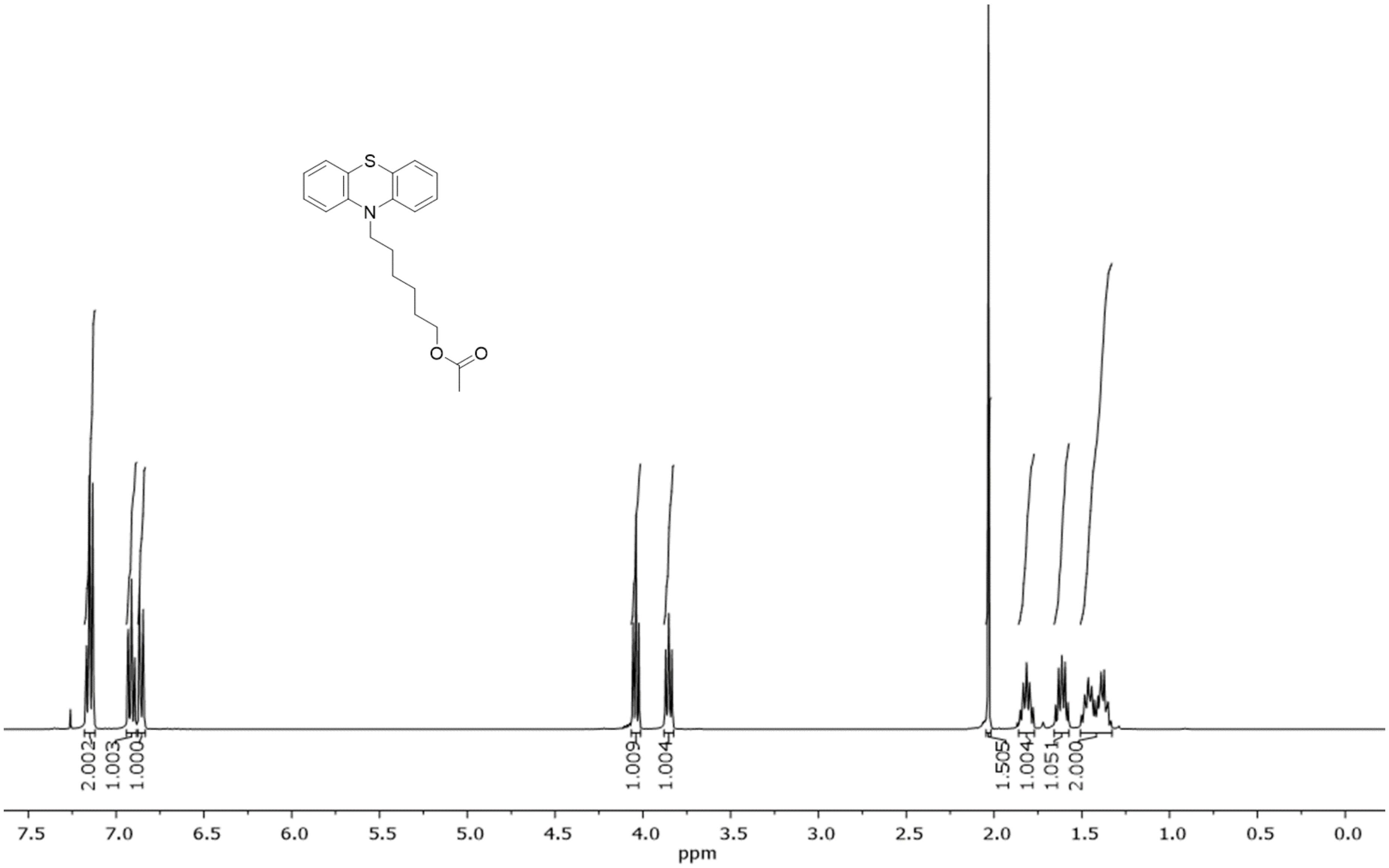

^{1}H NMR ($CDCl_3$, 400 MHz) of 6-(10H-phenothiazin-10-yl)hexyl acetate

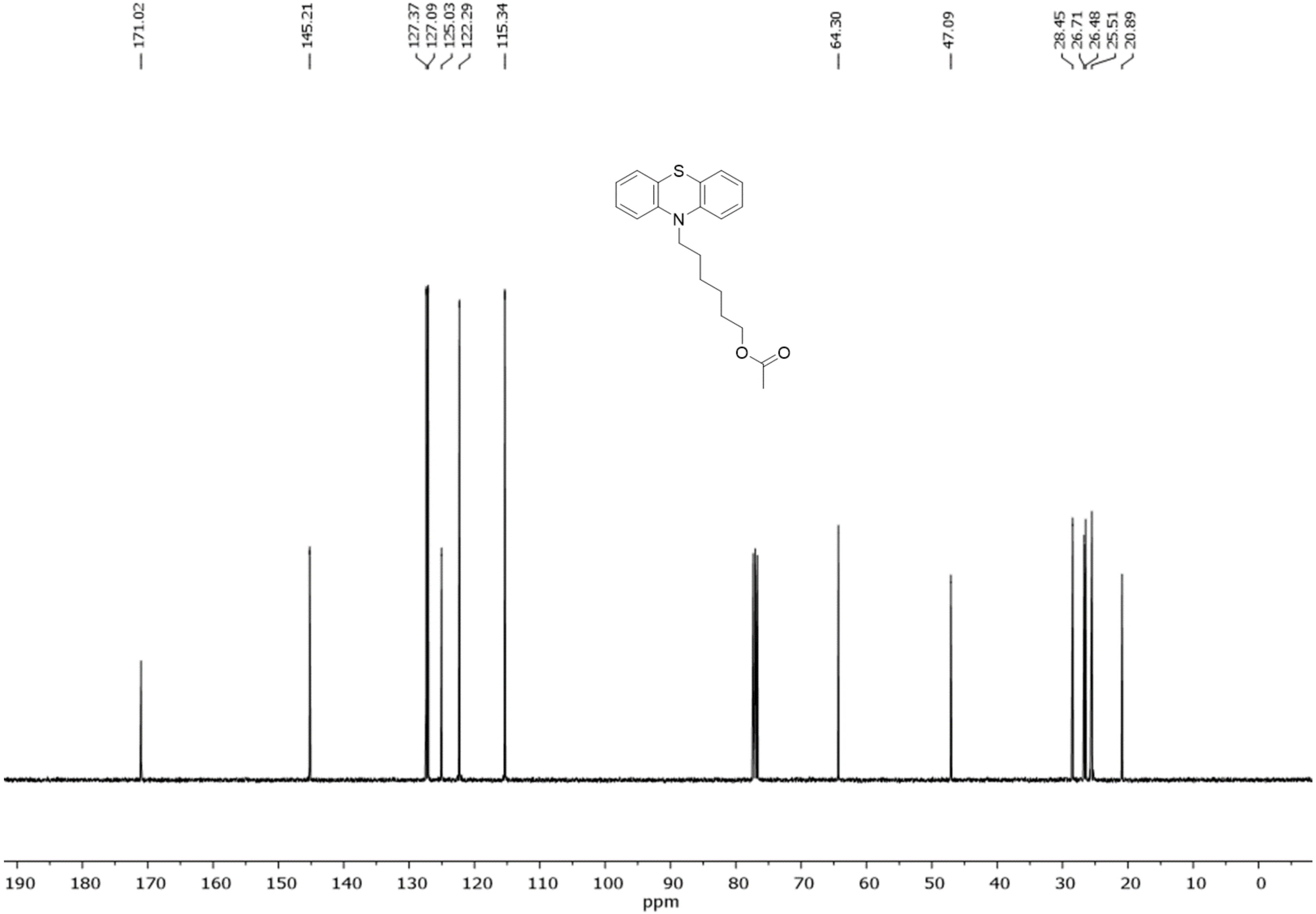

^{13}C NMR ($CDCl_3$, 100 MHz) of 6-(10H-phenothiazin-10-yl)hexyl acetate

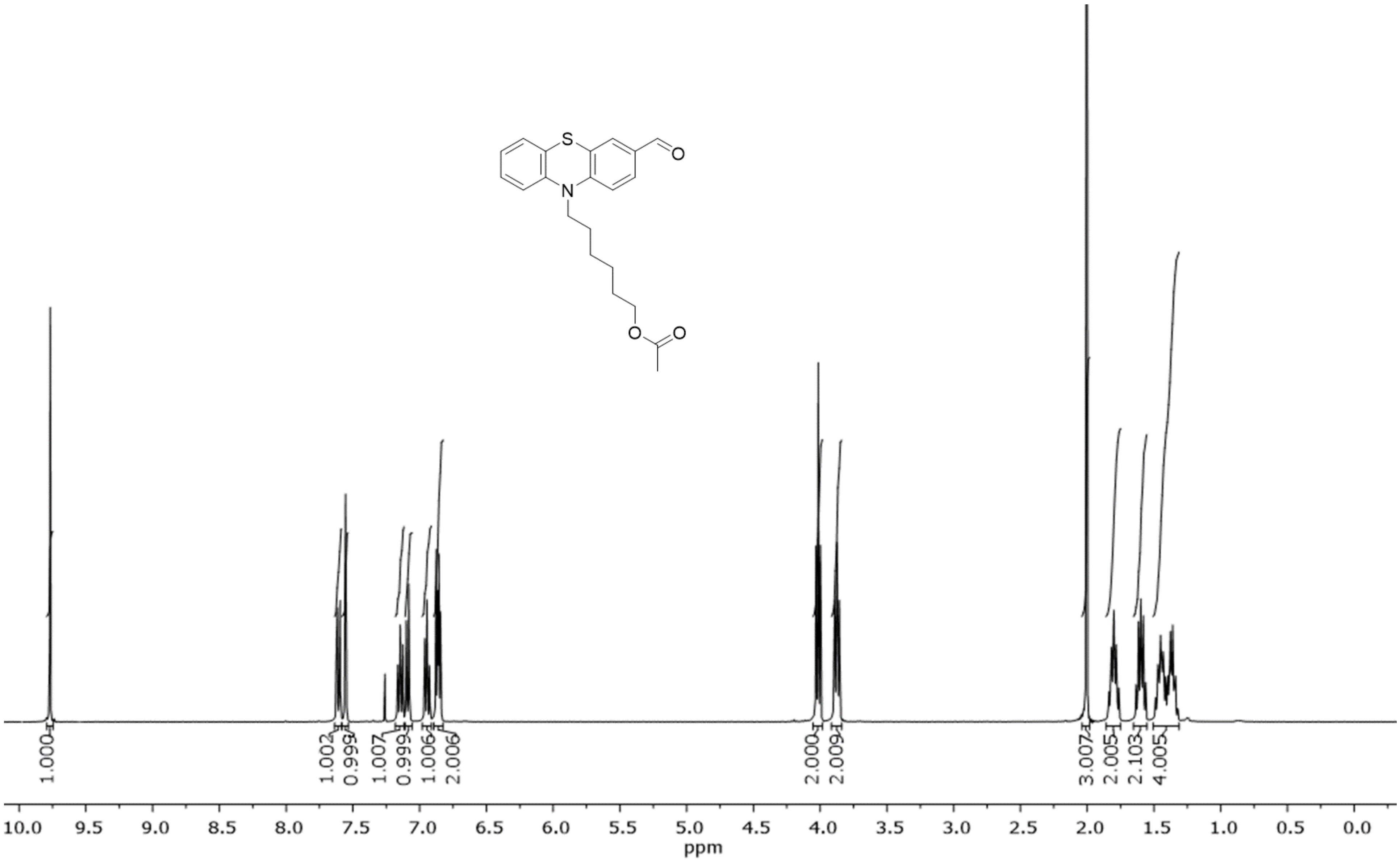

^{1}H NMR ($CDCl_3$, 400 MHz) of 6-(3-formyl-10H-phenothiazin-10-yl)hexyl acetate (**1e**)

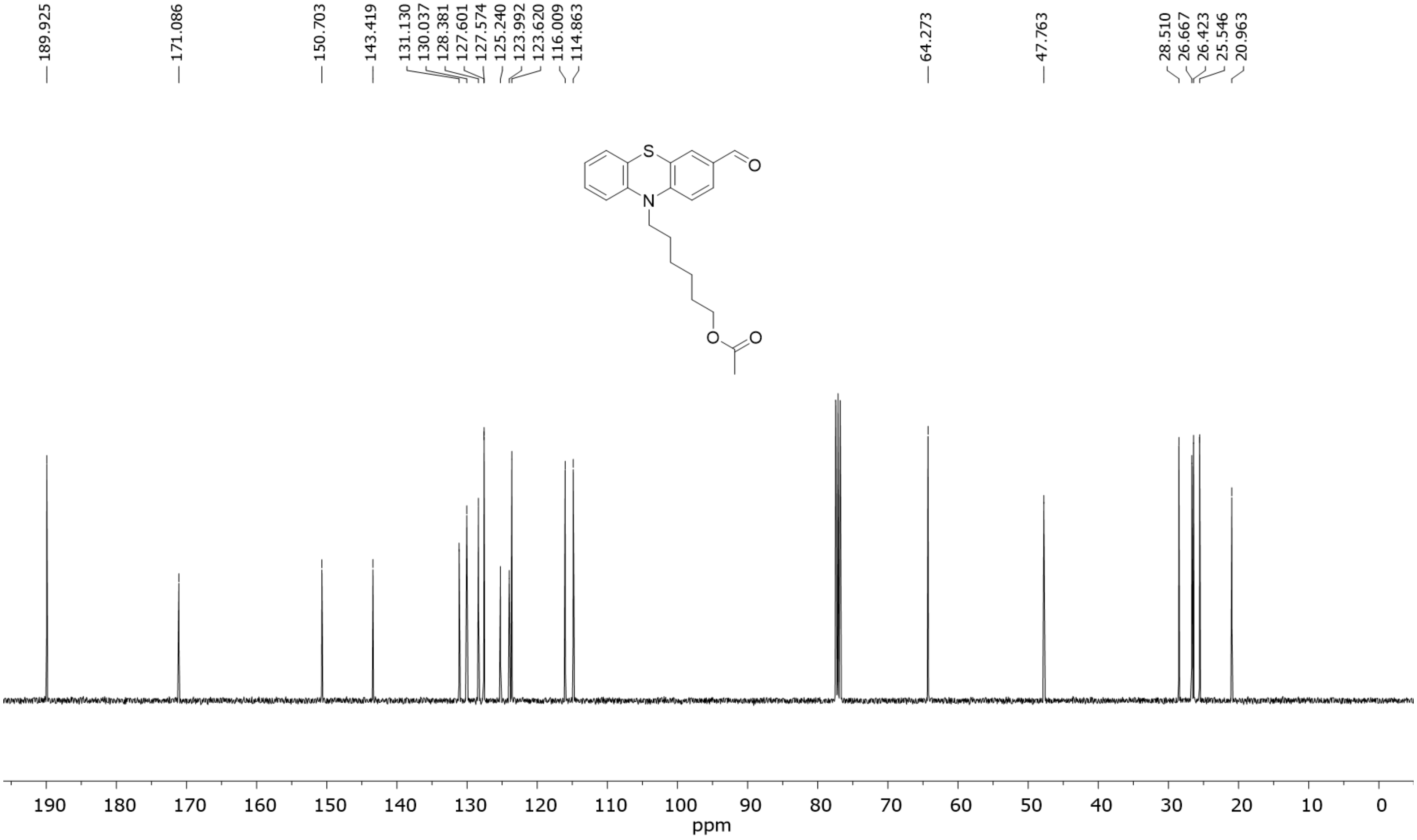

^{13}C NMR ($CDCl_3$, 100 MHz) of 6-(3-formyl-10H-phenothiazin-10-yl)hexyl acetate (**1e**)

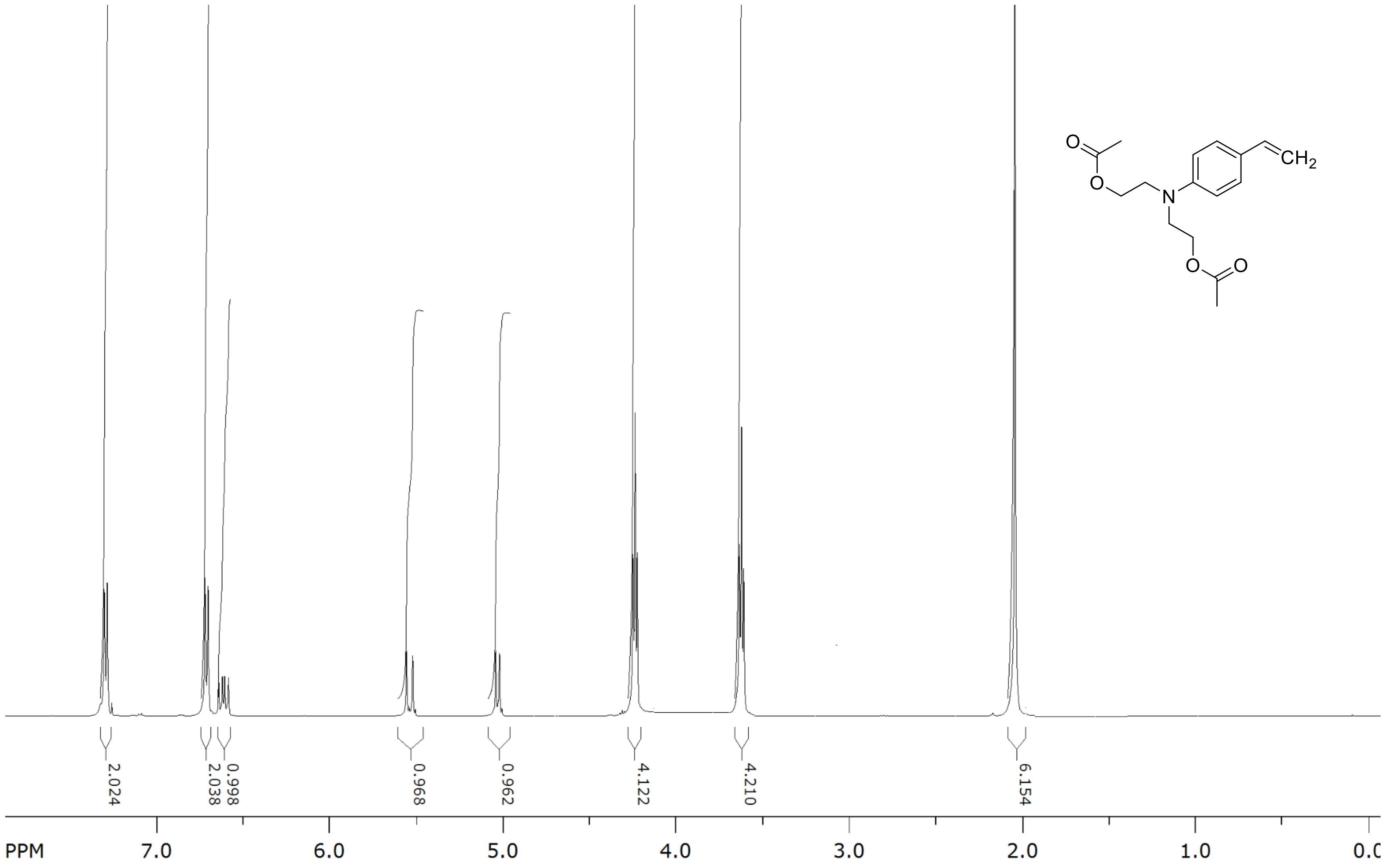

^{1}H NMR ($CDCl_3$, 400 MHz) of ((4-vinylphenyl)azanediyl)bis(ethane-2,1-diyl) diacetate (**2b**)

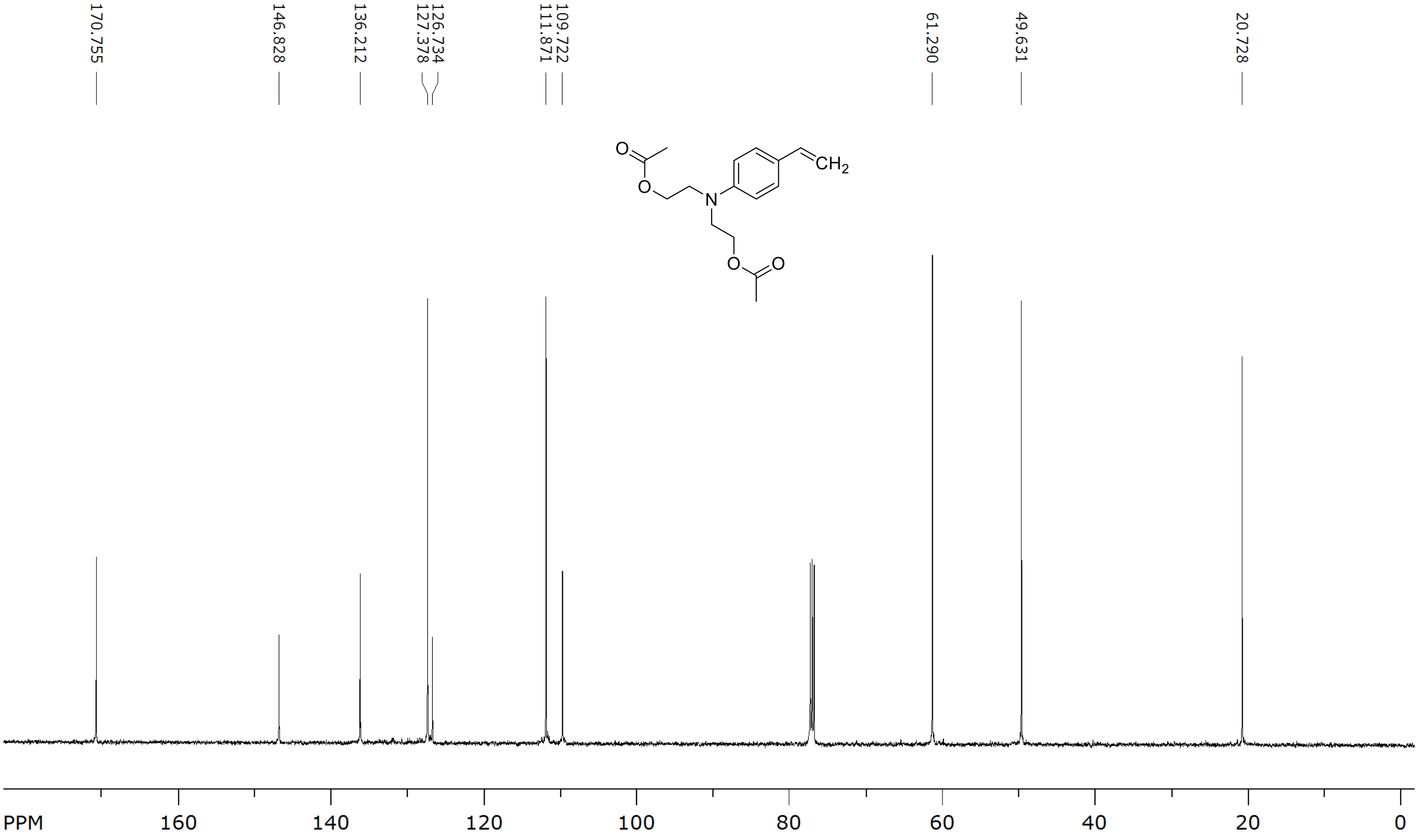

^{13}C NMR ($CDCl_3$, 100 MHz) of ((4-vinylphenyl)azanediyl)bis(ethane-2,1-diyl) diacetate (**2b**)

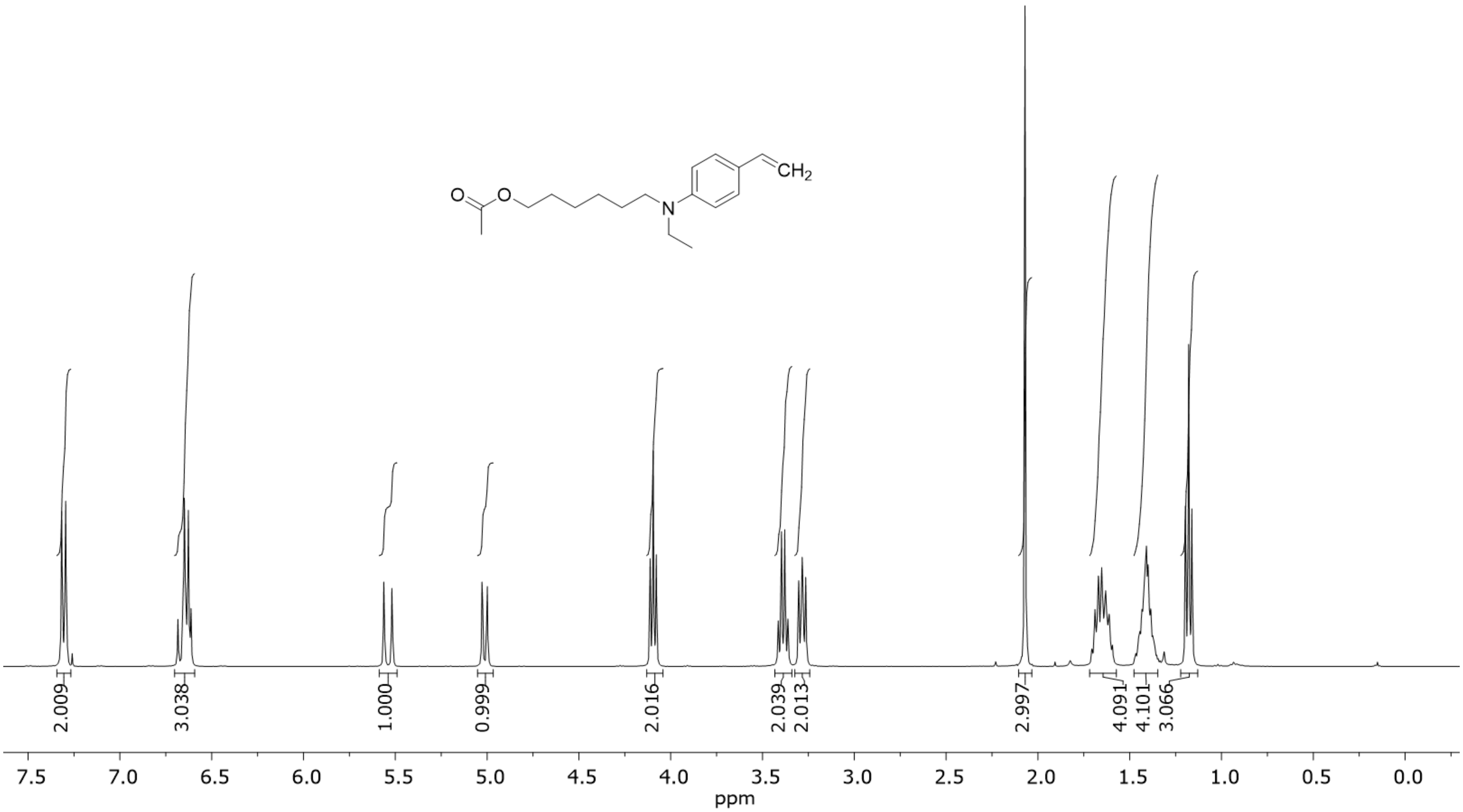

^{1}H NMR ($CDCl_3$, 400 MHz) of 6-(ethyl(4-vinylphenyl)amino)hexyl acetate (**2c**)

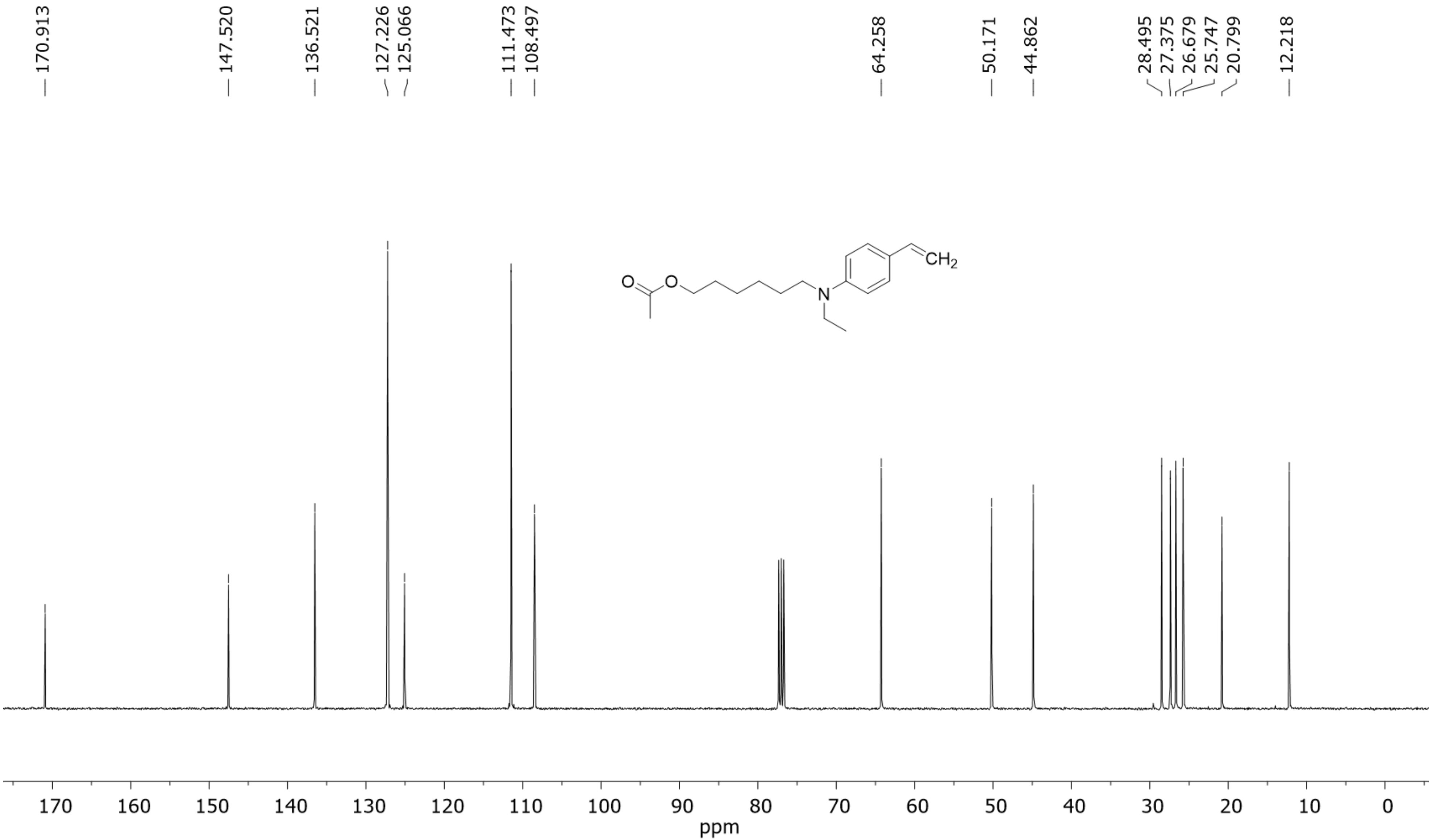

^{13}C NMR ($CDCl_3$, 100 MHz) of 6-(ethyl(4-vinylphenyl)amino)hexyl acetate (**2c**)

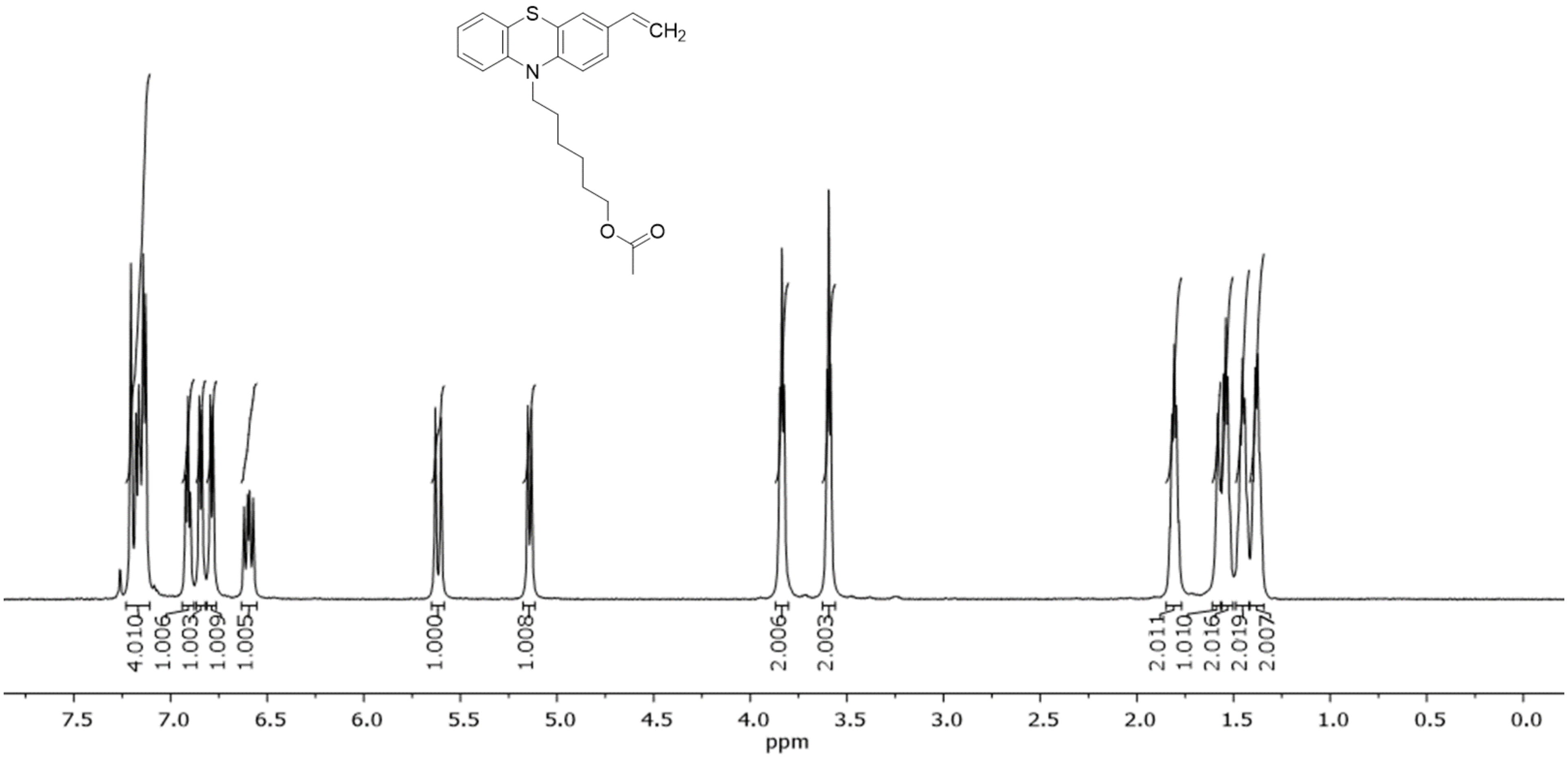

^{1}H NMR ($CDCl_3$, 600 MHz) of 6-(3-vinyl-10H-phenothiazin-10-yl)hexyl acetate (**2e'**)

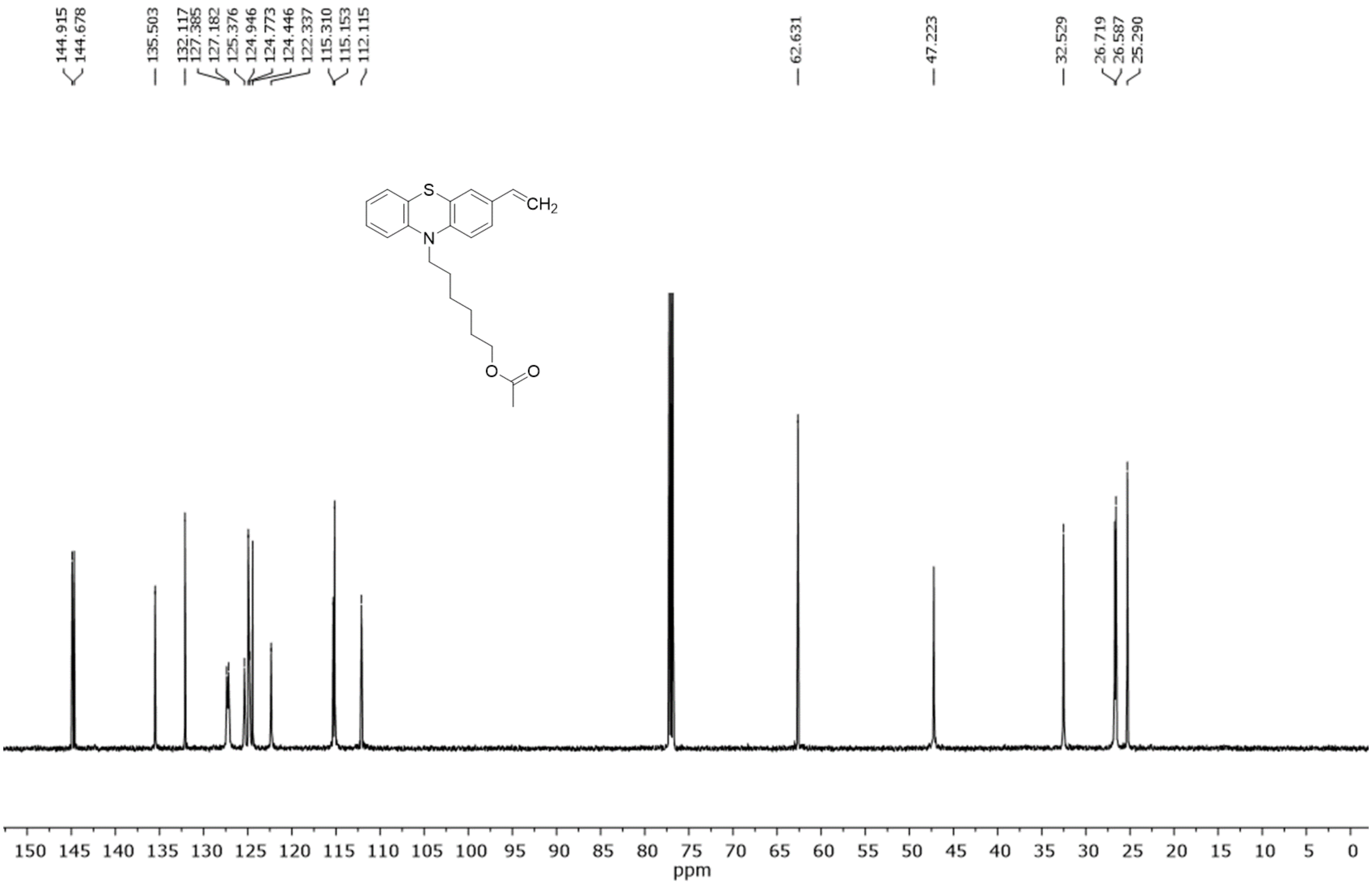

^{13}C NMR ($CDCl_3$, 150 MHz) of 6-(3-vinyl-10H-phenothiazin-10-yl)hexyl acetate (**2e'**)

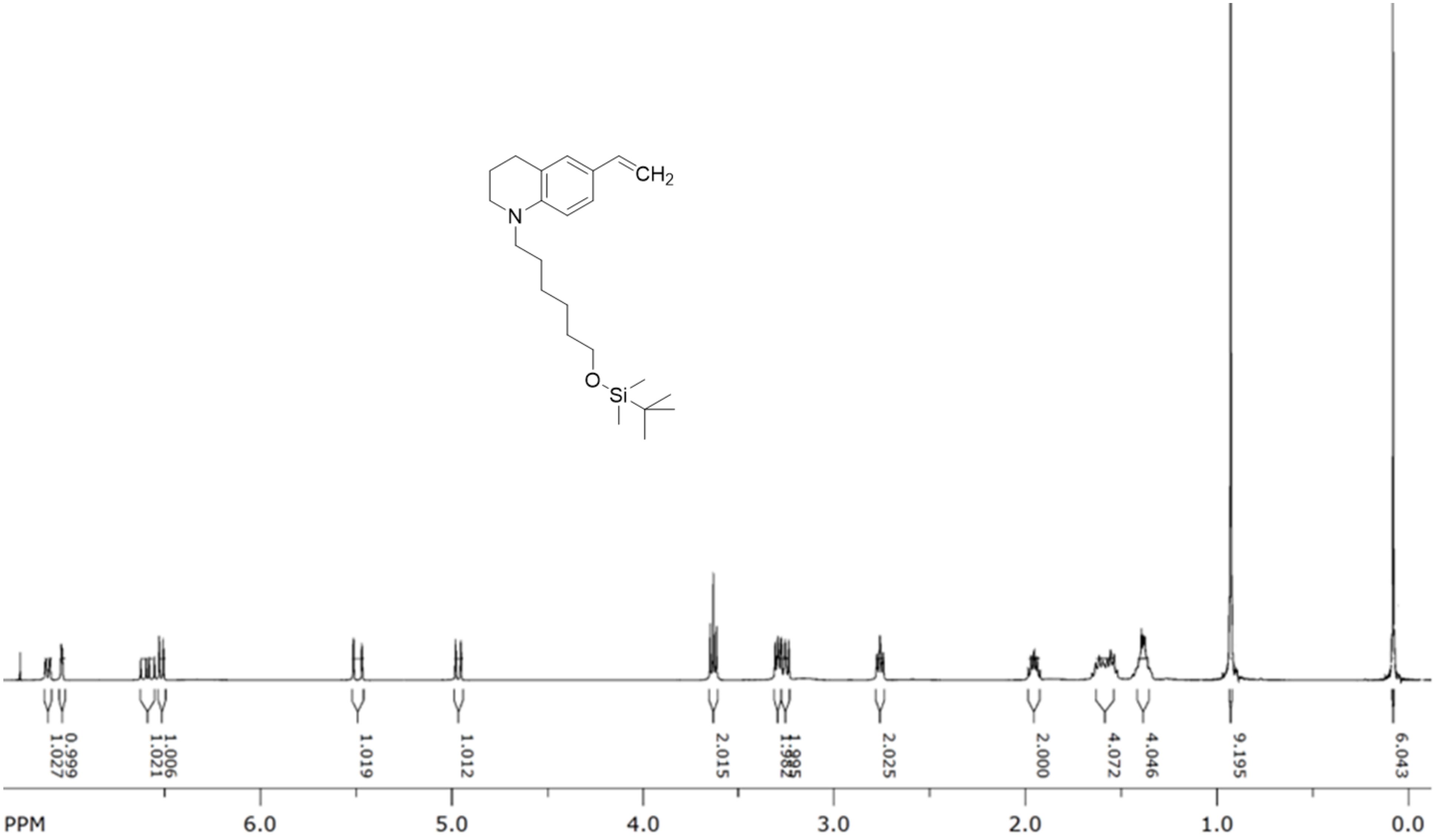

^{1}H NMR ($CDCl_3$, 500 MHz) of 1-(6-((*tert*-butyldimethylsilyl)oxy)hexyl)-6-vinyl-1,2,3,4-tetrahydroquinoline (**2f'**)

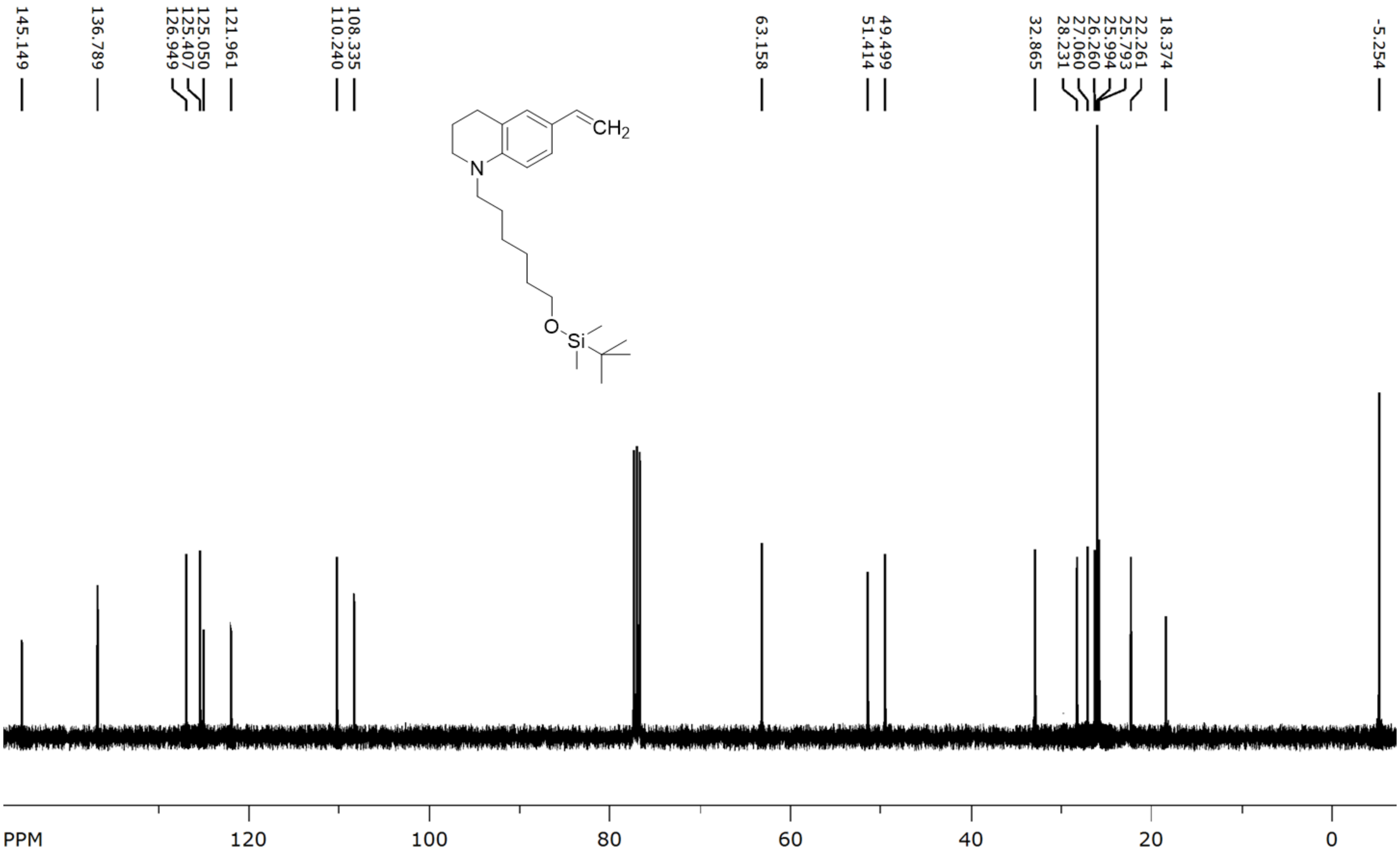

^{13}C NMR ($CDCl_3$, 125 MHz) of 1-(6-((tert-butyldimethylsilyl)oxy)hexyl)-6-vinyl-1,2,3,4-tetrahydroquinoline (**2f'**)

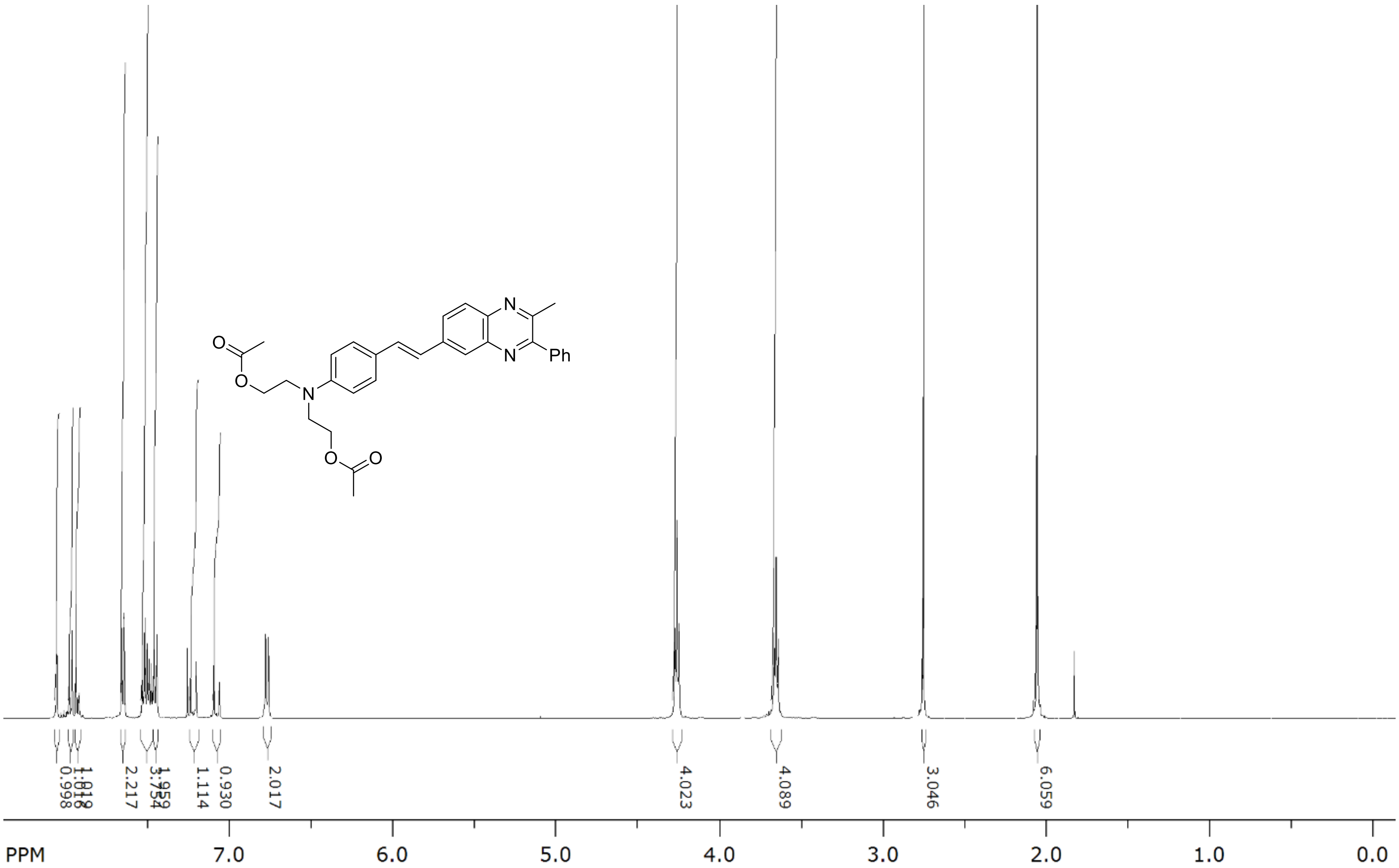

^{1}H NMR ($CDCl_3$, 500 MHz) of (*E*)-((4-(2-(2-methyl-3-phenylquinoxalin-6-yl)vinyl)phenyl)azanediyl)bis(ethane-2,1-diyl) diacetate (**4b**).

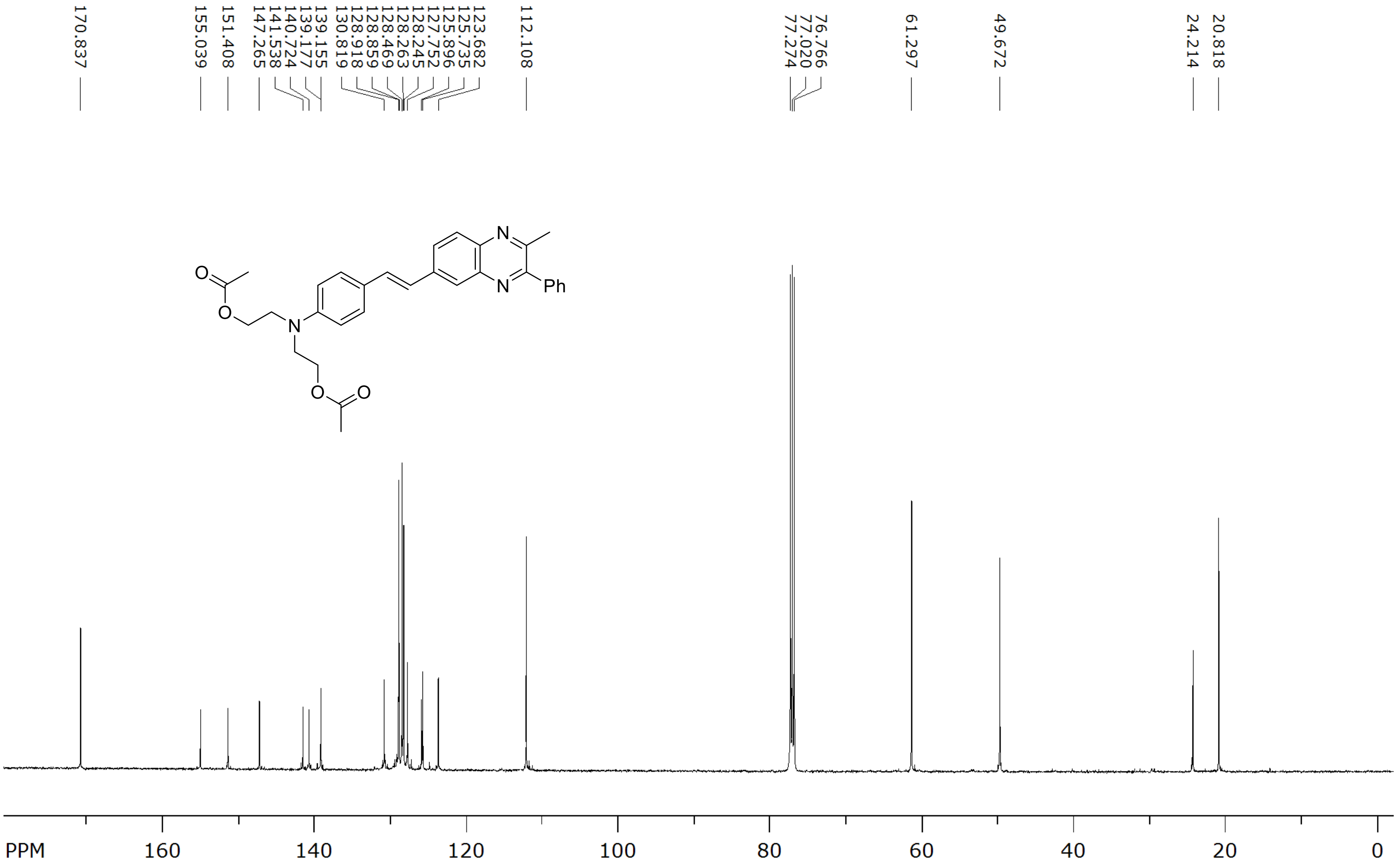

^{13}C NMR ($CDCl_3$, 125 MHz) of (*E*)-((4-(2-(2-methyl-3-phenylquinoxalin-6-yl)vinyl)phenyl)azanediyl)bis(ethane-2,1-diyl) diacetate (**4b**)

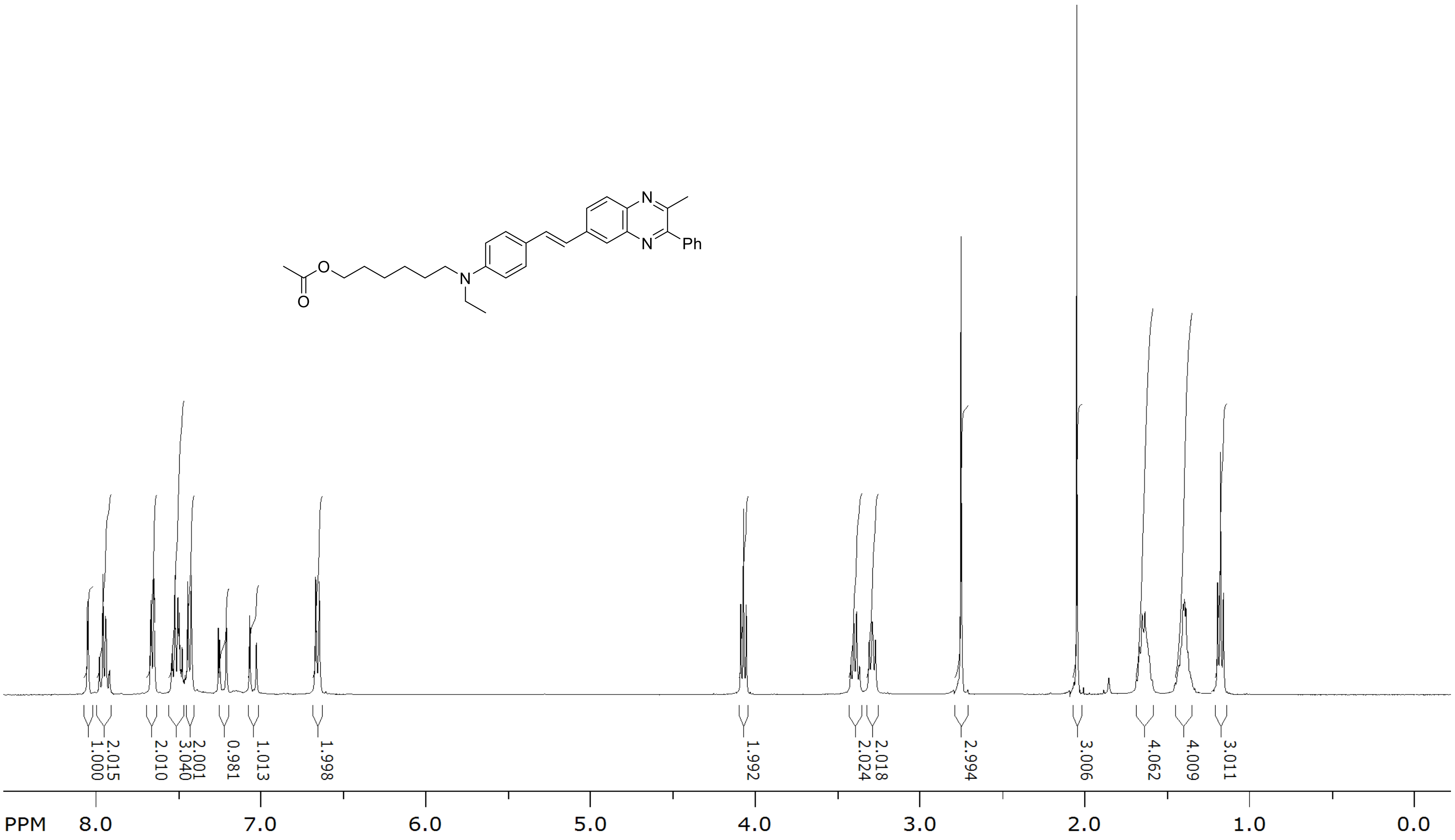

^{1}H NMR ($CDCl_3$, 400 MHz) of (E)-6-(ethyl(4-(2-(2-methyl-3-phenylquinoxalin-6-yl)vinyl)phenyl)amino)hexyl acetate (**4c**).

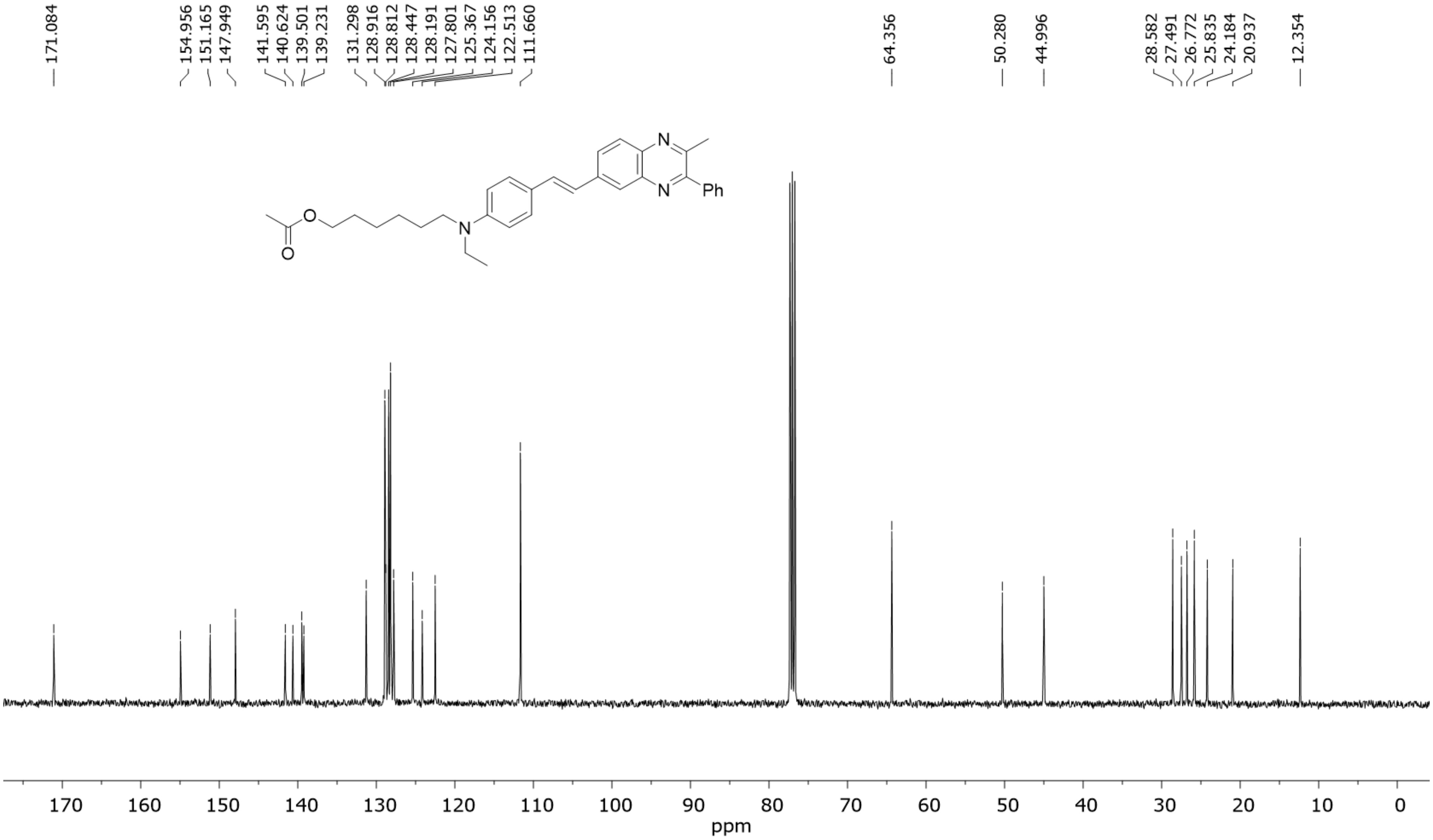

^{13}C NMR ($CDCl_3$, 100 MHz) of (E)-6-(ethyl(4-(2-(2-methyl-3-phenylquinoxalin-6-yl)vinyl)phenyl)amino)hexyl acetate (**4c**)

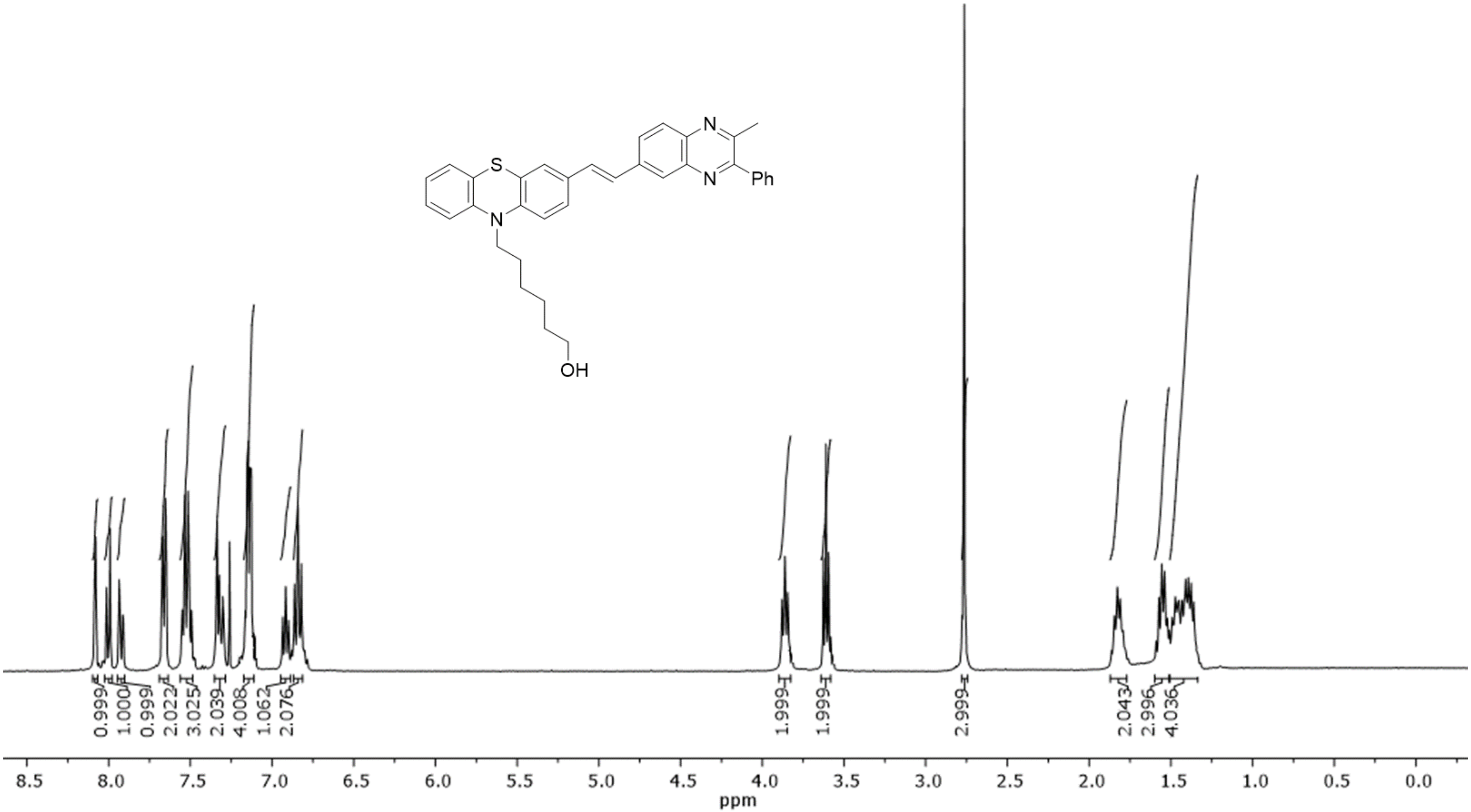

^{1}H NMR ($CDCl_3$, 400 MHz) of (E)-6-(3-(2-(2-methyl-3-phenylquinoxalin-6-yl)vinyl)-10H-phenothiazin-10-yl)hexan-1-ol (**5e**)

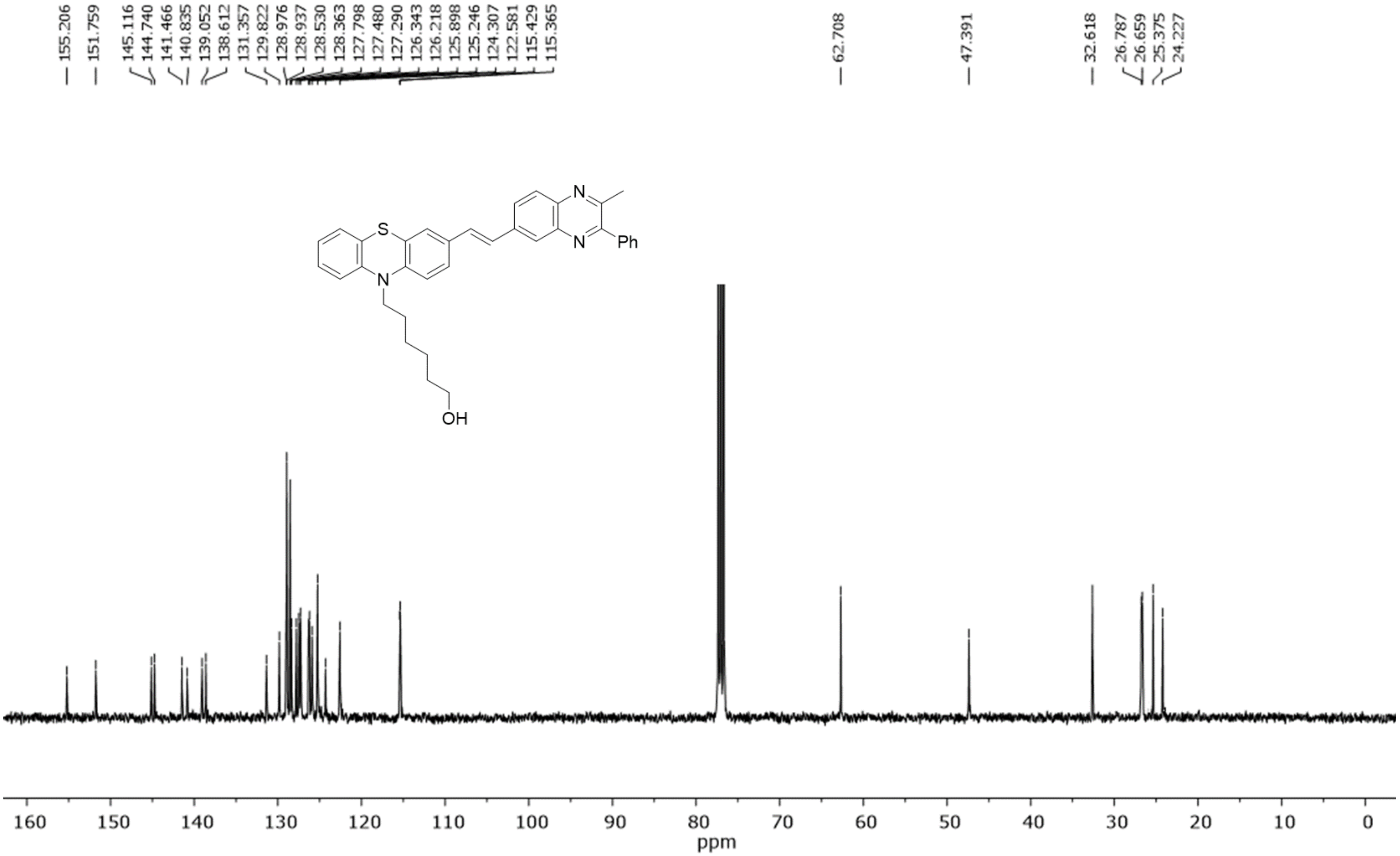

^{13}C NMR ($CDCl_3$, 100 MHz) of (E)-6-(3-(2-(2-methyl-3-phenylquinoxalin-6-yl)vinyl)-10H-phenothiazin-10-yl)hexan-1-ol (**5e**)

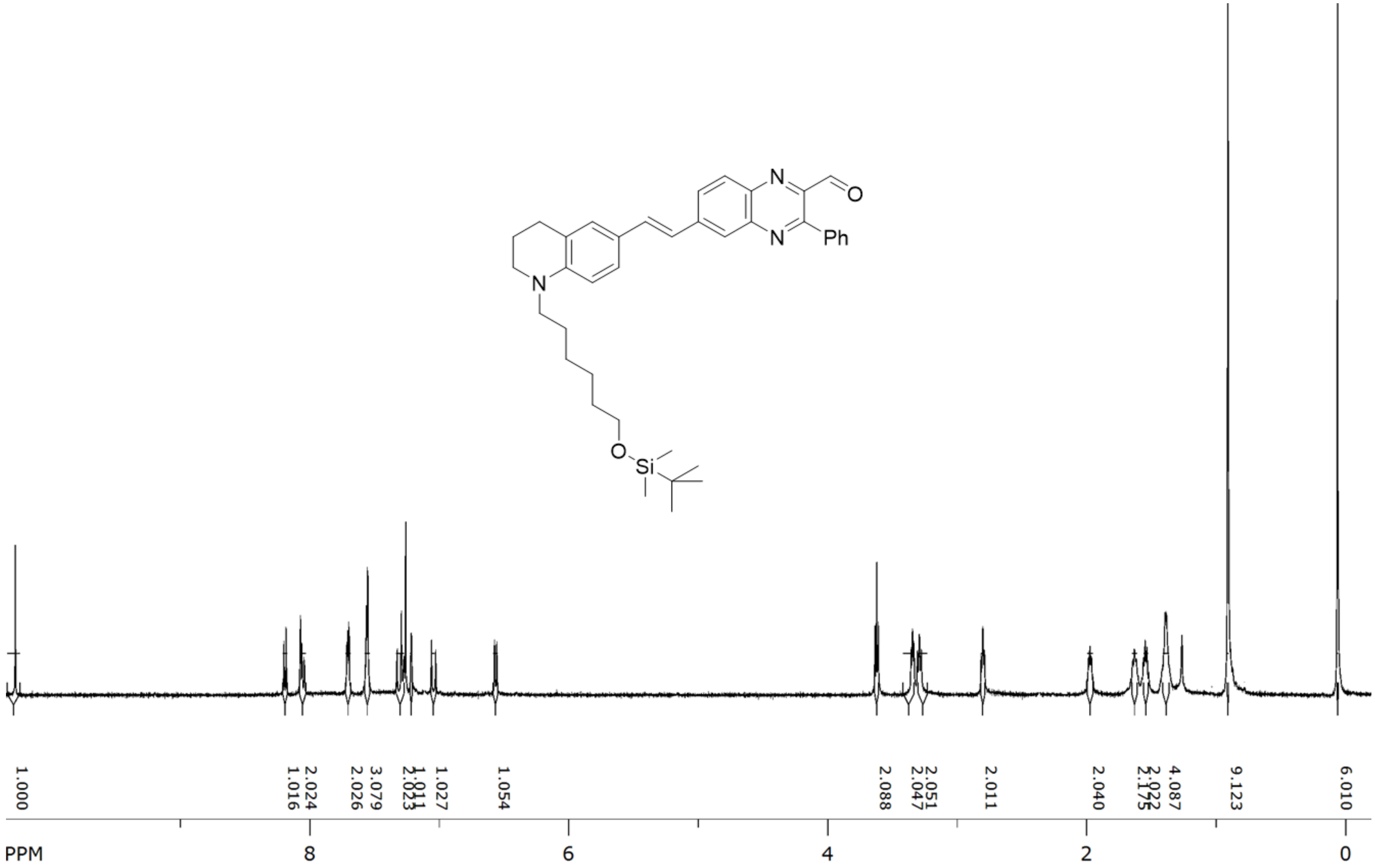

^{1}H NMR ($CDCl_3$, 500 MHz) of (*E*)-6-(2-(1-(6-((tert-butyldimethylsilyl)oxy)hexyl)-1,2,3,4-tetrahydroquinolin-6-yl)vinyl)-3-phenylquinoxaline-2-carbaldehyde (**7f**)

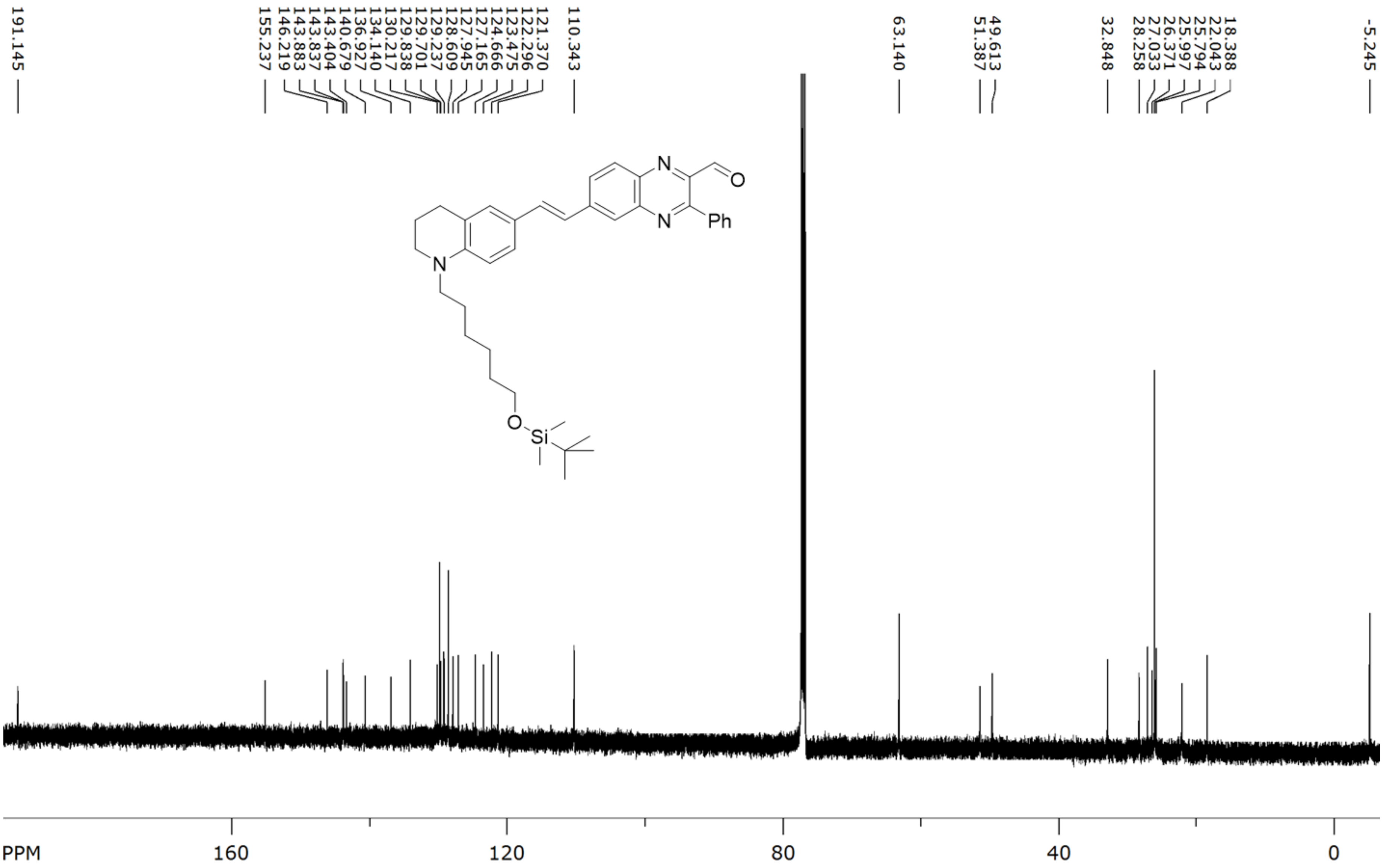

^{13}C NMR ($CDCl_3$, 125 MHz) of (E)-6-(2-(1-(6-((tert-butyldimethylsilyl)oxy)hexyl)-1,2,3,4-tetrahydroquinolin-6-yl)vinyl)-3-phenylquinoxaline-2-carbaldehyde (**7f**)

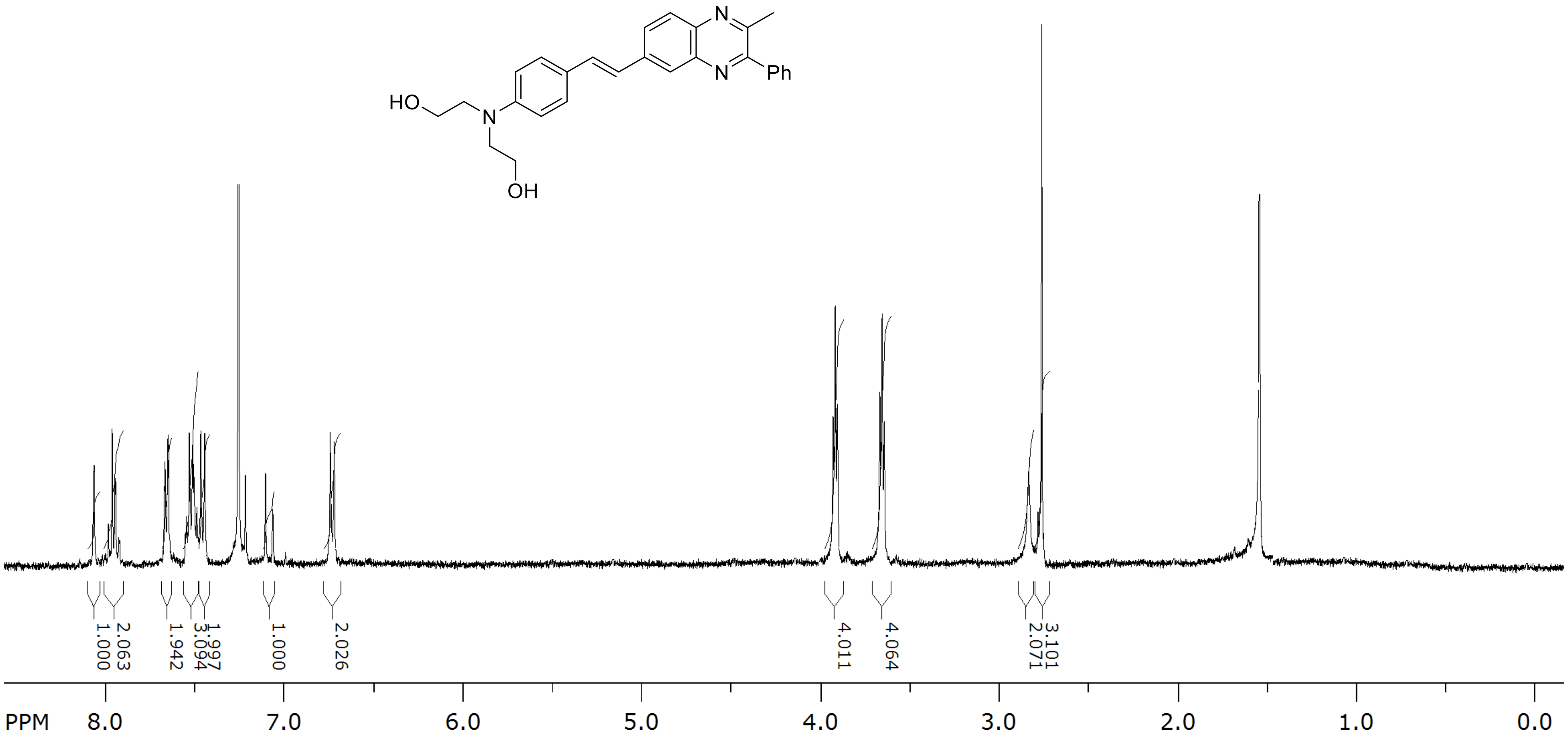

^{1}H NMR ($CDCl_3$, 500 MHz) of (*E*)-2,2'-((4-(2-(2-methyl-3-phenylquinoxalin-6-yl)vinyl)phenyl)azanediyl)bis(ethan-1-ol) (**5b**)

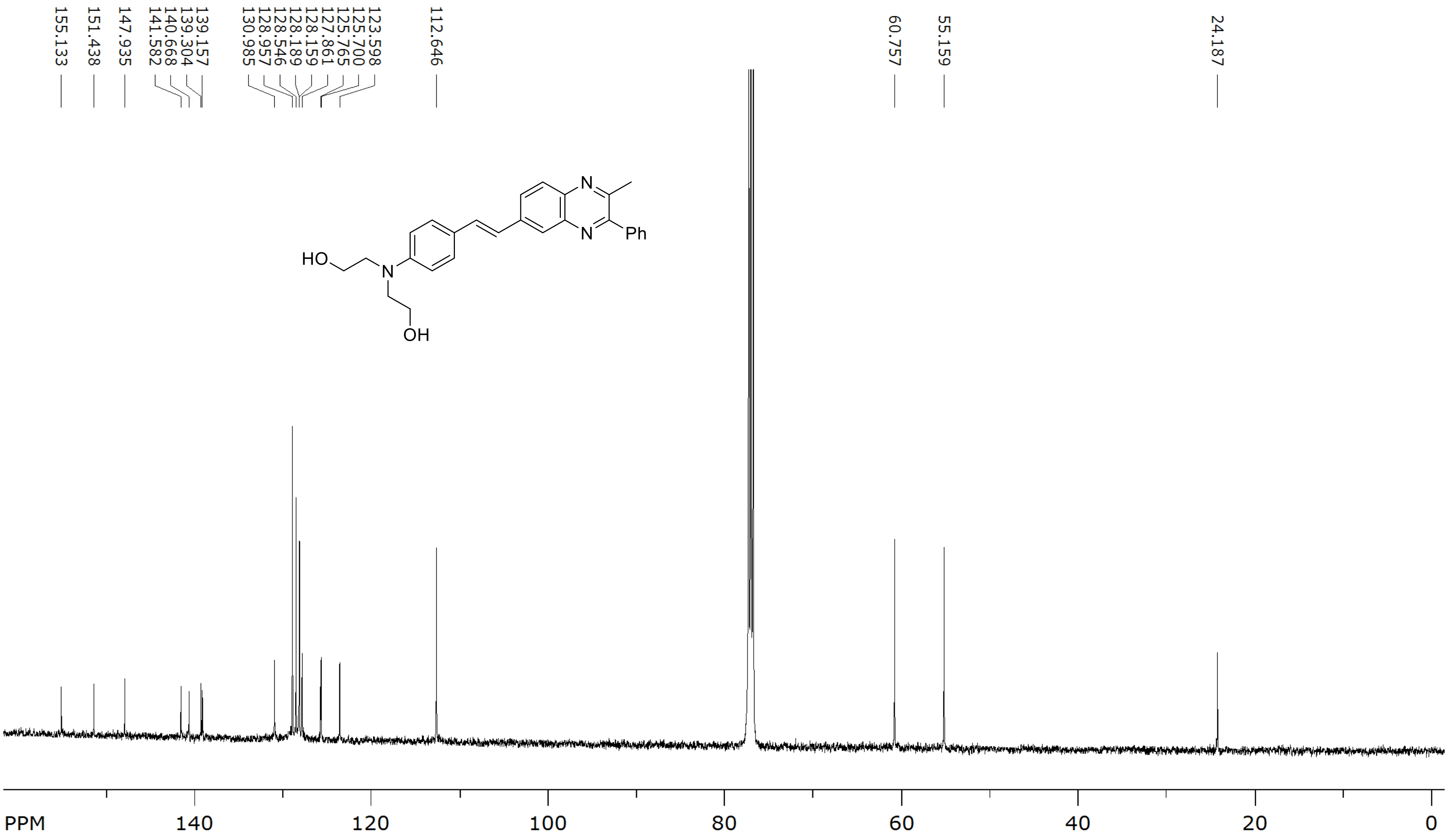

^{13}C NMR ($CDCl_3$, 125 MHz) of (*E*)-2,2'-((4-(2-(2-methyl-3-phenylquinoxalin-6-yl)vinyl)phenyl)azanediyl)bis(ethan-1-ol) (**5b**)

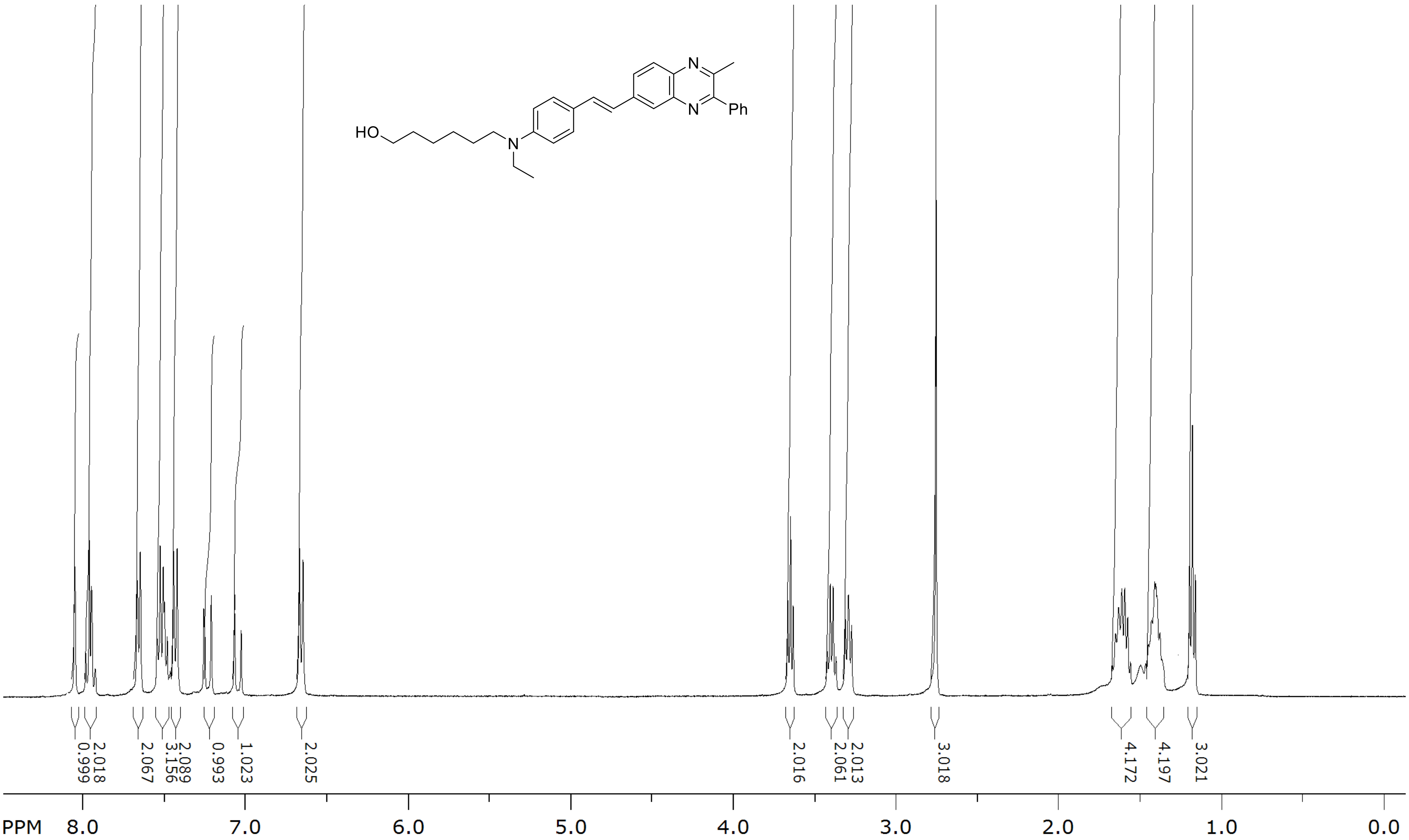

^{1}H NMR ($CDCl_3$, 400 MHz) of (*E*)-6-(ethyl(4-(2-(2-methyl-3-phenylquinoxalin-6-yl)vinyl)phenyl)amino)hexan-1-ol (**5c**)

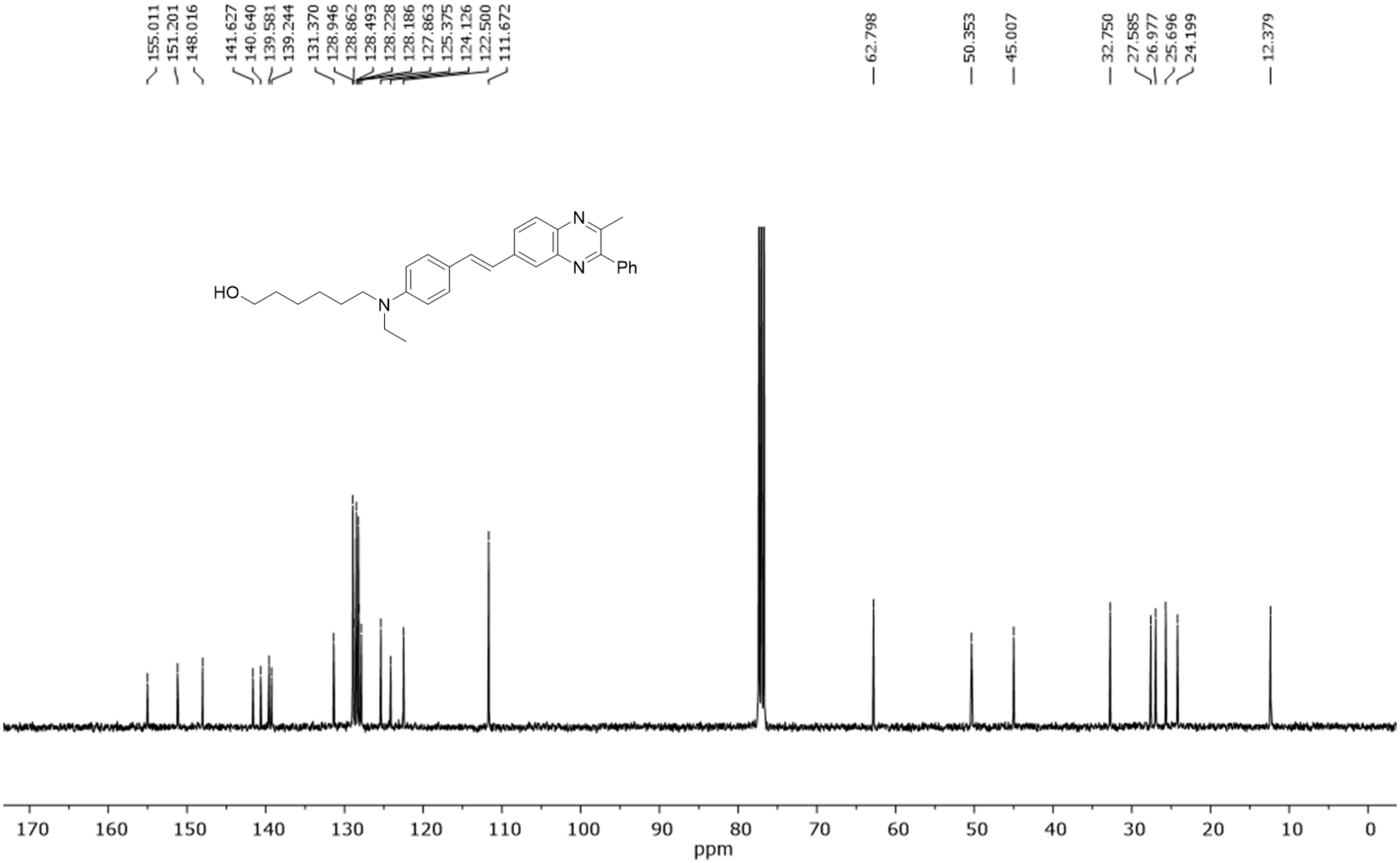

^{13}C NMR ($CDCl_3$, 100 MHz) of (E)-6-(ethyl(4-(2-(2-methyl-3-phenylquinoxalin-6-yl)vinyl)phenyl)amino)hexan-1-ol (**5c**)

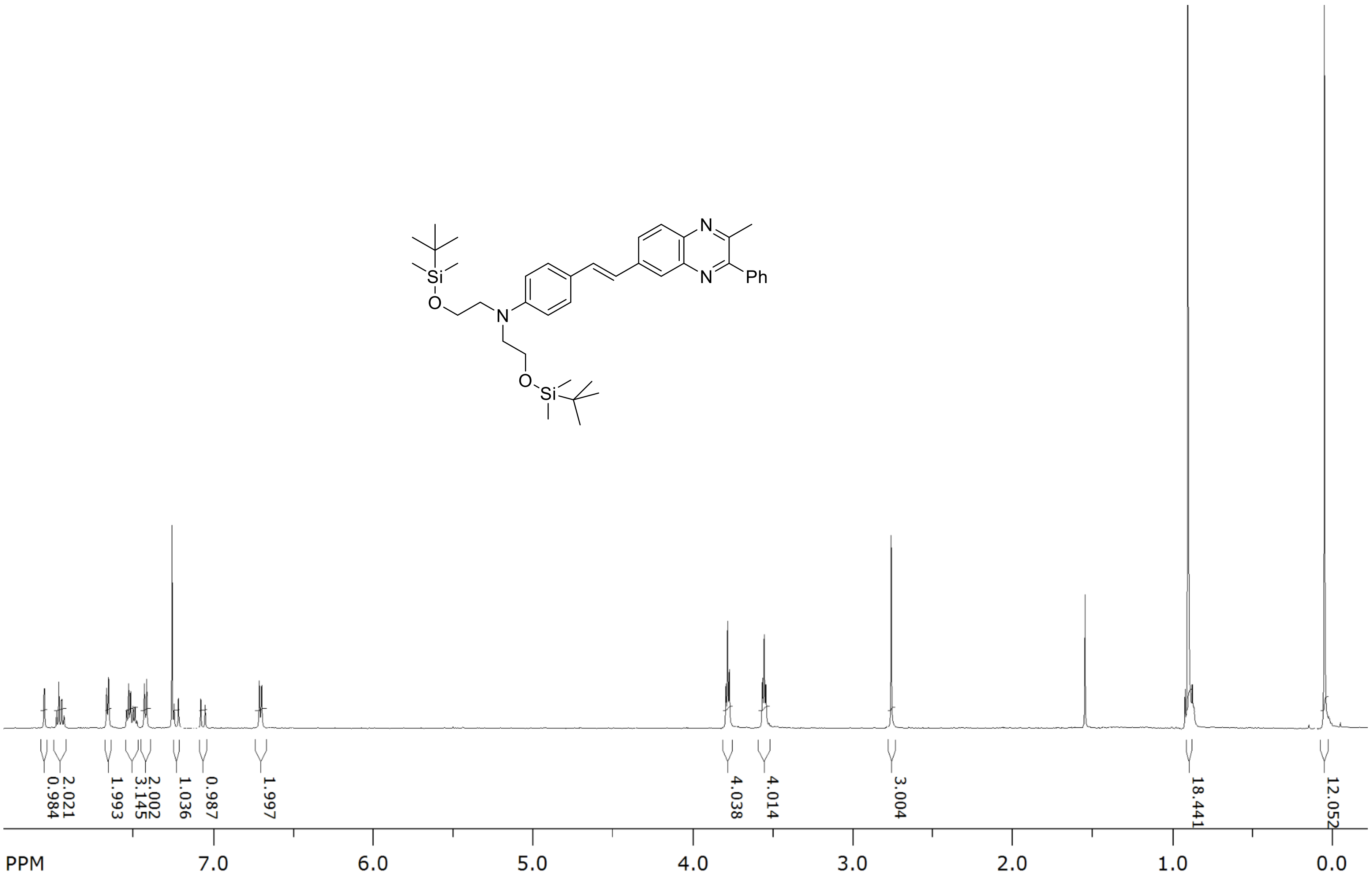

^{1}H NMR ($CDCl_3$, 400 MHz) of (*E*)-*N*,*N*-bis(2-((tert-butyldimethylsilyl)oxy)ethyl)-4-(2-(2-methyl-3-phenylquinoxalin-6-yl)vinyl)aniline (**6b**).

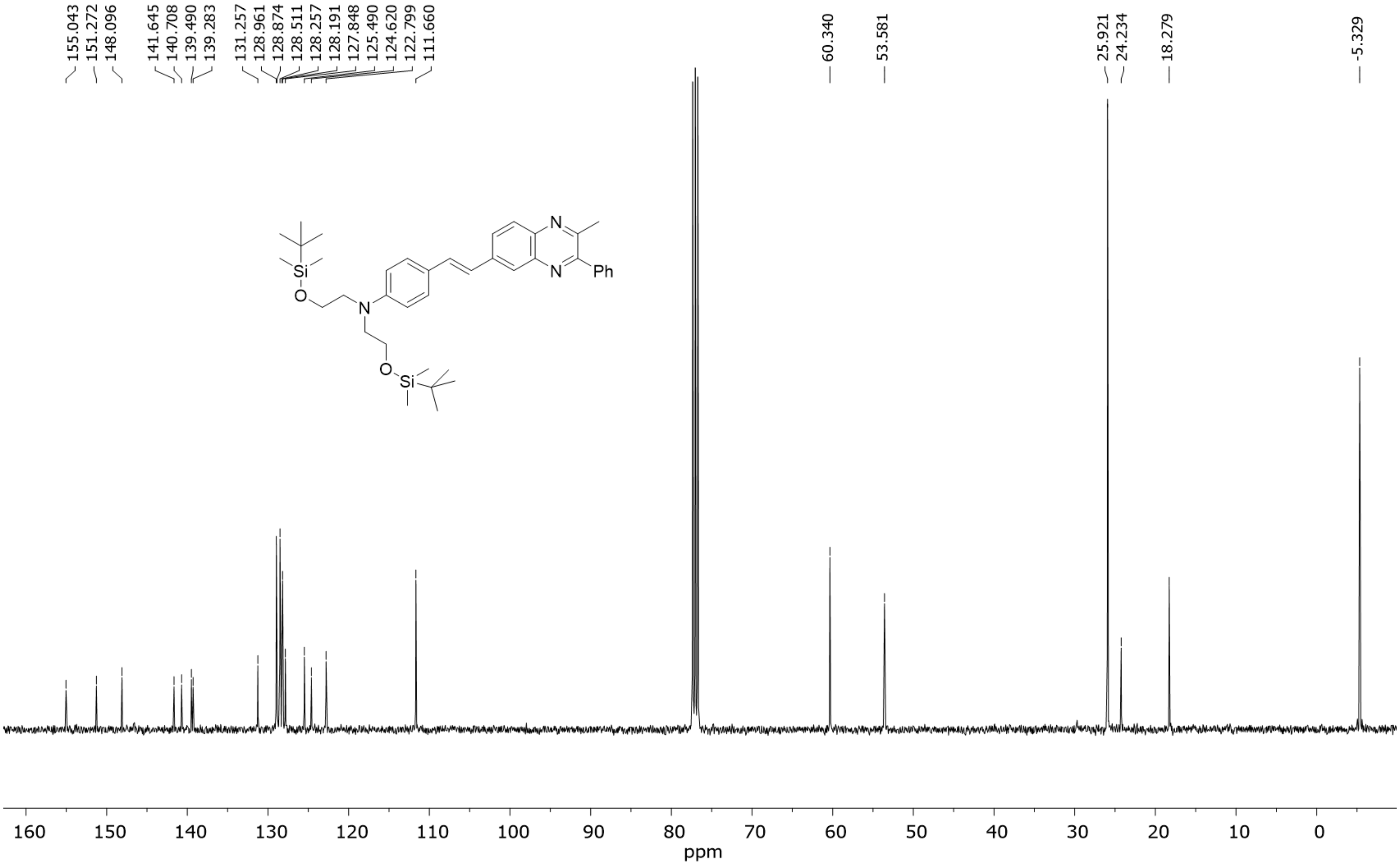

^{13}C NMR ($CDCl_3$, 100 MHz) of (*E*)-*N*,*N*-bis(2-((*tert*-butyldimethylsilyl)oxy)ethyl)-4-(2-(2-methyl-3-phenylquinoxalin-6-yl)vinyl)aniline (**6b**)

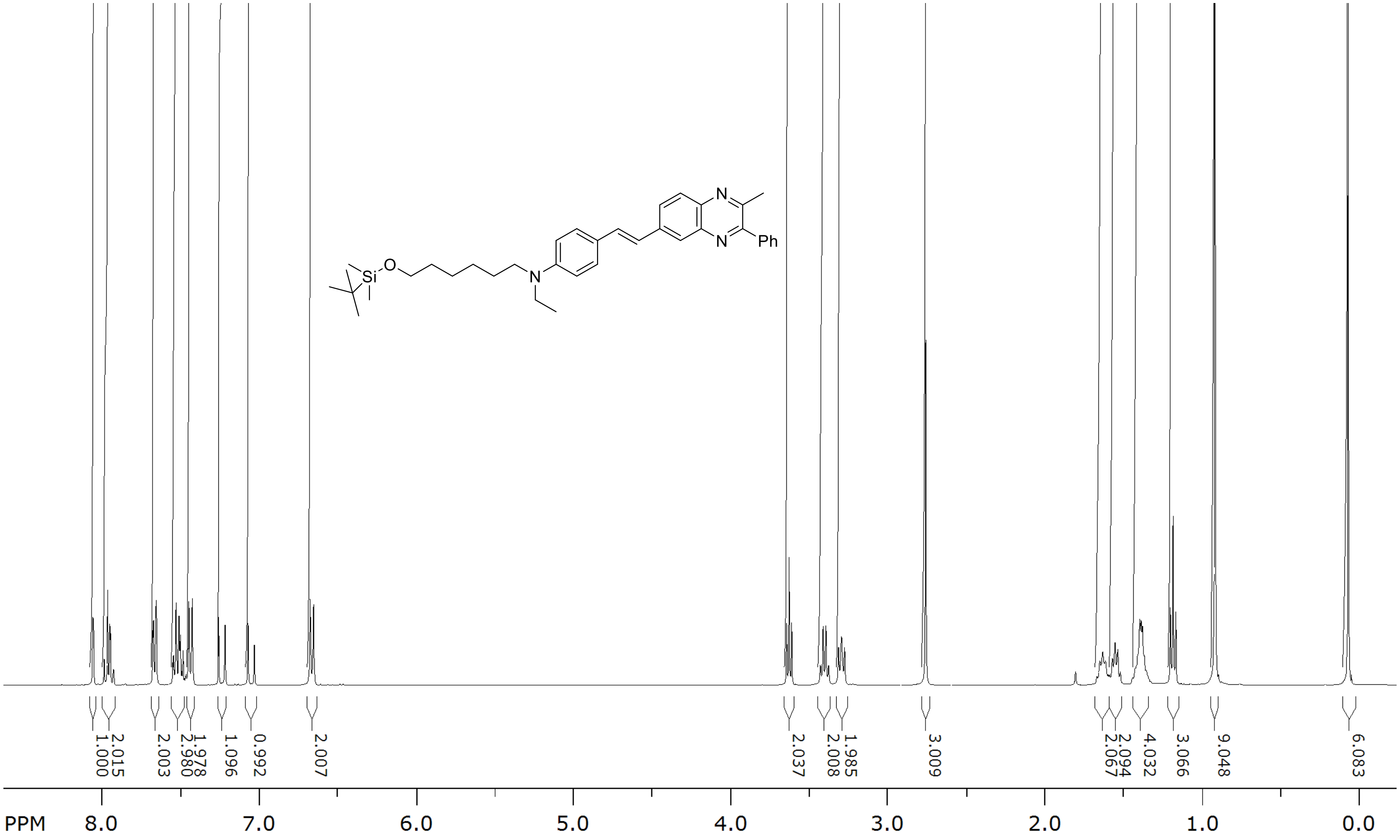

^{1}H NMR ($CDCl_3$, 400 MHz) of (*E*)-N-(6-((*tert*-butyldimethylsilyl)oxy)hexyl)-*N*-ethyl-4-(2-(2-methyl-3-phenylquinoxalin-6-yl)vinyl)aniline (**6c**)

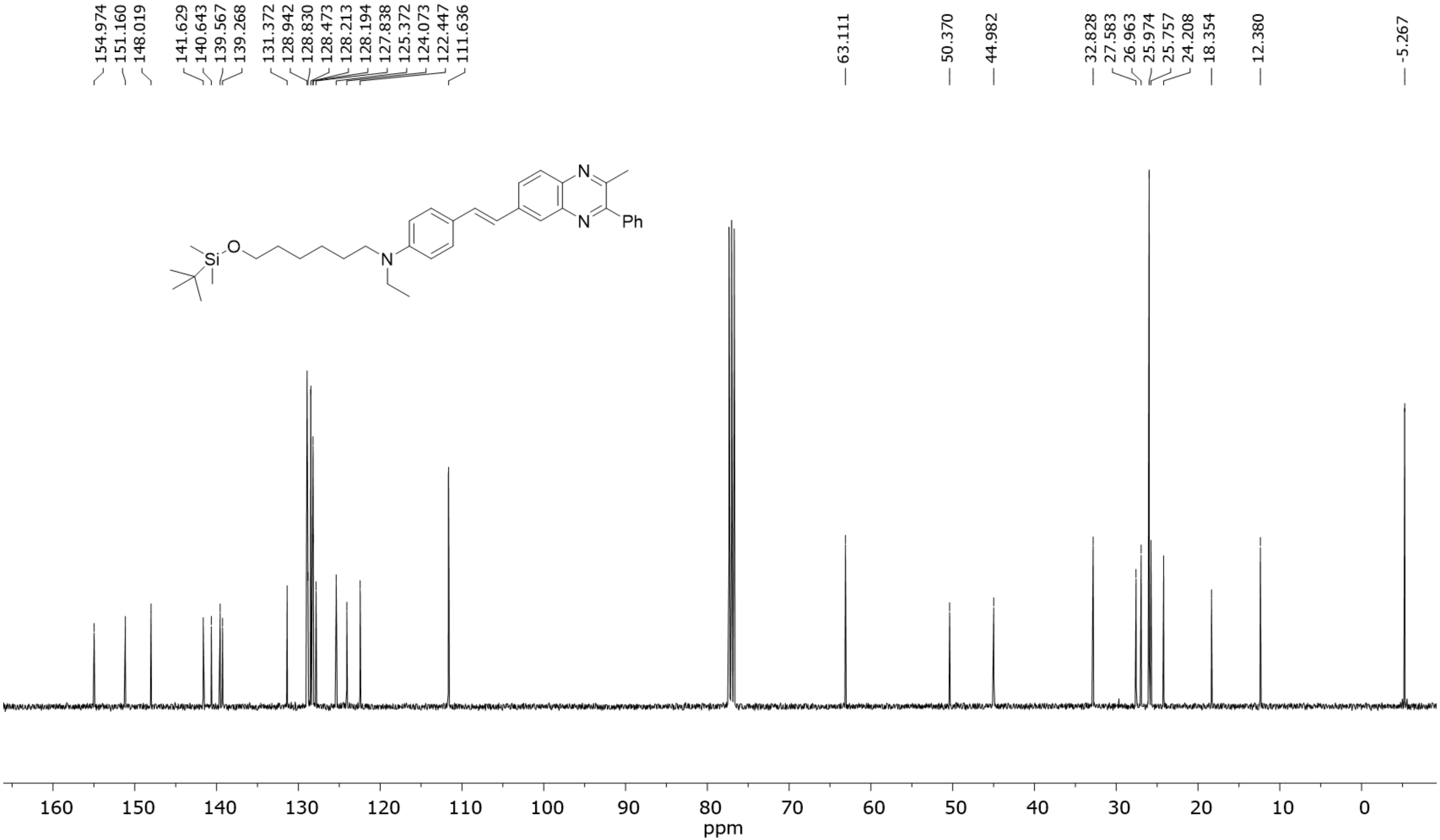

^{13}C NMR ($CDCl_3$, 100 MHz) of (E)-N-(6-((tert-butyldimethylsilyl)oxy)hexyl)-N-ethyl-4-(2-(2-methyl-3-phenylquinoxalin-6-yl)vinyl)aniline (**6c**)

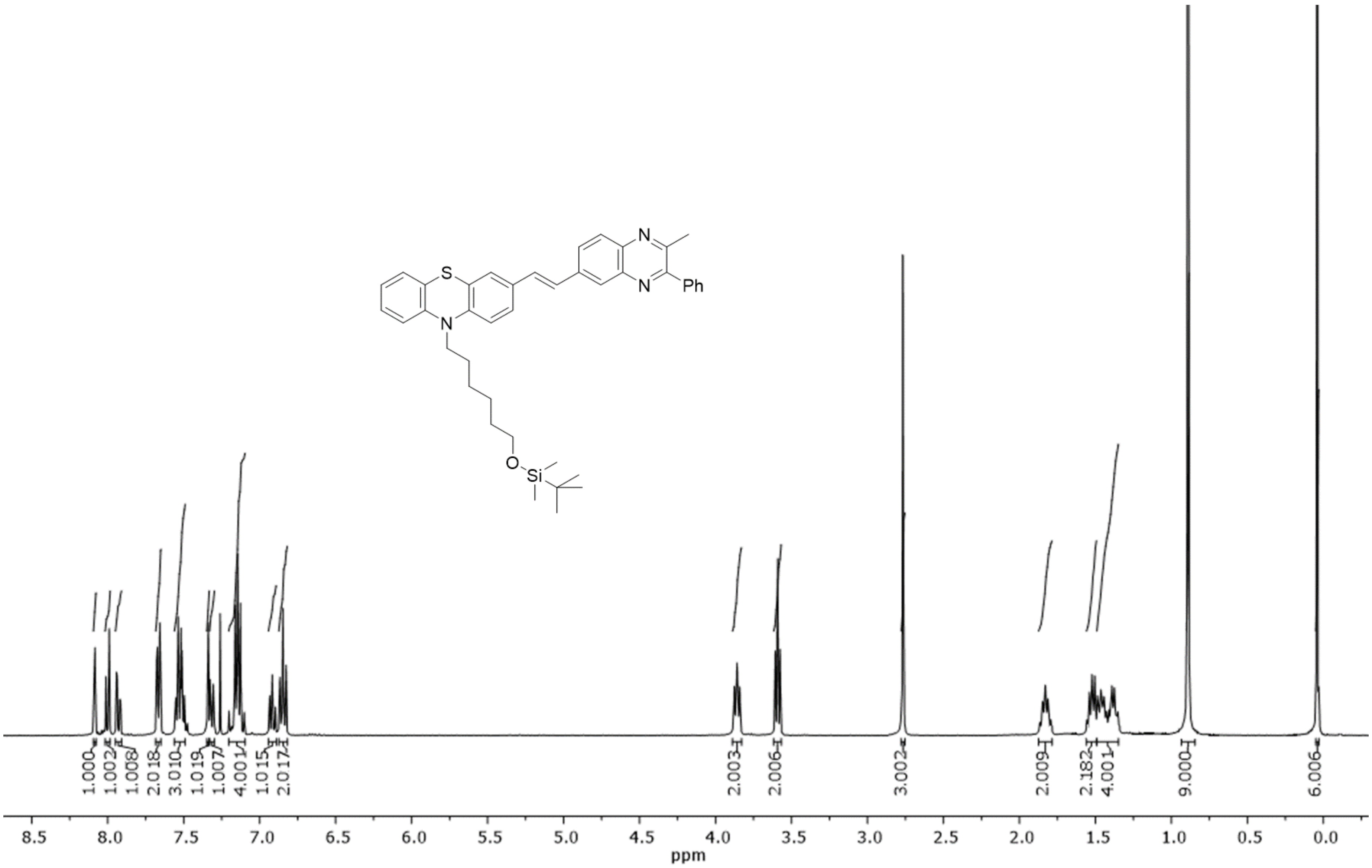

^{1}H NMR ($CDCl_3$, 400 MHz) of (E)-10-(6-((tert-butyldimethylsilyl)oxy)hexyl)-3-(2-(2-methyl-3-phenylquinoxalin-6-yl)vinyl)-10H-phenothiazine (**6e**)

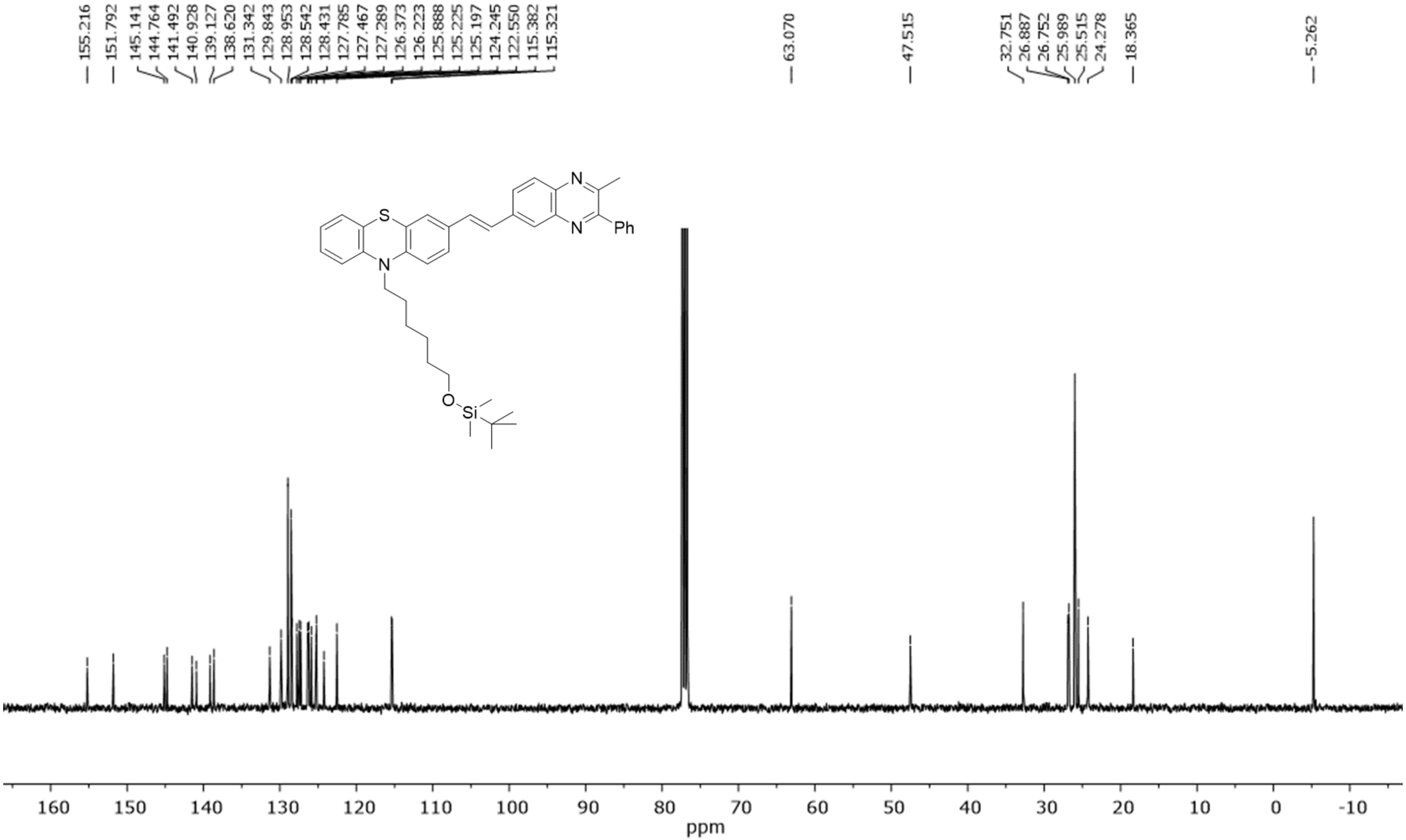

^{13}C NMR ($CDCl_3$, 100 MHz) of (E)-10-(6-((tert-butyldimethylsilyl)oxy)hexyl)-3-(2-(2-methyl-3-phenylquinoxalin-6-yl)vinyl)-10H-phenothiazine (**6e**)

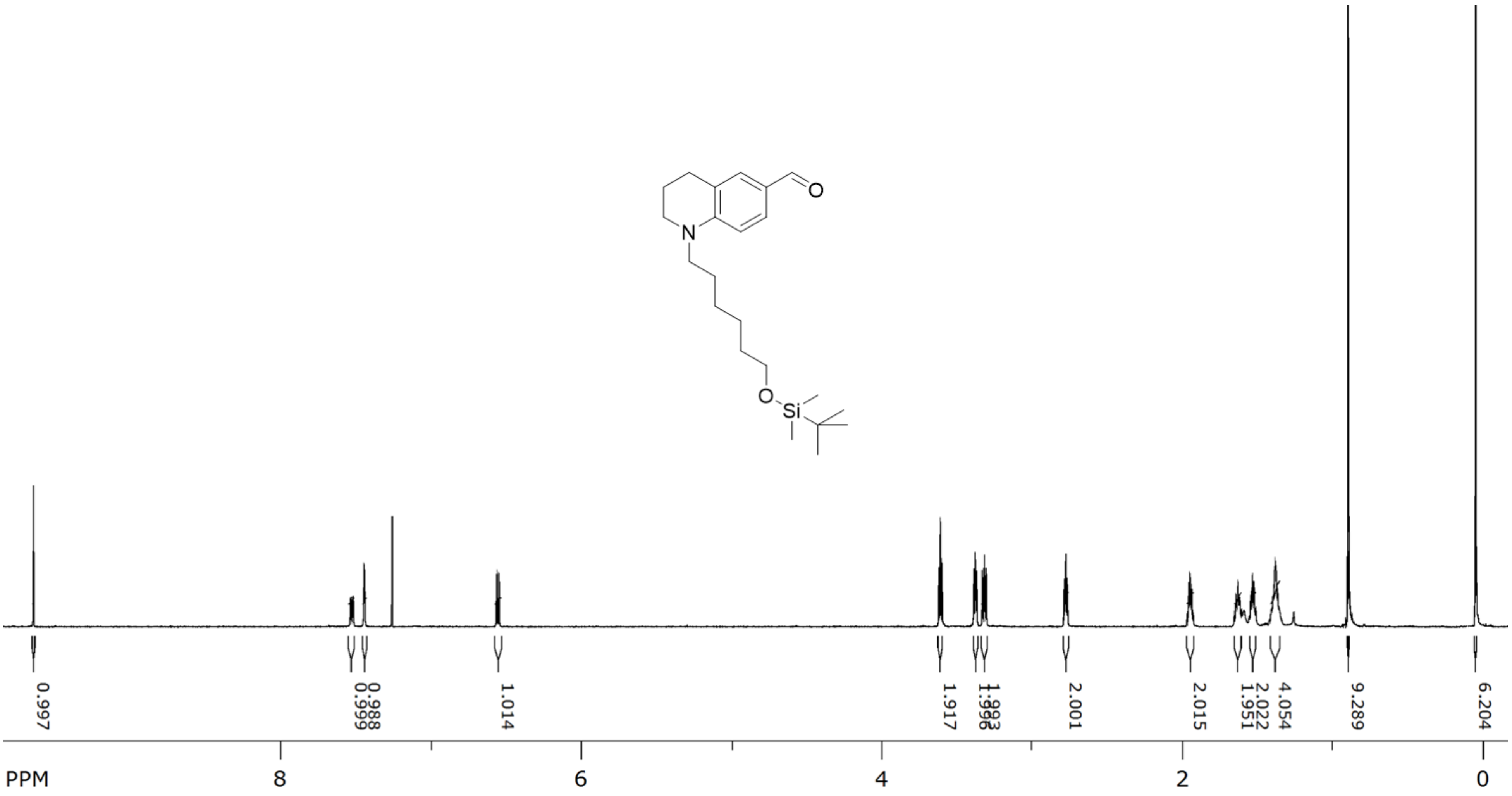

^{1}H NMR ($CDCl_3$, 600 MHz) of 1-(6-((tert-butyldimethylsilyl)oxy)hexyl)-1,2,3,4-tetrahydroquinoline-6-carbaldehyde (**1f'**)

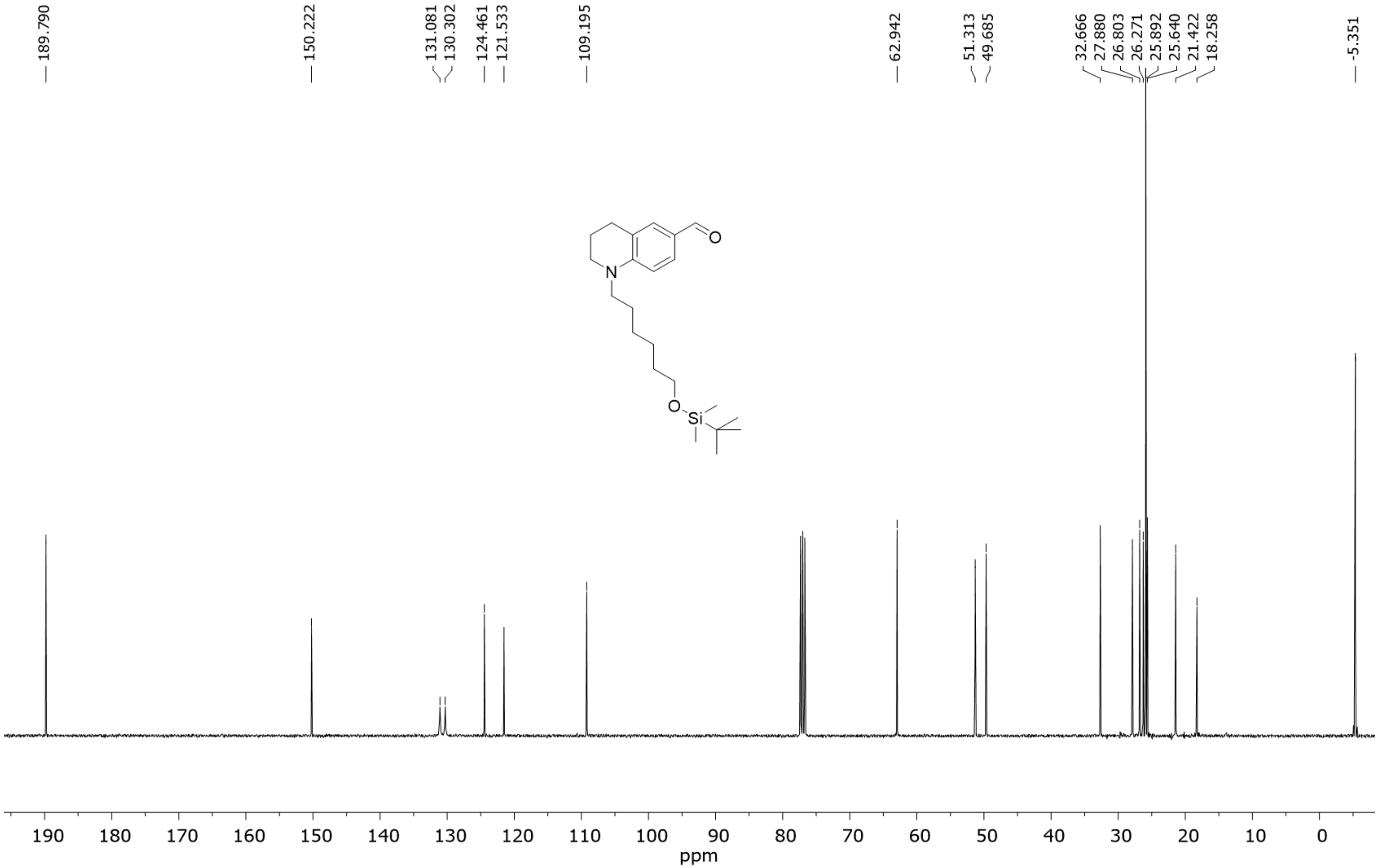

^{13}C NMR ($CDCl_3$, 100 MHz) of 1-(6-((tert-butyldimethylsilyl)oxy)hexyl)-1,2,3,4-tetrahydroquinoline-6-carbaldehyde (**1f'**)

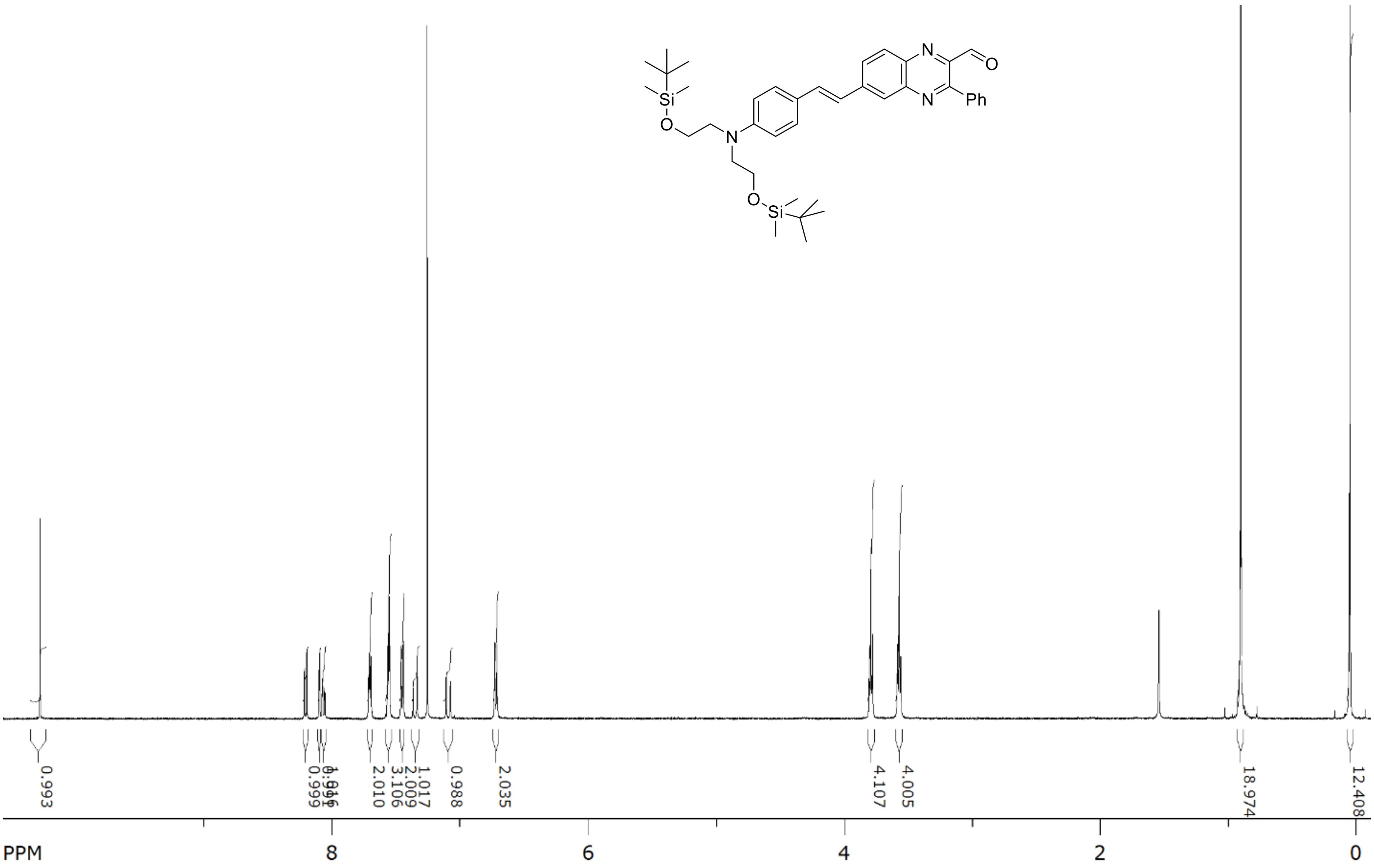

^{1}H NMR ($CDCl_3$, 500 MHz) of (*E*)-6-(4-(bis(2-((*tert*-butyldimethylsilyl)oxy)ethyl)amino)styryl)-3-phenylquinoxaline-2-carbaldehyde (**7b**)

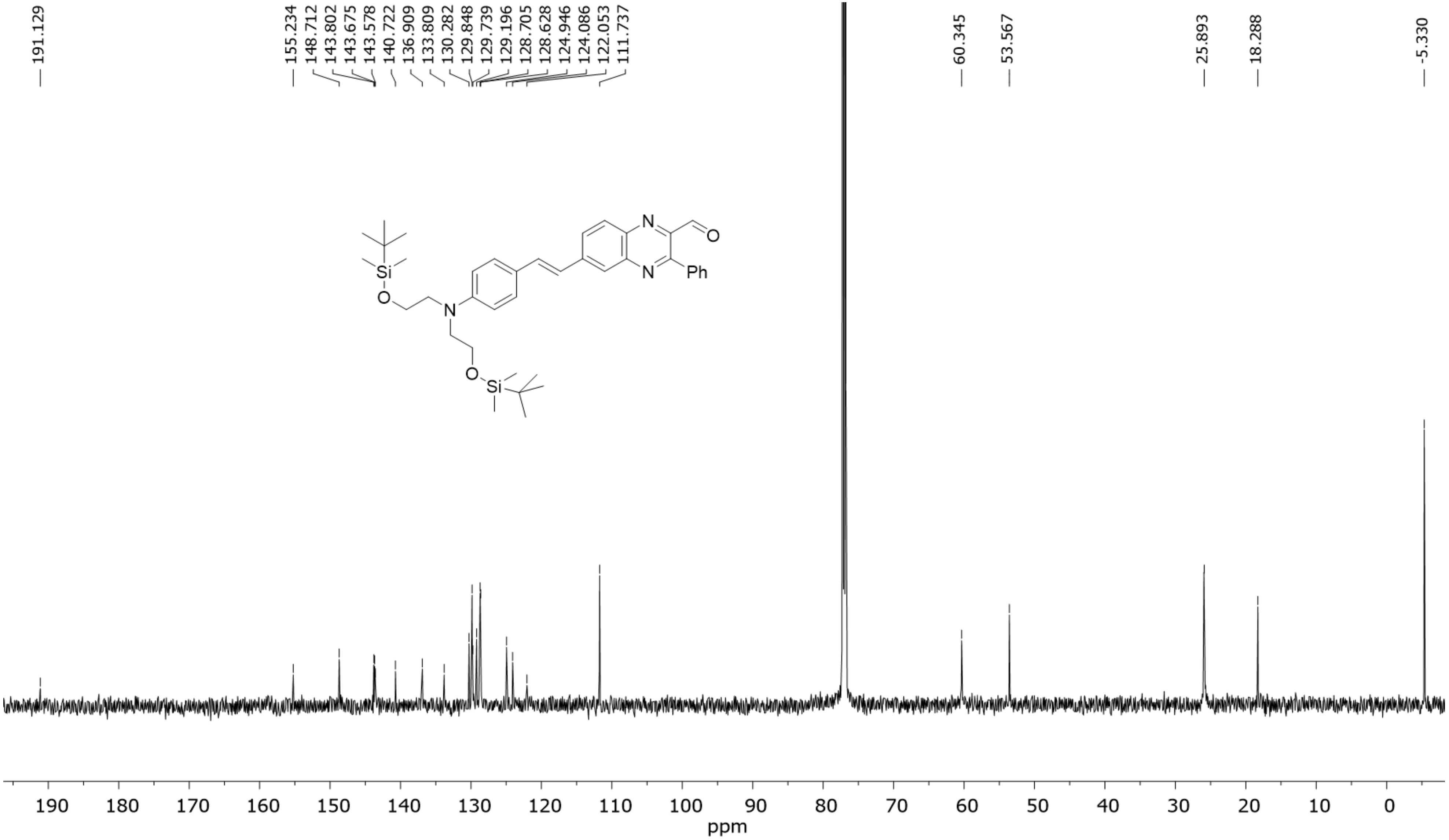

^{13}C NMR ($CDCl_3$, 150 MHz) of (*E*)-6-(4-(bis(2-((*tert*-butyldimethylsilyl)oxy)ethyl)amino)styryl)-3-phenylquinoxaline-2-carbaldehyde (**7b**)

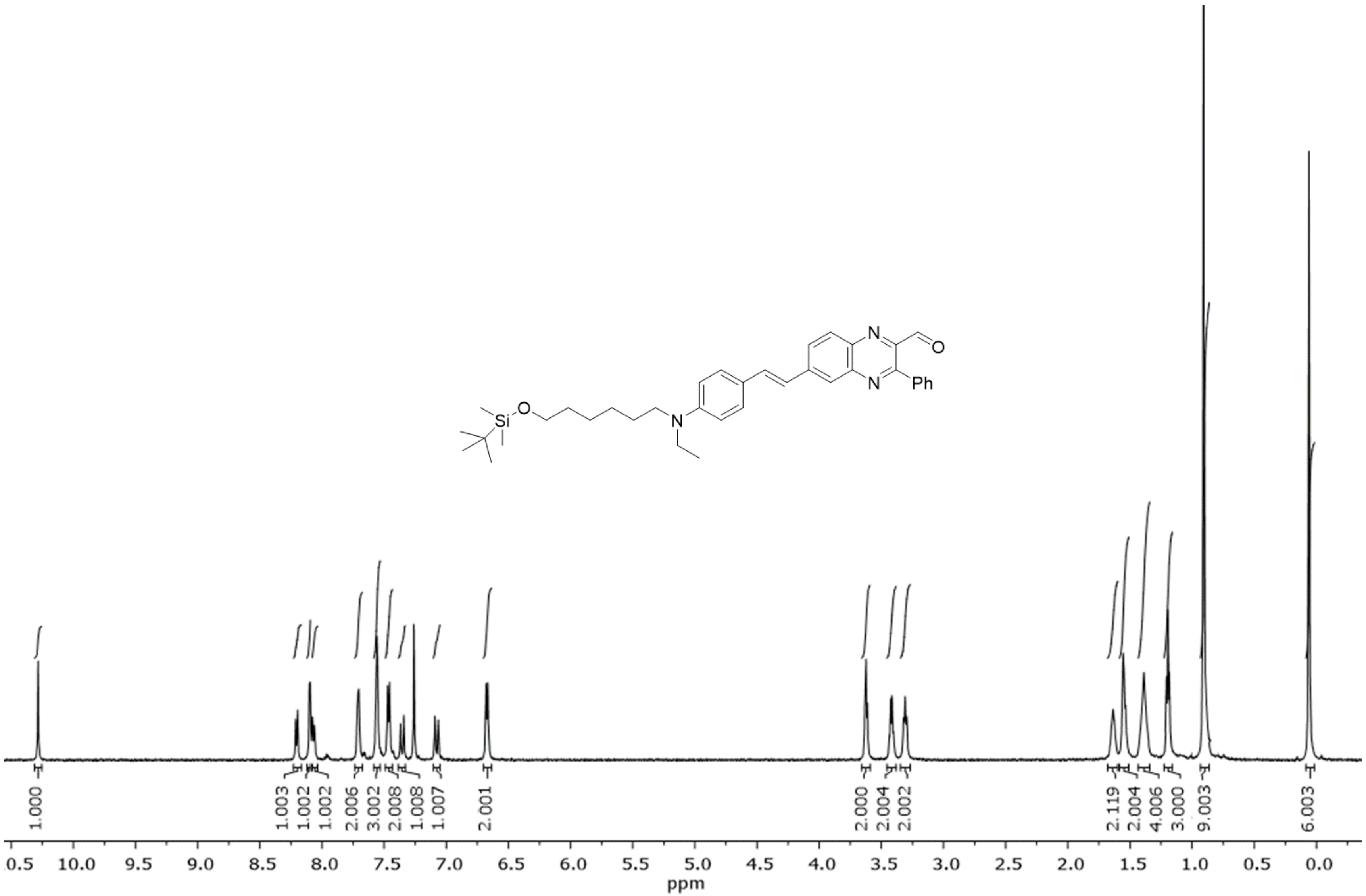

^{1}H NMR ($CDCl_3$, 600 MHz) of (E)-6-(4-((6-((tert-butyldimethylsilyl)oxy)hexyl)(ethyl)amino)styryl)-3-phenylquinoxaline-2-carbaldehyde (**7c**)

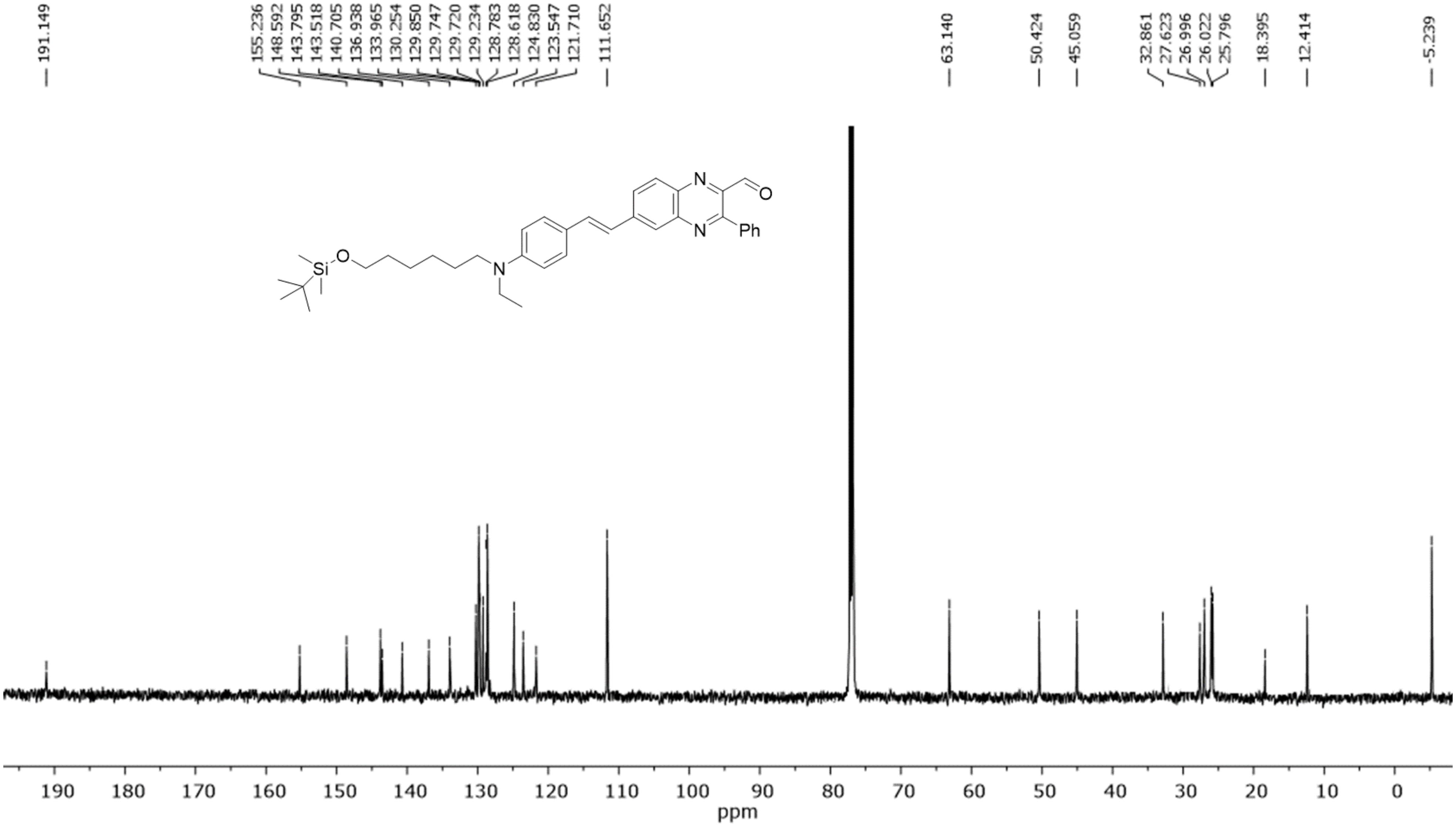

^{13}C NMR ($CDCl_3$, 150 MHz) of (E)-6-(4-((6-((tert-butyldimethylsilyl)oxy)hexyl)(ethyl)amino)styryl)-3-phenylquinoxaline-2-carbaldehyde (**7c**)

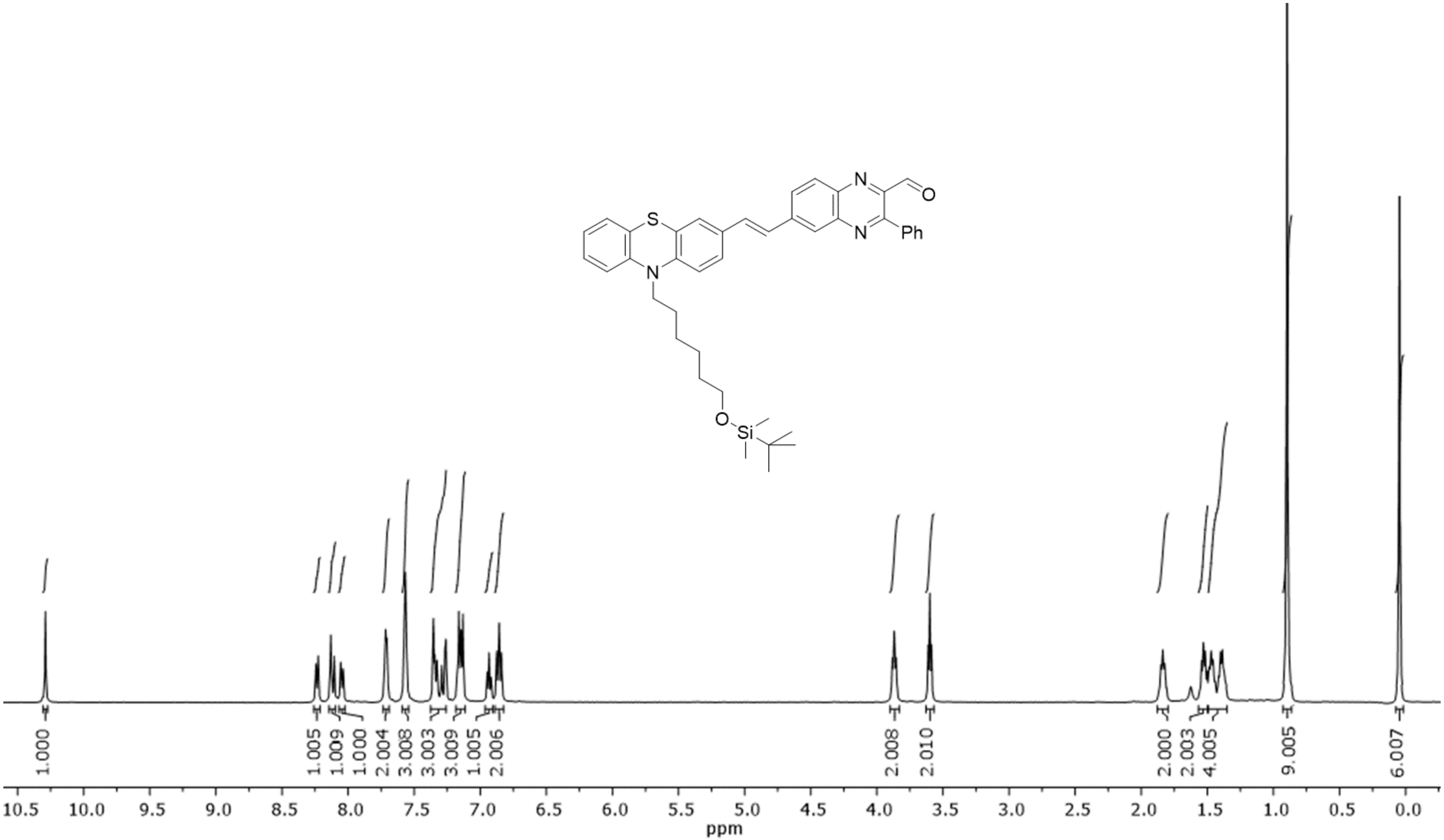

^{1}H NMR ($CDCl_3$, 500 MHz) of (E)-6-(2-(10-(6-((tert-butyldimethylsilyl)oxy)hexyl)-10H-phenothiazin-3-yl)vinyl)-3-phenylquinoxaline-2-carbaldehyde (**7e**)

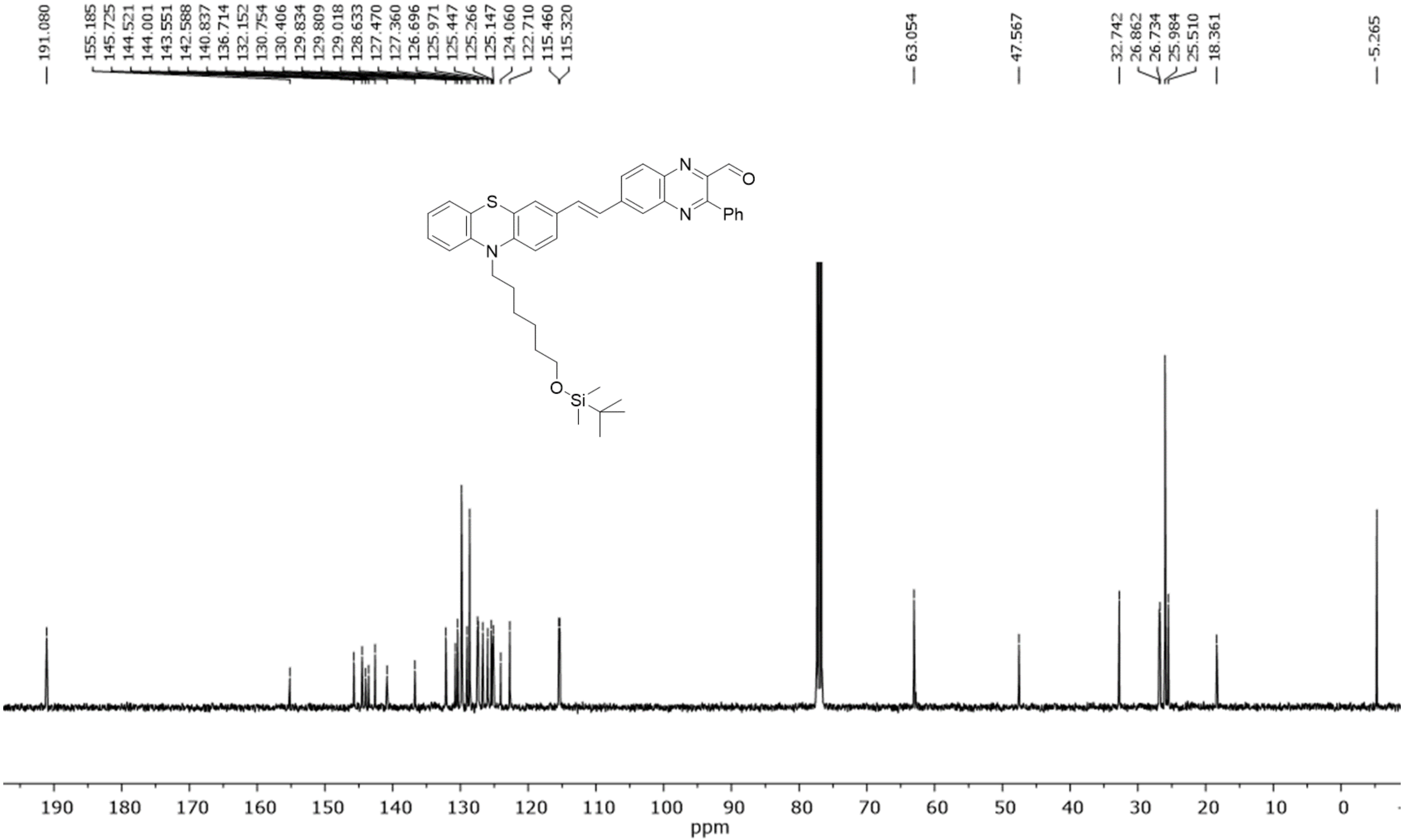

^{13}C NMR ($CDCl_3$, 100 MHz) of (E)-6-(2-(10-(6-((tert-butyldimethylsilyl)oxy)hexyl)-10H-phenothiazin-3-yl)vinyl)-3-phenylquinoxaline-2-carbaldehyde (**7e**)

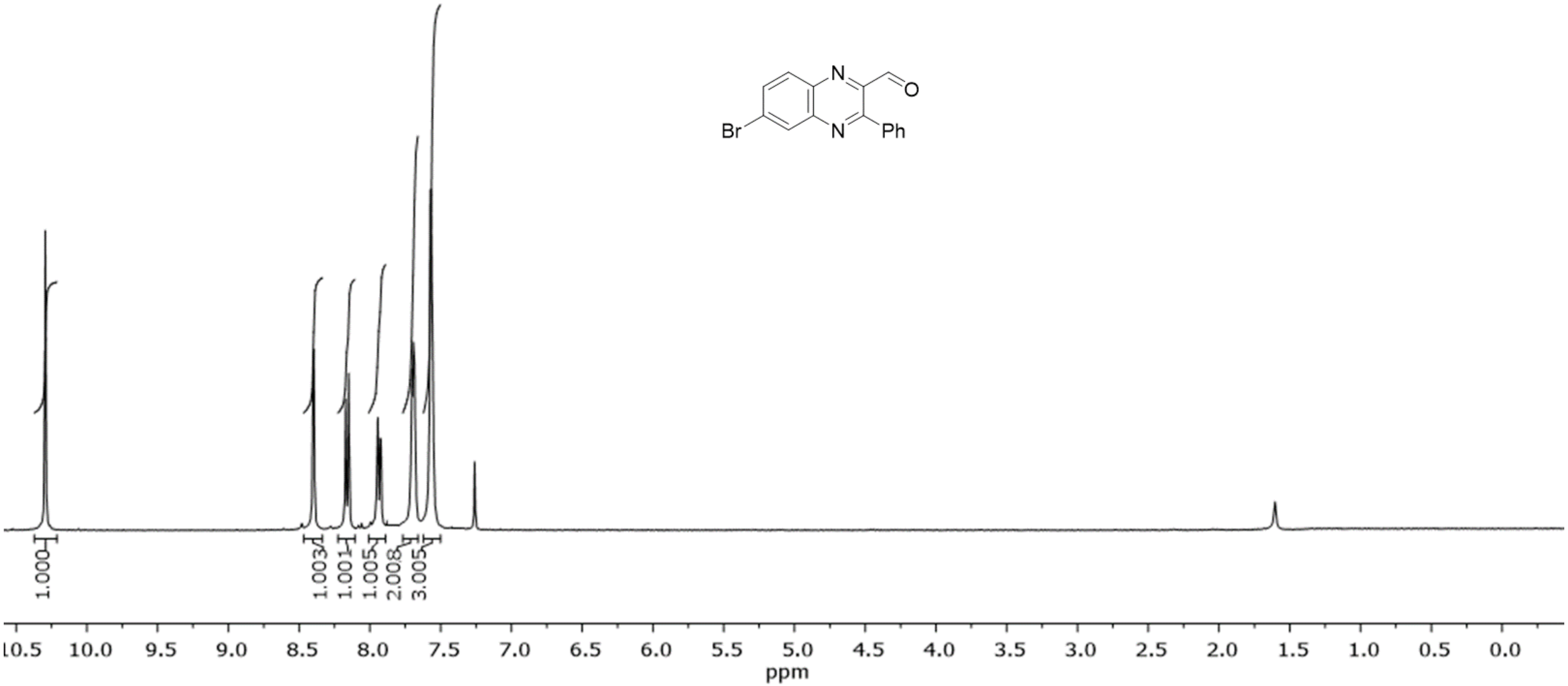

^{1}H NMR ($CDCl_3$, 400 MHz) of 6-bromo-3-phenylquinoxaline-2-carbaldehyde (**3'**)

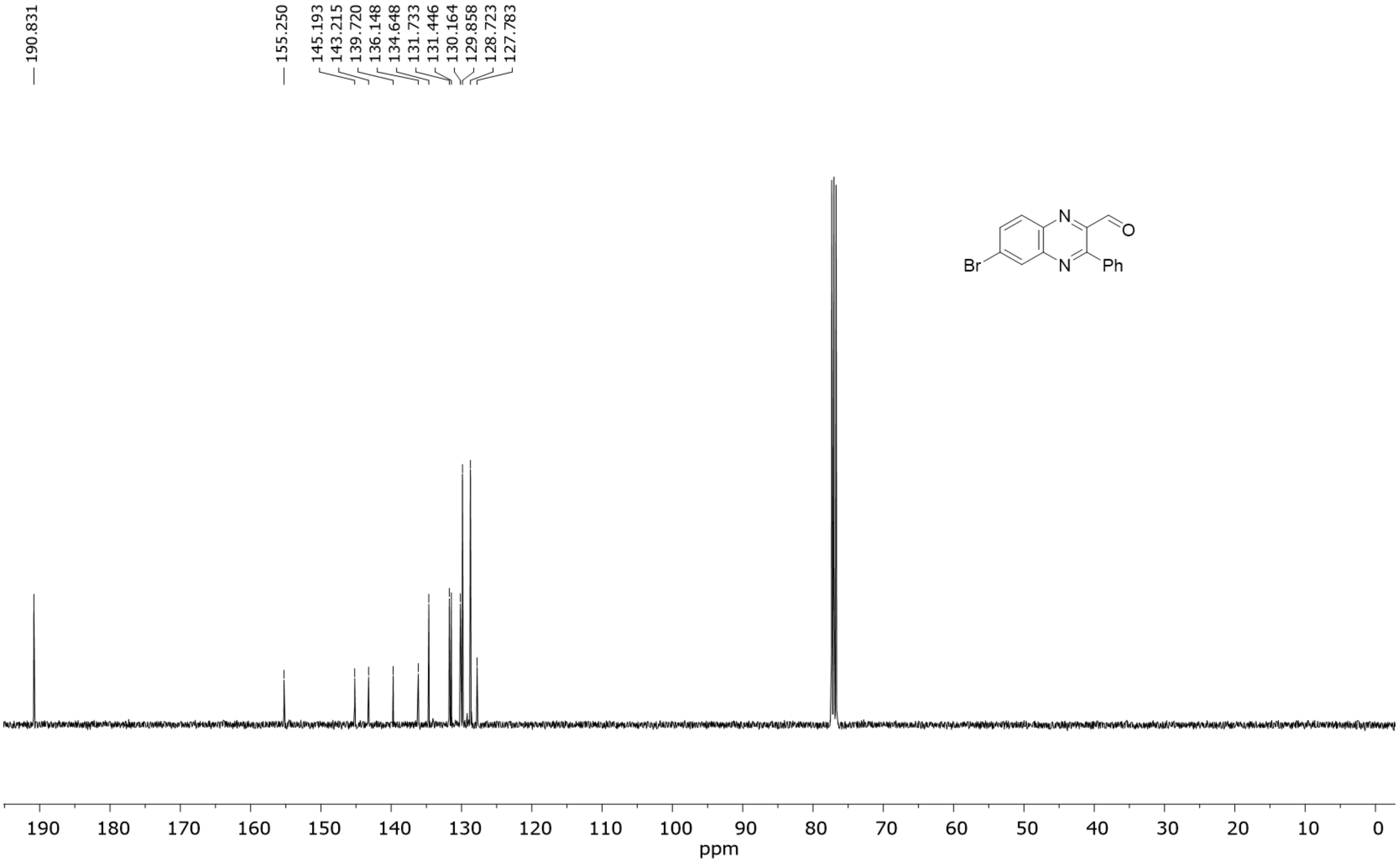

^{13}C NMR ($CDCl_3$, 100 MHz) of 6-bromo-3-phenylquinoxaline-2-carbaldehyde (**3'**)

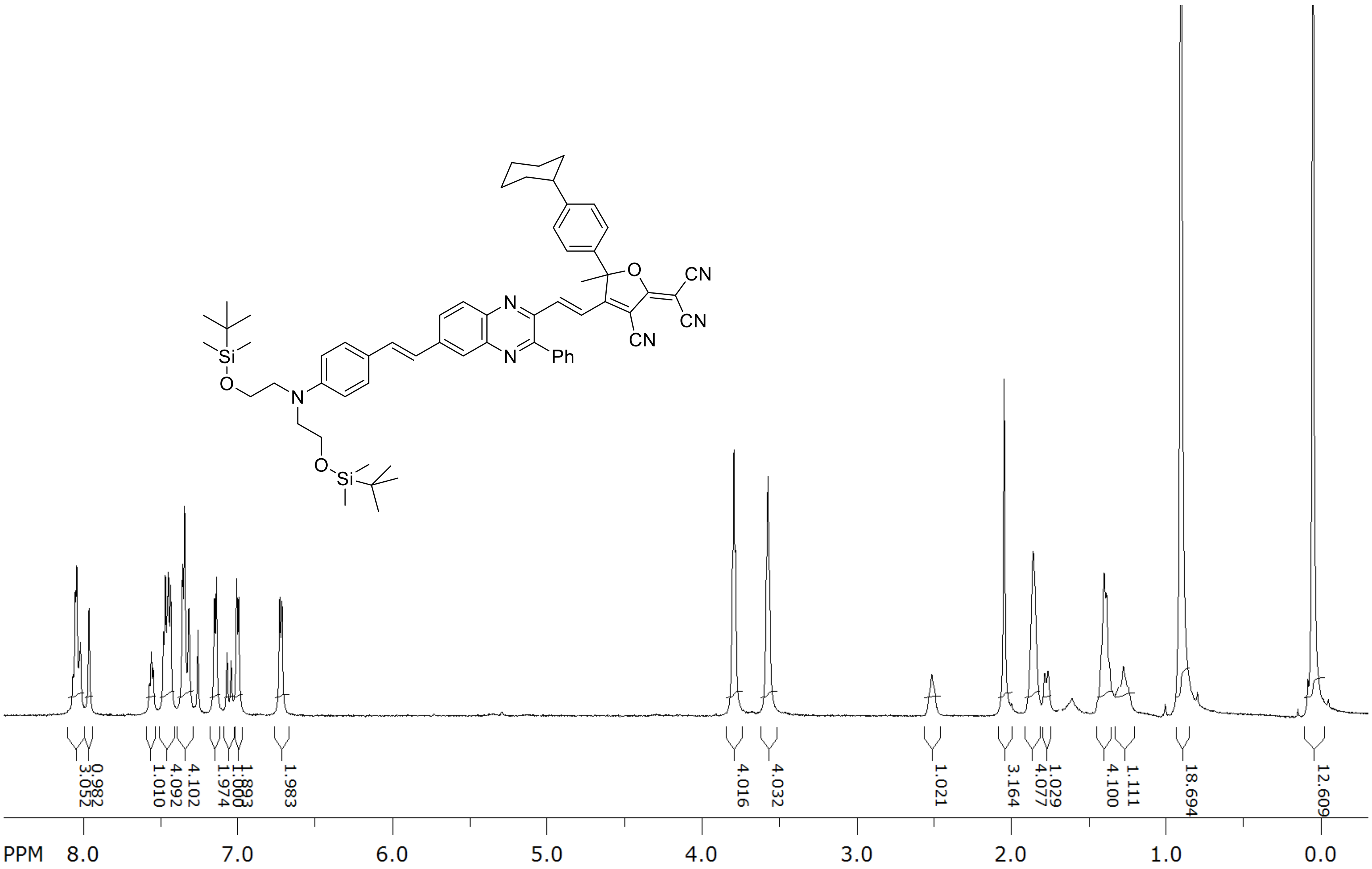

^{1}H NMR ($CDCl_3$, 600 MHz) of 2-(4-((E)-2-(6-((E)-4-(bis(2-((tert-butyldimethylsilyl)oxy)ethyl)amino)styryl)-3-phenylquinoxalin-2-yl)vinyl)-3-cyano-5-(4-cyclohexylphenyl)-5-methylfuran-2(5H)-ylidene)malononitrile (**Ch-An2**).

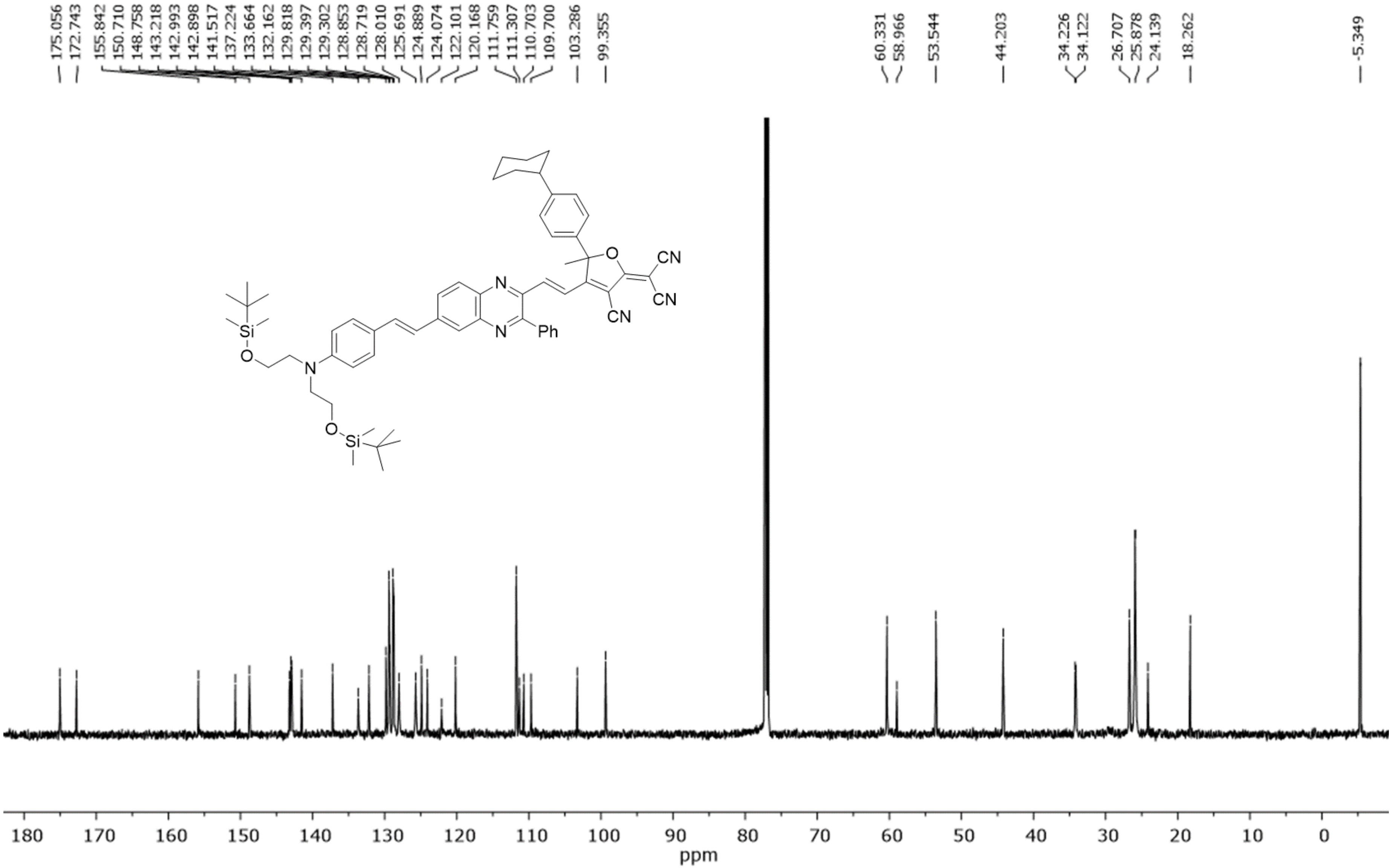

^{13}C NMR ($CDCl_3$, 150 MHz) of 2-(4-((E)-2-(6-((E)-4-(bis(2-((tert-butyldimethylsilyl)oxy)ethyl)amino)styryl)-3-phenylquinoxalin-2-yl)vinyl)-3-cyano-5-(4-cyclohexylphenyl)-5-methylfuran-2(5H)-ylidene)malononitrile (**Ch-An2**)

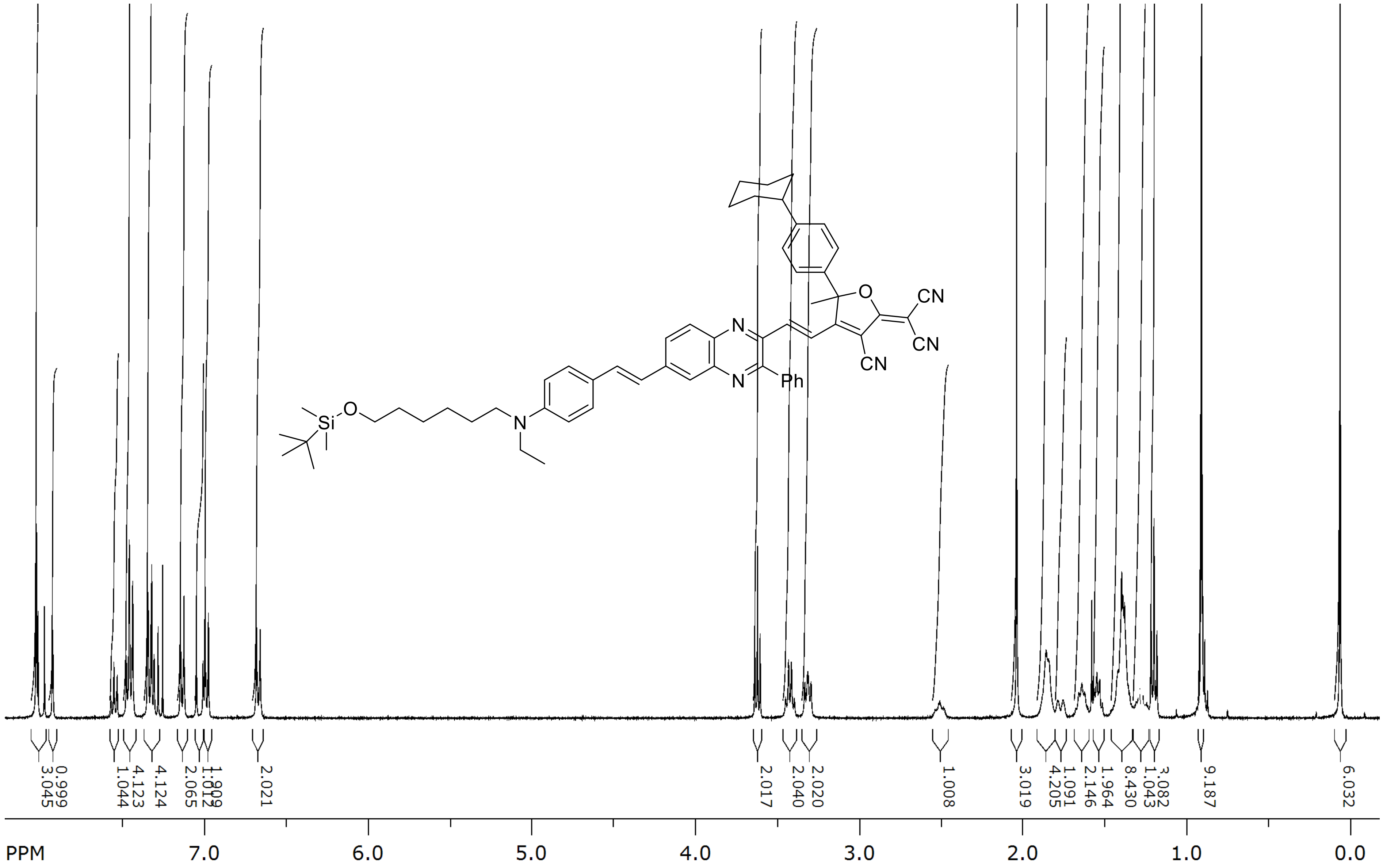

^{1}H NMR ($CDCl_3$, 400 MHz) of 2-(4-((E)-2-(6-((E)-4-((6-((tert-butyldimethylsilyl)oxy)hexyl)(ethyl)amino)styryl)-3-phenylquinoxalin-2-yl)vinyl)-3-cyano-5-(4-cyclohexylphenyl)-5-methylfuran-2(5H)-ylidene)malononitrile (**Ch-An3**)

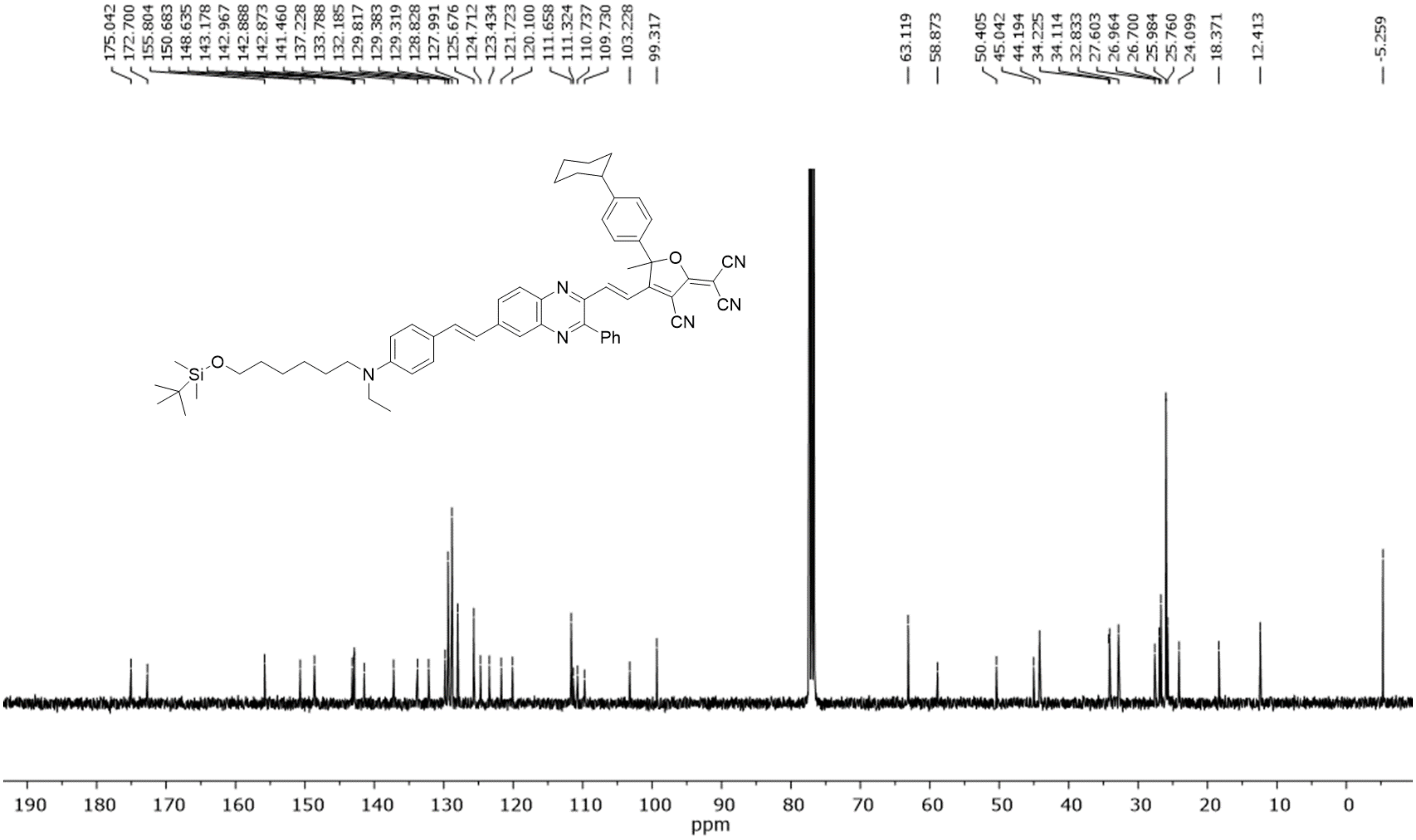

^{13}C NMR ($CDCl_3$, 100 MHz) of 2-(4-((E)-2-(6-((E)-4-((6-((tert-butyldimethylsilyl)oxy)hexyl)(ethyl)amino)styryl)-3-phenylquinoxalin-2-yl)vinyl)-3-cyano-5-(4-cyclohexylphenyl)-5-methylfuran-2(5H)-ylidene)malononitrile (**Ch-An3**)

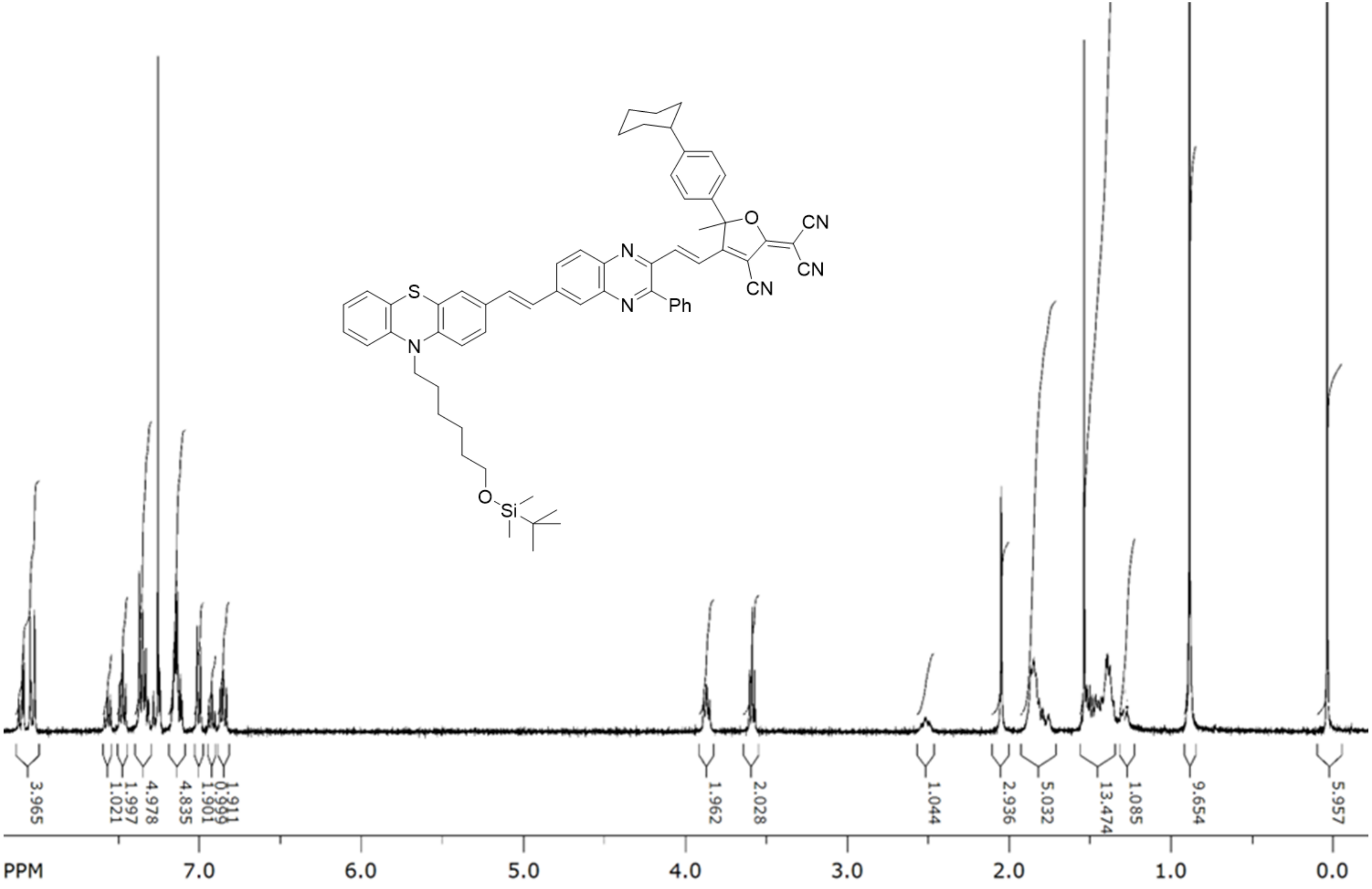

^{1}H NMR ($CDCl_3$, 400 MHz) of 2-(4-((E)-2-(6-((E)-2-(10-(6-((tert-butyldimethylsilyl)oxy)hexyl)-10H-phenothiazin-3-yl)vinyl)-3-phenylquinoxalin-2-yl)vinyl)-3-cyano-5-(4-cyclohexylphenyl)-5-methylfuran-2(5H)-ylidene)malononitrile (**Ch-PT**)

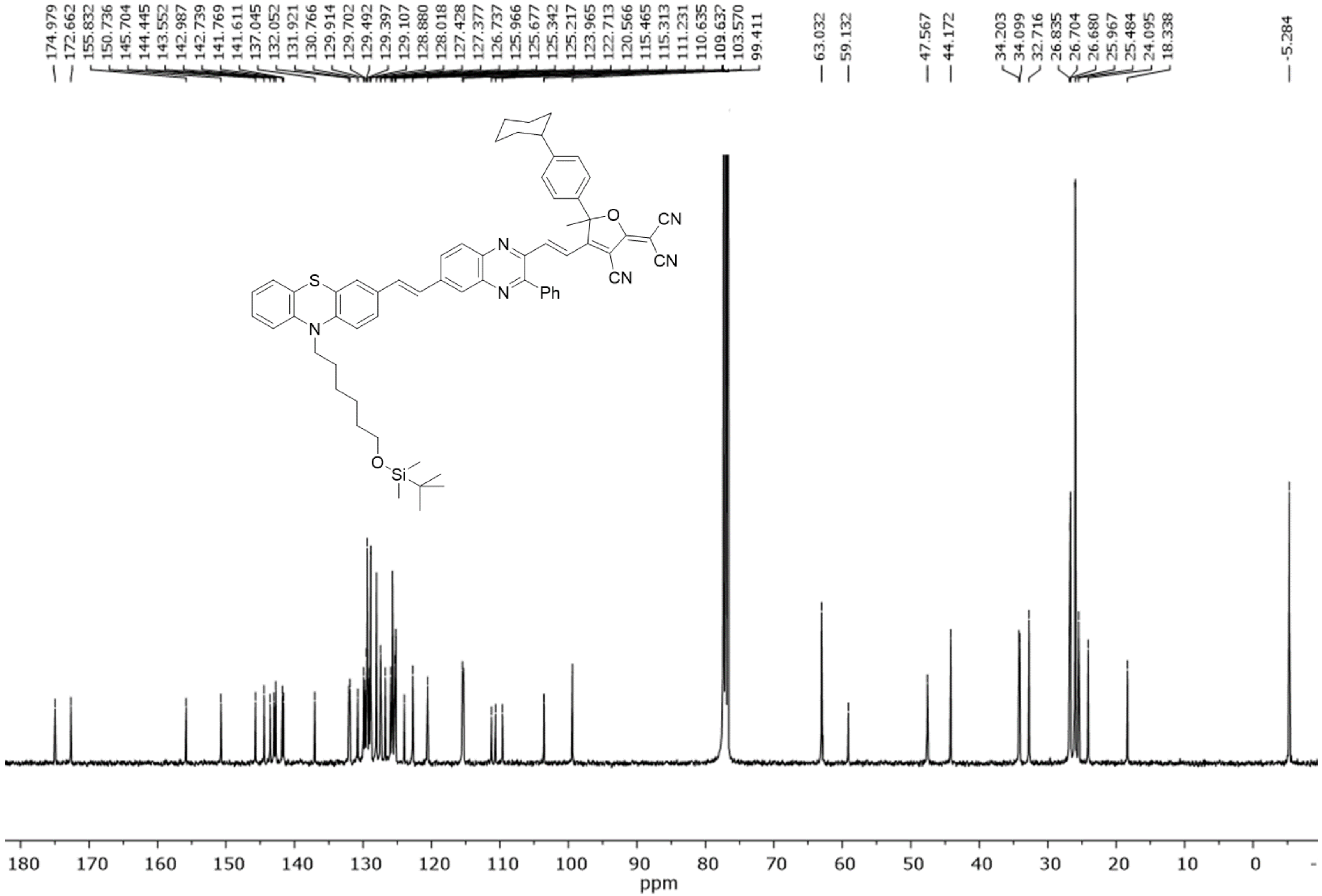

^{13}C NMR ($CDCl_3$, 100 MHz) of 2-(4-((E)-2-(6-((E)-2-(10-(6-((tert-butyldimethylsilyl)oxy)hexyl)-10H-phenothiazin-3-yl)vinyl)-3-phenylquinoxalin-2-yl)vinyl)-3-cyano-5-(4-cyclohexylphenyl)-5-methylfuran-2(5H)-ylidene)malononitrile (**Ch-PT**)

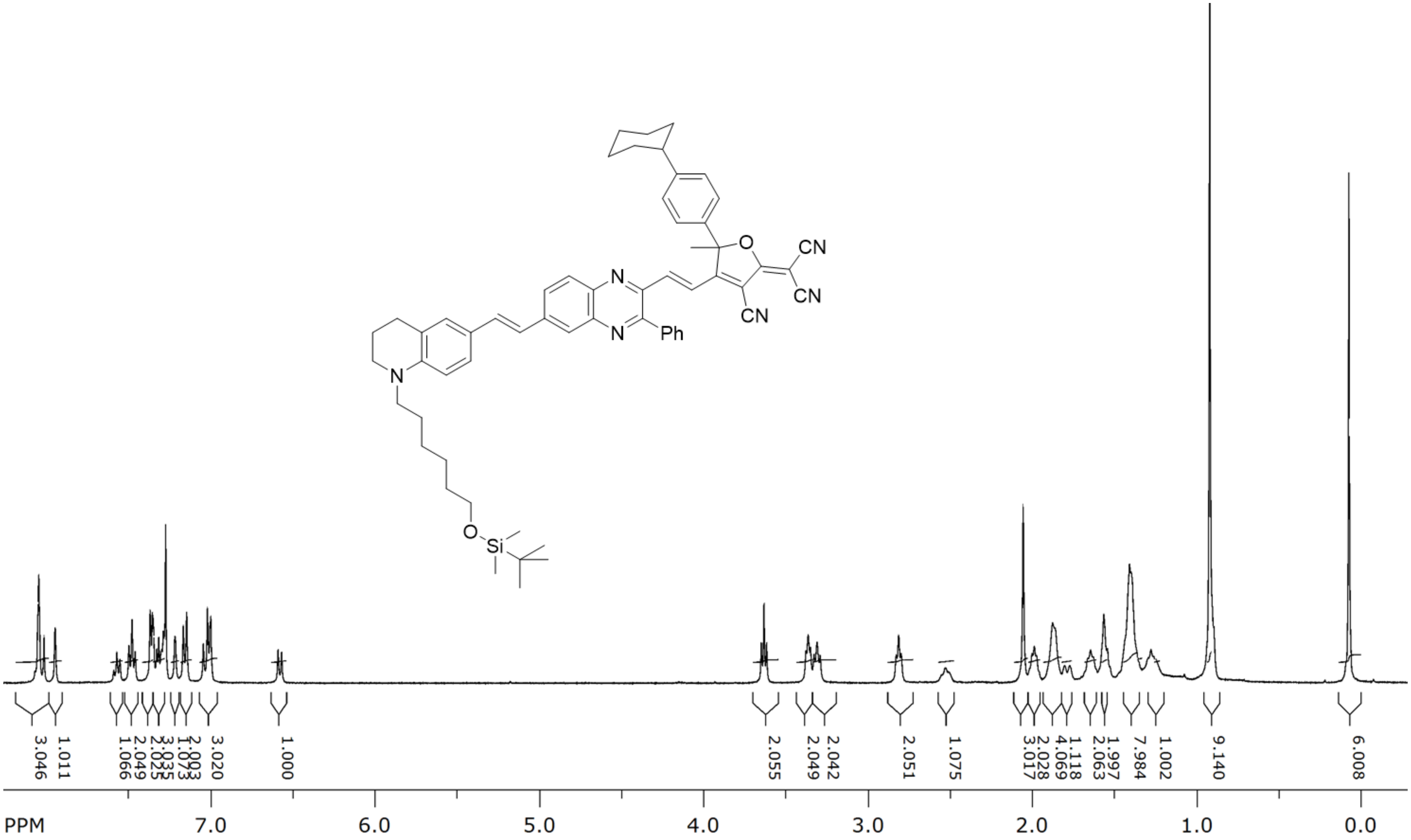

^{1}H NMR ($CDCl_3$, 400 MHz) of 2-(4-((E)-2-(6-((E)-2-(1-(6-((tert-butyldimethylsilyl)oxy)hexyl)-1,2,3,4-tetrahydroquinolin-6-yl)vinyl)-3-phenylquinoxalin-2-yl)vinyl)-3-cyano-5-(4-cyclohexylphenyl)-5-methylfuran-2(5H)-ylidene)malononitrile (**Ch-TQ**)

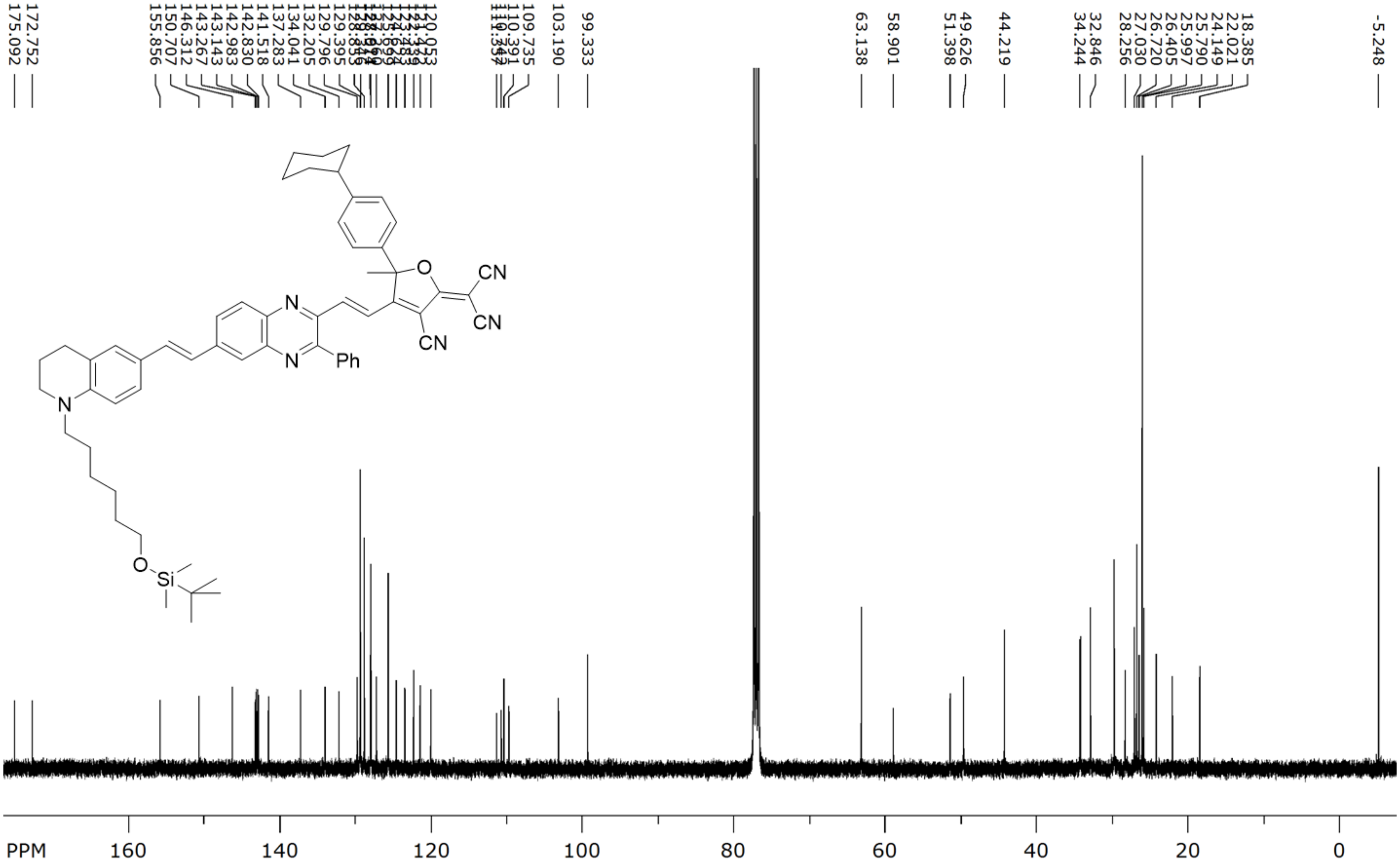

^{13}C NMR ($CDCl_3$, 100 MHz) of 2-(4-((E)-2-(6-((E)-2-(1-(6-((tert-butyldimethylsilyl)oxy)hexyl)-1,2,3,4-tetrahydroquinolin-6-yl)vinyl)-3-phenylquinoxalin-2-yl)vinyl)-3-cyano-5-(4-cyclohexylphenyl)-5-methylfuran-2(5H)-ylidene)malononitrile (**Ch-TQ**)
